# High-Density SNP Screening of the Major Histocompatibility Complex in Systemic Lupus Erythematosus Demonstrates Strong Evidence for Independent Susceptibility Regions

**DOI:** 10.1371/journal.pgen.1000696

**Published:** 2009-10-23

**Authors:** Lisa F. Barcellos, Suzanne L. May, Patricia P. Ramsay, Hong L. Quach, Julie A. Lane, Joanne Nititham, Janelle A. Noble, Kimberly E. Taylor, Diana L. Quach, Sharon A. Chung, Jennifer A. Kelly, Kathy L. Moser, Timothy W. Behrens, Michael F. Seldin, Glenys Thomson, John B. Harley, Patrick M. Gaffney, Lindsey A. Criswell

**Affiliations:** 1 Division of Epidemiology, School of Public Health, University of California Berkeley, Berkeley, California, United States of America; 2 Children's Hospital of Oakland Research Institute, Oakland, California, United States of America; 3 Rosalind Russell Medical Research Center for Arthritis, University of California San Francisco, San Francisco, California, United States of America; 4 Oklahoma Medical Research Foundation, Oklahoma City, Oklahoma, United States of America; 5 Immunology Diagnostics and Biomarkers, Genentech, South San Francisco, California, United States of America; 6 University of California Davis, Davis, California, United States of America; 7 Department of Integrative Biology, University of California Berkeley, Berkeley, California, United States of America; The Jackson Laboratory, United States of America

## Abstract

A substantial genetic contribution to systemic lupus erythematosus (SLE) risk is conferred by major histocompatibility complex (MHC) gene(s) on chromosome 6p21. Previous studies in SLE have lacked statistical power and genetic resolution to fully define MHC influences. We characterized 1,610 Caucasian SLE cases and 1,470 parents for 1,974 MHC SNPs, the highly polymorphic *HLA-DRB1* locus, and a panel of ancestry informative markers. Single-marker analyses revealed strong signals for SNPs within several MHC regions, as well as with *HLA-DRB1* (global p = 9.99×10^−16^). The most strongly associated *DRB1* alleles were: *0301 (odds ratio, OR = 2.21, p = 2.53×10^−12^), *1401 (OR = 0.50, p = 0.0002), and *1501 (OR = 1.39, p = 0.0032). The MHC region SNP demonstrating the strongest evidence of association with SLE was rs3117103, with OR = 2.44 and p = 2.80×10^−13^. Conditional haplotype and stepwise logistic regression analyses identified strong evidence for association between SLE and the extended class I, class I, class III, class II, and the extended class II MHC regions. Sequential removal of SLE–associated *DRB1* haplotypes revealed independent effects due to variation within *OR2H2* (extended class I, rs362521, p = 0.006), *CREBL1* (class III, rs8283, p = 0.01), and *DQB2* (class II, rs7769979, p = 0.003, and rs10947345, p = 0.0004). Further, conditional haplotype analyses demonstrated that variation within *MICB* (class I, rs3828903, p = 0.006) also contributes to SLE risk independent of HLA-DRB1*0301. Our results for the first time delineate with high resolution several MHC regions with independent contributions to SLE risk. We provide a list of candidate variants based on biologic and functional considerations that may be causally related to SLE risk and warrant further investigation.

## Author Summary

Systemic lupus erythematosus (SLE) is an autoimmune disease characterized by autoantibody production and involvement of multiple organ systems. Although the cause of SLE remains unknown, several lines of evidence underscore the importance of genetic factors. As is true for most autoimmune diseases, a substantial genetic contribution to disease risk is conferred by major histocompatibility complex (MHC) gene(s) on chromosome 6. This region of the genome contains a large number of genes that participate in the immune response. However, the full contribution of this genomic region to SLE risk has not yet been defined. In the current study we characterize a large number of SLE patients and family members for approximately 2,000 MHC region variants to identify the specific genes that influence disease risk. Our results, for the first time, implicate four different MHC regions in SLE risk. We provide a list of candidate variants based on biologic and functional considerations that may be causally related to SLE risk and warrant further investigation.

## Introduction

Systemic lupus erythematosus (SLE) is the prototypic systemic autoimmune disease characterized by autoantibody production and involvement of multiple organ systems. Although the etiology of SLE remains unknown, several lines of evidence underscore the importance of genetic factors, including a high sibling risk ratio (λ_s_ = 8–29), familial clustering, where approximately 10 to 12% of SLE patients have an affected first-degree relative, and higher concordance rates in monozygotic twins (24–69%) relative to dizygotic twins and non-twin siblings (2–9%) [1],[2],[3].

Similar to many other autoimmune diseases, genes within the major histocompatibility complex (MHC) on the short arm of chromosome 6p21.3 exhibit strong association with the risk for SLE.

The MHC is a gene-dense region of the human genome spanning approximately 4.5 Mb and known to encode more than 180 expressed genes [4],[5]. Forty percent of the expressed loci have functions related to immune activation and response. The class III region, with more than 55 expressed loci, is the most dense subregion of the MHC and the entire human genome [6].

Historically, interest in the MHC region for SLE has focused on the highly polymorphic HLA class I and II genes that encode membrane glycoproteins that present peptides for recognition by T lymphocytes, as well as genes within the HLA class III region, particularly the tumor necrosis factor and complement component C4 gene loci. Indeed, inherited deficiency of complement genes, particularly C4A (null) alleles, has long been recognized as strong, albeit rare, genetic risk factors for SLE [7]. Work by Graham and colleagues [8],[9] examining ∼50 microsatellite markers across the MHC region highlighted the importance of HLA class II haplotypes involving the *HLA-DRB1* and –*DQB1* loci, particularly those corresponding to serologic types HLA-DR2 and DR3 (DRB1*1501-DQB1*0602 and DRB1*0301-DQB1*0201, respectively). More recently, genome wide association scans in SLE also underscore the importance of the MHC [10],[11],[12]. However, strong linkage disequilibrium (LD) between particular alleles within the MHC has interfered with disease variant identification and previous studies have not been able to distinguish between associated MHC variants. Long-range, extended or ‘ancestral’ haplotypes, sometimes spanning greater than 2 Mb, have been observed [8],[13]. Recent studies in European-derived populations have examined the distribution of LD across the MHC and have suggested that SNPs can help dissect causal variation within this region [14],[15].

A recent analysis of 314 UK SLE families examining 68 SNPs across the HLA class II and III regions and *HLA-DRB1* suggested two distinct association signals centered on DRB1*0301 and the T allele of rs419788 in intron 6 of the class III region gene *SKIV2L* [16]. Of interest, the class III signal appears to exclude the *TNF -308* promoter polymorphism, which has been a focus of prior work [17]. Herein we extend this work by characterizing a much larger collection of SLE cases (and parents) for 1,974 MHC genetic markers plus the *HLA-DRB1* locus. We also characterized SLE cases for a set of ancestry informative markers (AIMs) to identify population outliers and assess the possible impact of substructure within the European population on our genetic association results. Further, we utilized several analytic strategies to determine whether multiple distinct alleles or haplotypes contribute to SLE risk.

## Results/Discussion

Table 1 summarizes characteristics of the 1,610 Caucasian SLE cases and available parents (n = 1,470) from the complete trio families included in this study. Clinical characteristics of the SLE cases are consistent with those reported for Caucasian patients, generally [18].

**Table 1:** Demographic and clinical characteristics of study subjects.

Case Series (# SLE cases)*	# trio families (# parents)
UCSF (925)	325 (650)
OMRF (429)	170 (340)
UMN (256)	240 (480)
Total # SLE cases: 1,610	Total # parents: 1,470
Female, %	92
Age at SLE diagnosis, mean (standard deviation)	33.1 (13.2)
History of renal involvement†, %	32
dsDNA autoantibody production‡, %	54
Ethnicity (based on ancestry informative marker data)	1,577 of 1,610 successful
≥80% European ancestry	1,518 (96.3%)
≥90% European ancestry	1,392 (88.3%)
≥90% Northern European ancestry (estimated for those with ≥90% European ancestry)	1,130 (71.1%)

*: UCSF = University of California, San Francisco; OMRF = Oklahoma Medical Research Foundation, UMN = University of Minnesota.

**†:** renal involvement based on presence of American College of Rheumatology renal criterion [35] and/or renal biopsy findings consistent with lupus nephritis.

**‡:** dsDNA = double strand DNA.

Table 2 summarizes association results for *HLA-DRB1* alleles in SLE cases and non-transmitted control alleles from parents of the trio families [19]. Due to the highly polymorphic nature of this locus and prior evidence suggesting that multiple *HLA-DRB1* alleles influence SLE risk, we employed a relative predispositional effects (RPE) analysis [20] (see Methods). Results indicated that three specific *HLA-DRB1* alleles were strongly associated with SLE risk: DRB1*0301 (odds ratio, OR = 2.21, p = 2.53×10^−12^), *1401 (OR = 0.50, p = 0.0002) and *1501 (OR = 1.39, p = 0.0032). The DRB1*0801 allele, which has been shown in previous work to be associated with SLE risk [8] was not significantly associated with SLE in the current RPE analysis, after accounting for testing of multiple *DRB1* alleles (full results shown in Table S1).

**Table 2:** Association of *HLA-DRB1* alleles with SLE risk based on relative predispositional effects (RPE) analysis of 1,522 SLE cases and 693 controls.

*HLA-DRB1* allele	Case allele frequency	Control allele frequency	P-value†	Odds Ratio (95% C.I.)‡
*0301	0.206	0.110	2.53×10^−12^	2.21 (1.82, 2.68)
*1401	0.015	0.035	0.0002	0.50 (0.33, 0.75)
*1501	0.165	0.141	0.0032	1.39 (1.15, 1.66)
*0801	0.031	0.023	0.0293	1.59 (1.05, 2.39)

**†:** P-values based on RPE analysis and therefore account for the other associated *DRB1* alleles (see Methods).

**‡:** Reference group for odds ratio calculations included all *DRB1* alleles except *0301, *0801, *1401, and *1501.

**Figure 1:**
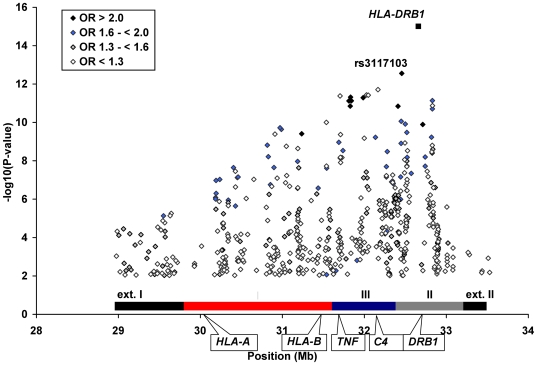
Association of 544 MHC region SNPs (out of 1,974) and *HLA-DRB1* with SLE. Shown are results for variants with p<0.01 among 1,484 SLE cases and 650 controls. UNPHASED v.3.0.10 was used for 1,974 SNPs and SAS v. 9.1.3 for *HLA-DRB1* global p-value. Odds ratio shown for *HLA-DRB1* corresponds to *0301 allele.

Figure 1 displays single marker association results for the 1,974 MHC SNPs (out of 2,360) passing quality control filters (see Methods) in SLE cases compared to controls (additional results are shown in Table S2). We observed strong association signals across a broad region encompassing the HLA class I, III and II regions, with weaker evidence of association in the extended class I and II regions. The strongest evidence of association was observed at the multiallelic *DRB1* locus (global p = 9.99×10^−16^ and ORs as shown in Table 2). The MHC region SNP demonstrating the strongest evidence of association with SLE was rs3117103 (bp 32,457,535), with OR = 2.44 and p = 2.80×10^−13^. This SNP is flanking C6orf10 at a distance of 9.8 kb.

**Figure 2:**
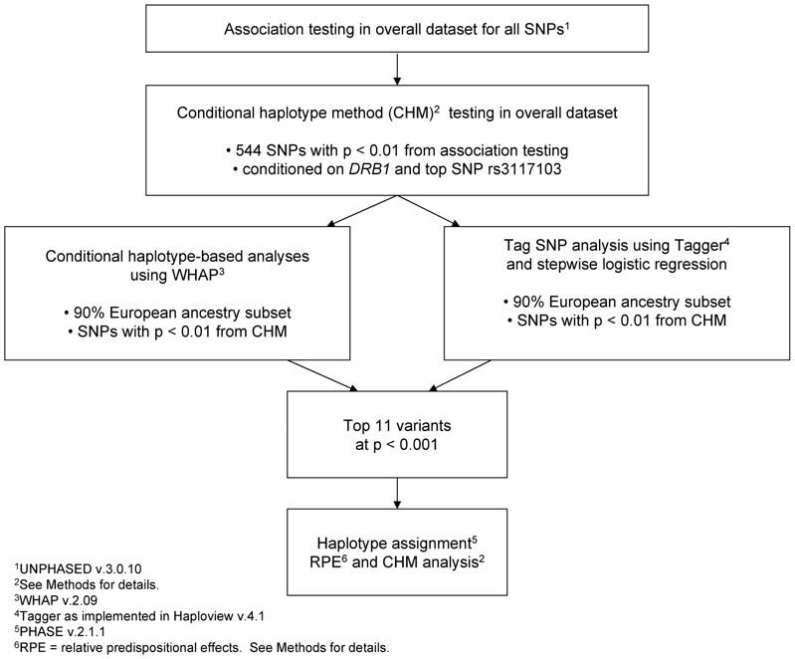
Summary of analytical plan. Summary of analytical plan for defining MHC region genetic variants with independent contributions to SLE risk among 1,484 SLE cases and 650 controls.

Given the extensive LD across this region, we utilized conditional analyses to identify association signals that were not due to rs3117103. Similarly, we repeated these analyses conditioning instead on the *HLA-DRB1* locus, including the DRB1*0301, *1401 and *1501 alleles. The rs number and map position for all SNPs associated with SLE using the conditional haplotype method (CHM) (total = 171 SNPs with p<0.01) are provided in Table S3. Following these conditional analyses, several analytic approaches were utilized to further define genetic variants with the most compelling evidence of association with SLE risk, as shown in Figure 2.

**Figure 3:**
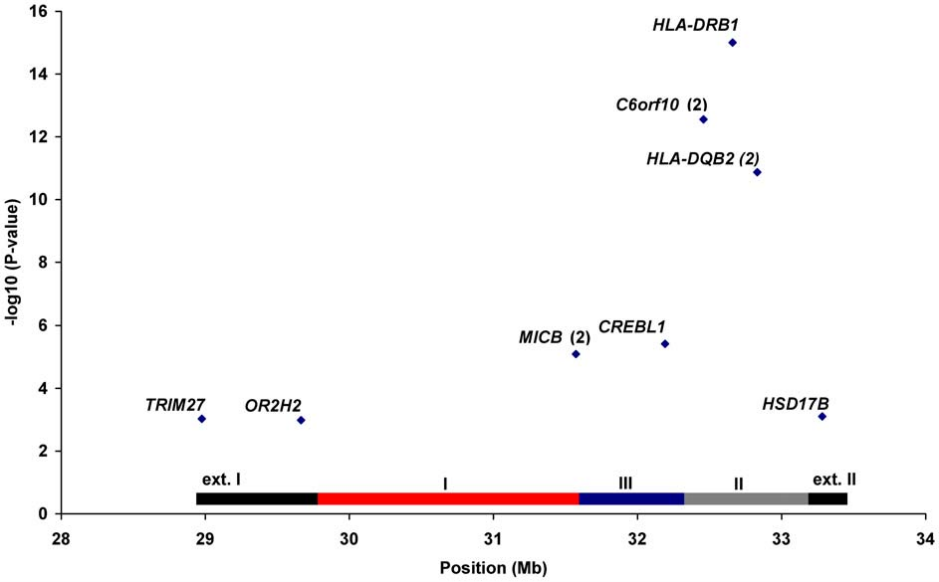
Summary of MHC region variants with evidence of independent association with SLE. Summary of MHC region variants (n = 11, including *HLA-DRB1*) with evidence of independent association with SLE (p<0.001) based on conditional haplotype and stepwise logistic regression analyses. Number in parentheses indicates number of SNPs at that locus. See Figure 2 for details of overall analytical strategy. P-values from original univariate analyses are shown on the y-axis.

Figure 3 displays initial association results for the MHC region variants (n = 11, including *HLA-DRB1*) that were shown to be associated with SLE risk based on conditional haplotype and stepwise logistic regression analyses, with p<0.001 (See Figure 2 and Methods). In addition to the *HLA-DRB1* locus, there were several variants with evidence of association with SLE, including one variant in the extended class II region, five variants in HLA class II (including *HLA-DRB1*), one variant in HLA class III, two in HLA class I, and two in the extended class I region.

**Figure 4:**
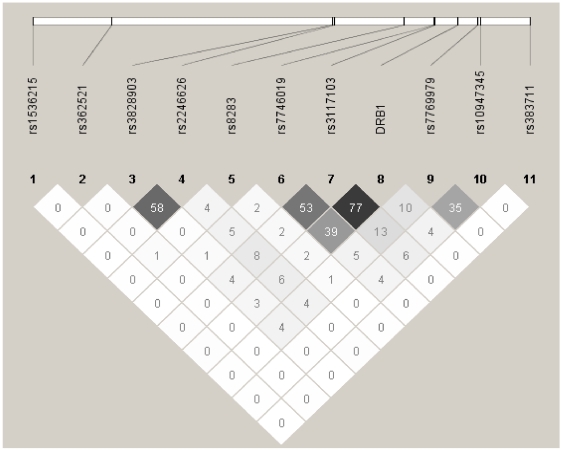
Linkage disequilibrium (LD) plot for *HLA-DRB1* and 10 MHC region SNPs. LD plot showing correlation (r^2^) for *HLA-DRB1* and 10 MHC region SNPs demonstrating evidence of independent association with SLE. A total of 2,607 individuals with >90% European ancestry (maximum unrelated subset of all cases and trios) were used for analysis. Similar results were obtained when only the SLE cases or SLE trio family founders were used to generate the LD plots (data not shown). *HLA-DRB1* was coded as a biallelic marker (DRB1*0301 vs. others) for analysis in Haploview based on results obtained for LD analyses of this locus as a multilocus marker using UNPHASED (see Methods). See Table 3 for other gene names.

Figure 4 shows an LD plot representing the correlation (r^2^) among *HLA-DRB1* and the aforementioned 10 associated SNPs that met our significance threshold of p<0.001 based on conditional analyses (see Methods and Table S4). There was very little evidence of LD among these 10 SNPs and *HLA-DRB1* with the exception of SNPs within the *MICB* (r^2^ = 0.58), *C6orf10* (r^2^ = 0.53) and *HLA-DQB2* loci (r^2^ = 0.35) and strong LD between *HLA-DRB1* and the *C6orf10* locus (r^2^ = 0.77).

To further evaluate the evidence supporting association of these 10 variants with SLE we performed transmission disequilibrium testing [21],[22] among 650 complete trio families. These analyses were consistent with the prior results, including strongest evidence of association for rs3117103 (OR = 2.44, p = 1.58×10^−14^). Additional details are shown in Table S5.

We also evaluated these 10 SNPs among a more homogeneous subset of cases (and non-transmitted controls) estimated by AIMs to have ≥90% Northern European ancestry. These results also supported our main analyses, with the strongest evidence of association for rs3117103 (OR = 2.49, p = 1.62×10^−13^) (see Table S5 for additional details). Similarly, results for the top four *DRB1* risk alleles did not change when analyses were repeated among the Northern European subset of cases and controls (data not shown).

A distinguishing feature of the MHC region is the extensive, long-range LD, which has been observed particularly among European-derived populations [15] and is haplotype specific [23]. Thus, to further assess the independence of the 11 variants (including *DRB1*) and determine whether one or more extended haplotypes are strongly associated with SLE risk, we estimated haplotypes involving these variants and evaluated the evidence of association with SLE (see Methods). When case and control chromosomes were considered together, a total of 171 unique haplotypes were observed. When MHC haplotypes were compared between SLE cases and controls, evidence of association was revealed for two common haplotypes. The haplotype with the strongest evidence of association with SLE contains the DRB1*0301 allele (15% transmitted vs. 6% non-transmitted haplotypes, OR = 2.63, p = 8.32×10^−15^), which represents ∼70% of the DRB1*0301 haplotypes. The other associated haplotype contains the *1501 allele (9% transmitted vs. 6% non-transmitted haplotypes, OR = 1.54, p = 0.0025) (data not shown).

Finally, we considered our data both a) excluding and b) conditioning on haplotypes containing the SLE-associated *DRB1* alleles. More specifically, we compared MHC SNP allele frequencies among cases vs. controls after sequentially removing haplotypes containing *0301, *1501, and then *1401 (see Table 3 and Table S6). After removing haplotypes containing these alleles, we observed no evidence for independent association of rs3117103 with SLE (p>0.30; see Table 3 and Table S6). However, four MHC region variants remained significantly associated with SLE, including rs362521 (*OR2H2*, extended class I region; p = 0.0060), rs8283 (*CREBL1*, class III; p = 0.011), rs7769979 and rs10947345 (*DQB2*, class II; p = 0.0030 and 0.0004, respectively). Of interest, two HLA class II region SNPs at the *DQB2* locus remained associated with SLE even in the absence of *DRB1* risk alleles. This is also the first study to support a role in SLE for variants within the extended MHC class I region, near *OR2H2*. Next, the CHM was used to investigate associations with the top 10 MHC region SNPs conditioned on haplotypes containing the SLE-associated *HLA-DRB1* alleles (see Table 4 and Table S6). When conditioning on DRB1*0301 haplotypes, we observed significant association with SLE (p = 0.0059) for a SNP at the *MICB* locus (HLA class I). In contrast, conditioning on DRB1*1501 or DRB1*1401 revealed no evidence for other MHC effects on SLE risk, however limited power was present for conditional analyses involving DRB1*1401 given the infrequency of that allele in this dataset. Finally, logistic regression analyses involving the aforementioned five SNPs and *HLA-DRB1* revealed evidence of independent association with SLE for all variants (p-values = 0.003 to <10^−6^) with the exception of one of the *DQB2* SNPs (rs7769979) (data not shown).

**Table 3:** Results from relative predispositional effects analyses of top MHC SNPs in SLE cases and controls.

		All Haps†		DRB1*0301 removed		DRB1*0301, *1501 removed		DRB1*0301, *1501, *1401 removed	
SNP	Gene	P-value	OR	P-value	OR	P-value	OR	P-value	OR
rs1536215	*TRIM27*	0.0037	0.72	0.0806	0.81	0.0627	0.78	0.0684	0.78
rs362521	*OR2H2*	0.0021	0.62	0.0065	0.64	0.0081	0.61	0.0060	0.60
rs3828903	*MICB*	3.20×10^−7^	0.67	0.0024	0.78	0.0759	0.86	0.0965	0.86
rs2246626	*MICB*	3.45×10^−4^	0.77	0.2570	0.91	0.7664	1.04	0.6680	1.04
rs8283	*CREBL1*	7.67×10^−7^	0.63	0.0013	0.74	0.0117	0.78	0.0111	0.78
rs7746019	*C6orf10*	7.85×10^−12^	1.77	0.1075	1.20	0.0244	1.30	0.0224	1.31
rs3117103	*C6orf10*	2.42×10^−16^	2.35	0.3961	1.47	0.3701	1.52	0.3699	1.50
rs7769979	*DQB2*	5.64×10^−14^	0.58	1.90×10^−6^	0.69	0.0013	0.75	0.0030	0.76
rs10947345	*DQB2*	6.96×10^−4^	1.29	1.21×10^−7^	1.53	0.0002	1.42	0.0004	1.40
rs383711	*HSD17B8*	0.0024	0.65	0.0308	0.73	0.0949	0.77	0.1082	0.77

OR = odds ratio.

**†:** Haplotypes assigned in SLE cases and all family members using PHASE v. 2.1.2. Non-transmitted haplotypes were used as control group. Haplotypes containing SLE-associated *HLA-DRB1* alleles were sequentially removed (see Methods).

**Table 4:** Results from conditional haplotype method analyses of top MHC SNPs in SLE cases and controls.

		DRB1*0301 only		DRB1*1501 only		DRB1*1401 only	
SNP	Gene	P-value	OR	P-value	OR	P-value	OR
rs1536215	*TRIM27*	0.16	0.48	0.7701	0.91	1.000	0.89
rs362521	*OR2H2*	0.43	0.68	0.4213	0.72	NA	NA
rs3828903	*MICB*	0.0059	0.46	0.1216	0.61	0.3422	0.47
rs2246626	*MICB*	0.0615	0.59	0.4478	0.80	0.3614	0.48
rs8283	*CREBL1*	0.0122	0.32	1.0000	1.02	0.2220	NA
rs7746019	*C6orf10*	0.0189	1.94	0.6853	2.05	0.7403	1.40
rs3117103	*C6orf10*	0.0189	1.94	1.0000	1.36	NA	NA
rs7769979	*DQB2*	0.0544	0.54	0.2374	0.70	0.2451	0.30
rs10947345	*DQB2*	0.8908	1.06	0.2536	1.37	0.2451	3.35
rs383711	*HSD17B8*	0.2428	0.43	0.2013	0.59	0.5988	0.43

OR = odds ratio.

**†:** Haplotypes assigned in SLE cases and all family members using PHASE v. 2.1.2. Non-transmitted haplotypes were used as control group.

In summary, we have characterized a large set of SLE cases and parents of European ancestry for the highly polymorphic *HLA-DRB1* locus and ∼2,000 SNPs across 4.9 Mb of the MHC. We have employed methods to ensure that our main results are not due to confounding by population admixture or major substructure within the European population. Our results support the existence of multiple, independent association signals across this region as opposed to a single primary association signal (with other effects explained by LD). Independent associations within extended class I and both class II and III regions were present, even after accounting for *HLA-DRB1* effects. However, we cannot exclude the possibility that multiple associated variants within MHC haplotypes function together to influence SLE risk through co-regulation or other mechanisms.

An important limitation of the current analysis is the lack of comprehensive coverage in the MHC region. For example, the MHC Panel Set of markers was not designed to directly assess rare variants or multi-allelic markers, such as insertion-deletion or copy number variants. In addition, we have not characterized these families for other classical HLA genes (besides *HLA-DRB1*), and in fact our results implicate a role for other HLA class II region genes. Thus, we cannot determine which, if any, of the associated variants we have identified represent underlying causal variants, versus proxies for causal variants as a result of LD. In order to begin to address this important question we identified SNPs and other variants tagged by the independently associated SNPs and summarized their known function, as well as what is known about these variants themselves (see Table S7).

One of the novel loci highlighted in the current study is *OR2H2*, within the extended class I region, which encodes olfactory receptor family 2, subfamily H, number 2. The olfactory receptor proteins are G-protein-coupled receptors that share structure with many neurotransmitter and hormone receptors [24]. To our knowledge, this gene has not been implicated in risk of SLE or other human autoimmune diseases. Our top SNP in this region tags three additional SNPs in a single locus, gamma-aminobutyric acid (GABA) B receptor, 1 (*GABBR1*, see Table S7), which is also a neurotransmitter receptor [25]. Of relevance to this finding, recent chromosome 6 sequencing efforts have now fully characterized the extended MHC in humans [26], spanning a total of 7.6 Mb and comprised of five subregions that include the original ‘classical’ MHC [27]. Of the 421 loci in this region, a total of 252 (60%) are classified as expressed genes. Although our study included 305 SNPs within the extended class I MHC and 247 within the extended class II MHC, more work is needed to interrogate the entire extended MHC region.

Our top SNP within the class III region was located in the 3′ untranslated region of *CREBL1*, which encodes c-AMP responsive element binding protein-like 1 [28]. Of interest, we did not identify additional variants tagged by our top SNP in this region (see Table S7). Unfortunately, our MHC marker set did not include SNPs within or near the *C4* locus, which has been previously associated with SLE. In particular, copy number variation at this locus has been strongly implicated in SLE risk. However, the two markers flanking the *C4* locus did not reveal evidence for association with SLE in the current study (rs389512 and rs1009382, p>0.15). Given the density of genes within the class III region and previous work suggesting a contribution of class III variants to SLE risk, further study of this region is clearly warranted, including direct assessment of *C4* variation.

We also demonstrated association of SLE with a variant near the 5′ end of the MHC class I chain-related B (*MICB*) gene, which encodes a heavily glycosylated protein that serves as a ligand for the *NKG2D* type 2 receptor [29]. Binding of this ligand activates the cytolytic response of natural killer cells. This finding is also of interest given previous evidence of association of the closely related *MICA* gene to SLE [30], although results have been contradictory [31]. The *MICB* gene has not been previously implicated in SLE risk. All other variants tagged by our top SNP in this region are within or very near the *MICB* locus (see Table S7).

Lastly, two HLA class II region SNPs at the *DQB2* locus remained associated with SLE even in the absence of *DRB1* risk alleles, and these two SNPs tag variants of at least four additional class II loci (see Table S7). However, our study lacked power to detect additional rare *DRB1* associations (beyond *0301, *1501 and *1401), thus it is possible that additional *DRB1* effects explain, at least in part, the *DQB2* association observed in the current study.

We also sought to determine how our results compare to those reported by Fernando, et al., based on their analysis of 68 SNPs in the HLA class II and III regions, plus *HLA-DRB1*, in 314 UK SLE families [16]. Two distinct association signals centered on DRB1*0301 and the T allele of rs419788 in intron 6 of the class III region gene *SKIV2L* were observed. Five SNPs within this gene were included in our marker panel, including their top SNP, rs419788. Four of the five SNPs were associated with SLE with p<0.01 in our single marker analyses, however, only two of these SNPs (but not rs419788) met the significance threshold (p<0.01) for our CHM analyses. Neither of these two SNPs were among the group of ten SNPs selected for further study based on conditional haplotype based analyses or stepwise logistic regression.

We have not examined other, non-European populations in this study and this represents a very important focus for future work given the increased burden of SLE among those groups. Further, comparison of MHC association results across major ethnic groups, where LD patterns are substantially different, or the analysis of specific disease subtypes may improve our ability to localize causal variants within this region.

## Methods

### Ethics statement

Written consent was obtained from all study participants and ethical approval for this study was obtained from the University of California, San Francisco Committee on Human Research.

### Subjects

SLE trio families and cases were derived from three independent case series, including the UCSF Lupus Genetics Project collection, the Lupus Genetics Studies and Lupus Family Registry and Repository (http://lupus.omrf.org/) at the Oklahoma Medical Research Foundation (OMRF) and the University of Minnesota SLE collection. SLE cases from trio families (n = 735) were a subset of the total set of SLE cases (n = 1,610), as shown in Table 1. Details of recruitment and enrollment procedures have been reported previously [32],[33],[34]. All subjects were self-reported Caucasian and all SLE cases met the American College of Rheumatology classification criteria for SLE [35],[36]. Non-transmitted *HLA-DRB1* and MHC SNP alleles (described below) from SLE trio family parents were used as ‘controls’ for all comparisons with SLE cases, as previously described [19].

### DNA samples

DNA was extracted from whole blood using the Gentra Puregene Whole Blood Kit. DNA was obtained from buccal cells and extracted using the Gentra Puregene Mouse Tail Kit. A total of 50 ng of buccal cell or 25 ng of blood DNA (when necessary) was whole genome amplified (WGA) using the Sigma WGA2 Kit prior to genotyping. WGA samples were column purified using the Sigma Genelute PCR Clean-up Kit. DNA was also extracted from DNA Genotek Oragene saliva sample collection kits according to the manufacturer's protocol. Of the total 3,080 individual DNA samples used for genotyping in this study, 69% were derived from whole blood, 25% from buccal cells, and 6% from saliva; 72% of all samples (including both blood and buccal) were WGA prior to genotyping.

### Genotyping methods

#### HLA-DRB1

Genotyping for the classical *HLA-DRB1* locus was performed using a PCR-Sequence-Specific Oligonucleotide Probe (SSOP) methodology [37], also known as “linear array” technology. Briefly, this technology employs biotin-labeled amplification products, generated from the polymorphic exons of the HLA genes, in a hybridization reaction with a series of unlabelled oligonucleotides. These oligonucleotides, which correspond to variable sequence motifs in the genes, are immobilized onto a nylon backed membrane. Hybridization is visualized colorimetrically, and signal intensity is automatically quantitated with a flatbed scanner and in-house software. Genotype calling was achieved using SCORE, HLA genotyping software developed by Dr. Wolfgang Helmberg (www.ncbi.gov). The DRB1 HiRes assay specifically amplifies only *DRB1* alleles, thus removing the confounding signals from other *DRB* loci that can be found in commercial assays. A total of 100–200 ng was used, depending on DNA source.

#### MHC SNP genotyping

All DNA samples were genotyped for MHC region SNPs using the Combined MHC genotyping panel (2,360 SNPs) from Illumina. This panel spans 4.9 Mb across the MHC region with ∼2 kb/SNP spacing and utilizes the robust Golden-Gate technology [38]. The Combined MHC Panel Set is comprised of both the Exon-Centric and Mapping panels. The Exon-Centric Panel consists of 1,228 SNPs which are located in or near coding regions (within 10 kb of exons). The Mapping Panel consists of 1,293 SNPs. Genotyping was performed in a 96-well format utilizing the 1,536 Sentrix Array Matrix (×2). A total of 250 ng to 1 ug was used for each Illumina assay, depending on source of DNA.

#### Ancestry informative markers (AIMs)

A total of 384 SNP markers selected for both Continental and European ancestry informativeness, including differentiation between Northern vs. Southern European origin were genotyped for all SLE cases [39],[40] (see Table S4). Genotypes were derived using a custom Illumina Golden Gate 384 SNP panel (∼65% of SLE cases) or the Illumina HumHap 550 BeadChip (∼35% of cases). A total of 22 samples were genotyped on both platforms for comparison (see QC methods below)

#### Quality control analysis for DNA samples and genotypes

Stringent quality control criteria were applied to all samples and genotype data (*HLA-DRB1*, MHC region SNPs, and AIMs). Sample success rate for *HLA-DRB1* was 97.9%. Mendelian inconsistencies for *DRB1* were identified in five SLE trio families. These individuals (n = 15) were excluded from further analyses. A per-sample genotype rate threshold of 80% was used for MHC SNP genotyping (total n = 173 samples removed); a per-SNP genotype rate threshold of 90% was used (total n = 337 SNPs removed). Additional criteria including assessment of SNP minor allele frequency (<0.01), Hardy-Weinberg equilibrium (p<0.001), and both Mendelian (>0.01) and gender inconsistencies identified six additional families (n = 18 individuals) and 49 additional SNPs for exclusion from analyses. Analysis of replicate SNPs (n = 146 SNPs occurring on both Illumina Mapping and Exoncentric panels) demonstrated very strong evidence for agreement; 99.7% of all genotypes for all samples compared were the same. Replicate sample comparisons within and across DNA genotyping plates, as well as between different sample sources (blood vs. saliva or blood vs. buccal) also demonstrated very strong evidence for agreement (data not shown). A total of 2,873 samples (665 trios and 878 SLE cases without parents) and 1,974 MHC SNPs passed our stringent quality control criteria. The mean distance between MHC SNPs was 2.08 kb (range: 0.005–71.05 kb). The total MHC SNP coverage spanned 4,913.28 kb. Similar quality control criteria were used for all AIMs genotype data. A total of 27 SNPs were removed from analysis, leaving a final dataset comprised of genotypes from 357 SNPs (Table S4). A total of 22 samples were genotyped using both platforms for comparison demonstrating very high agreement (data not shown). Five additional SLE cases and three additional trio families were removed from further analyses due to missing AIM data. Results of AIM genotyping and analysis among all SLE cases who reported Caucasian ancestry revealed 51 SLE cases with an estimated European ancestral contribution <80% and these individuals (plus their parents, total n = 75) were excluded from further analysis. Two datasets were created for analysis of *HLA-DRB1* and MHC SNP loci based on European ancestry: >80% European ancestry (650 trio families and 834 SLE cases; total n = 2,784 individuals); >90% European ancestry (614 trio families and 765 SLE cases; total n = 2,607 individuals). The first of these datasets (>80% European ancestry) was used for initial univariate and conditional haplotype analyses (see below). The second dataset (>90% European ancestry) was used for subsequent analyses. A third dataset comprised of an even more genetically homogeneous subgroup of cases (n = 1,379) who were estimated based on AIMs to have at least 90% Northern (vs. Southern) European ancestry was also used to examine the final MHC SNP variants identified in the study.

#### Analysis of *HLA-DRB1*

Global testing for heterogeneity at the *DRB1* locus was performed using SAS v. 9.1.3 (Cary, NC). A method to reveal the relative predispositional effects (RPEs) (predisposing, protective, or neutral) of the *HLA-DRB1* alleles was also used, as previously described [20],[41]. When a disease is associated with two or more alleles at a locus, the RPE method identifies the associations sequentially according to their strength; thus the problem that a strong association with one allele can create misleading deviations in the frequencies of other alleles is alleviated.

#### Univariate analysis of MHC variants

Univariate analysis of MHC region SNPs was performed using the log likelihood ratio test implemented in the UNPHASED program v.3.0.10 [42]. Transmission disequilibrium testing (TDT) [21] analyses of individual markers were performed using the PLINK program [22].

#### Conditional methods of analysis

The conditional haplotype method (CHM) [43],[44], was used to initially distinguish primary from secondary MHC associations. The CHM is a test for homogeneity of relative allele frequencies in cases and controls (or transmitted and non-transmitted alleles in families) at a test locus on haplotypes identical for alleles at another locus. Specifically, the CHM tests the null hypothesis of equality in the distribution of transmitted (T) and non-transmitted (NT) marker haplotypes identical at one variant but different at another closely linked variant. If there is heterogeneity between the T and NT (case-control) groups in the distribution of two marker haplotypes identical at a predisposing marker (variant A) but different at a putative predisposing marker at another site (variant B), then this is evidence that variant A does not entirely explain disease predisposition and that variant B itself, or another marker in LD with variant B, is influencing the relative association of variant A, and therefore disease risk. We performed initial analyses of all MHC SNPs in the SLE cases and non-transmitted controls, conditioning on the top SNP and each *DRB1* susceptibility allele. SNPs associated with SLE at p<0.01 based on initial univariate analyses were included in these analyses (n = 544 SNPs; see Figure 2). All haplotype counts were determined using UNPHASED v.3.0.10 [42]. In addition, the CHM was used for analysis of the 11 final MHC variants. Here, full haplotypes including *HLA-DRB1* were assigned using PHASE v.2.1.1, as described below. The Fisher's exact test in SAS was used to calculate OR, 95% CI and p-values for each analysis.

We also used the WHAP program [45] version 2.09 (http://pngu.mgh.harvard.edu/~purcell/whap/) to further evaluate the variants identified using the CHM to have some evidence of independent association with SLE susceptibility (p<0.01; see above and Figure 1). A per-block WHAP method was utilized. More specifically, within each haplotype block, we performed a conditional analysis starting with *HLA-DRB1*, which was modeled as a multi-allelic marker with values DRB1*0301, DRB1*1401, DRB1*1501, or other. Starting with *DRB1*, we tested each candidate marker for an independent effect; in particular this uses a likelihood-ratio test to determine the significance (conditional p-value) of the difference between an “alternate” model with 2-locus haplotypes - composed of *DRB1* plus each new candidate - versus the initial 1-marker (*DRB1* only) “null” model. As long as the most significant locus out of the list of candidates has conditional p<0.01, we add this best marker to the list of independent markers (now *DRB1* plus one SNP), and proceed to test all remaining SNPs as part of 3-marker haplotypes - again composed of the current list plus each new candidate marker - compared to our 2-locus model; and so on with larger haplotypes as appropriate. The algorithm completes when no remaining candidate SNP has p<0.01 conditional on the current list of significant markers. Haplotype block structure for WHAP analysis was defined using solid spline (http://www.biostat.wustl.edu/genetics/geneticssoft/manuals/haploview/haploview_doc.pdf).

The Tagger program [46] was used to select MHC SNPs identified from CHM analyses (p<0.01 as described above) for further analysis using stepwise logistic regression modeling, and also for retrieving tagged SNPs from CEPH Hap Map data as previously described (see below). Stepwise logistic regression analyses were performed using STATA v.9.2 (College Station, Texas). Forward stepwise was performed using thresholds of p<0.01 to add a variable and p≥0.0101 to remove a variable from our model. For the stepwise logistic regression analyses, MHC SNPs were coded for presence/absence of the minor allele. *HLA-DRB1* was coded according to genotype for SLE associated alleles (*0301*, *1501*, and *1401*), with all other alleles as the reference group. Logistic regression analysis including the final top MHC SNPs and *HLA-DRB1* was performed using a similar approach.

#### Imputation of missing MHC SNP data

Missing genotypes for cases and non-transmitted controls were inferred using default parameters in BEAGLE v2.1.3 [47]. The entire dataset served as the reference group. Briefly, four haplotype pairs were sampled per individual for each iteration of the phasing algorithm. Ten iterations of the phasing algorithm were performed. The threshold scale and threshold shift, which are used to control the number of haplotype clusters in the graphical model for the phased data, were set to 4.0 and 0.2, respectively. The maximum number of consecutive markers considered for determining whether to merge haplotype clusters was 500. The number of missing genotypes imputed in this study was small, at 1.47% of all study genotypes (across 1,974 SNPs and 2,784 individuals, or ∼5.5 million genotypes). We used the resulting phased data file, which gives the most likely genotypes for each individual, as input for stepwise logistic regression analyses of cases and controls described above.

#### Linkage disequilibrium analyses

Haploview (v.4.1) [48] was used to define LD among the final group of MHC SNP variants using all cases and founders from SLE families. Similarly, for the multi-allelic *HLA-DRB1* locus, all pairwise measures of LD with MHC region SNPs were determined using UNPHASED (-LD option) [42]. Due to the restriction of Haploview to analyze and display measures of LD for biallelic markers, *HLA-DRB1* was recoded. We chose DRB1*0301 vs. other alleles for this analysis based on results obtained for LD analyses of this locus as a multilocus marker using UNPHASED.

#### Haplotype assignment and analysis

PHASE v.2.1.1 was used to assign extended multilocus haplotypes in all SLE cases and parents comprised of *HLA-DRB1* and our top 10 MHC SNPs. Default settings for number of iterations (n = 100), thinning interval (n = 1) and burn-in (n = 100) were used. RPE analysis and tests of heterogeneity to compare MHC SNP effects on *HLA-DRB1* haplotypes were performed using SAS. OR, 95% CI and p-values are reported.

#### Identification of tagged variants

LD data calculated by HapMap for the CEPH population were downloaded directly from the HapMap Genome Browser (http://hapmap.org/cgi-perl/gbrowse) for phase I and II SNPs (release #23a, NCBI Build 36, June 2008) and phase III SNPs (draft release #1, Sept 2008). For each study SNP, LD was assessed for all SNPs within 500 kb flanking each side of the SNP. In addition, for two of our study SNPs not present in NCBI Build 36, CEPH genotypes were downloaded directly from HapMap and LD between each of these SNPs and other SNPs within 500 kb in NCBI Build 35 was assessed using the Tagger algorithm as implemented in Haploview (v 4.1) by force including each SNP and force excluding all others. For each release and method, SNPs typed in the CEPH were considered tagged by our study SNPs if they showed r^2^≥0.80. SNPs shown to be tagged, but already typed and assessed in our study were not included as tagged variants. For study SNPs not typed in both phases, LD between these SNPs and SNPs typed only in the alternate phase could not be assessed. For study SNPs not included in NCBI Build 36, LD could only be assessed between these and SNPs typed in phase I and phase II that were also present in NCBI Build 35. Annotation for each study SNP and tagged SNP including nearest gene, SNP function, and heterozygosity was taken from the UCSC Genome Browser (http://genome.ucsc.edu/).

## Supporting Information

Table S1 Relative predispositional effects (RPE) analysis of *HLA-DRB1* alleles among 1,522 SLE cases versus 693 controls.
(0.09 MB DOC)

Table S2 Association results for 1,974 SNPs among 1,484 SLE cases and 650 controls.
(3.70 MB DOC)

Table S3 List of MHC SNPs (n = 171) associated with SLE with p<0.01 based on conditional haplotype method among 1,484 SLE cases and 650 controls.
(0.16 MB DOC)

Table S4 Ancestry informative markers (total n = 357) genotyped to classify SLE cases (n = 1,570) according to continental ancestry (n = 112 SNPs) and European substructure (n = 246 SNPs).
(0.39 MB DOC)

Table S5 Results from analyses of independently associated MHC variants in SLE families and Northern European individuals (see Figure 3 and Figure 4).
(0.04 MB DOC)

Table S6 Results from relative predispositional effects (RPE) (A) and CHM analyses (B) of top MHC SNPs in SLE cases and controls. (Same as Table 3 and Table 4 with 95% confidence intervals included.)
(0.08 MB DOC)

Table S7 Details for top associated study MHC SNPs plus variants tagged by these SNPs (see Figure 3, Figure 4, Table 3, Table 4).
(0.15 MB DOC)
