# Supplementary material for: High-Density SNP Screening of the Major Histocompatibility Complex in Systemic Lupus Erythematosus Demonstrates Strong Evidence for Independent Susceptibility Regions
Source: PLoS Genet. 2009 Oct 23;5(10):e1000696. doi: 10.1371/journal.pgen.1000696 (PMC2758598; doi:10.1371/journal.pgen.1000696)
Supplement: Table S1 — Relative predispositional effects (RPE) analysis of HLA-DRB1 alleles among 1,522 SLE cases versus 693 controls. (0.09 MB DOC) [file pgen.1000696.s001.doc]

**Table S1.** Relative predispositional effects (RPE) analysis of *HLA-DRB1* alleles among 1,522 SLE cases versus 693 controls*.

| **DRB1 Allele** | **Case alleles (frequency)** | **Control alleles (frequency)** | **Chi square (1)** | **P-value** | **OR (95% CI)** | **Chi square (2)** | **P-value** | **Chi square (3)** | **P-value** | **Chi square (4)** | **P-value** |
| --- | --- | --- | --- | --- | --- | --- | --- | --- | --- | --- | --- |
| 101 | 227(0.075) | 125 (0.090) | 2.90 | 0.0886 | 0.81 (0.65, 1.02) | 0.45 | 0.5023 | 0.75 | 0.3860 | 0.12 | 0.7290 |
| 102 | 29 (0.010) | 17 (0.012) | 0.68 | 0.4096 | 0.77 (0.42, 1.41) | 0.20 | 0.6547 | 0.27 | 0.6021 | 0.11 | 0.7401 |
| 103 | 31 (0.010) | 18 (0.013) | 0.67 | 0.4131 | 0.78 (0.44, 1.40) | 0.19 | 0.6629 | 0.26 | 0.6135 | 0.10 | 0.7518 |
| 301 | 625 (0.206) | 153 (0.110) | 49.02 | 2.53e-12 | 2.08 (1.72, 2.52) | -- | -- | -- | -- | -- | -- |
| 401 | 244 (0.080) | 112 (0.081) | 0.00 | 1.0000 | 0.99 (0.79, 1.25) | 0.89 | 0.3455 | 0.56 | 0.4535 | 1.59 | -- |
| 402 | 37 (0.012) | 11 (0.008) | 1.57 | 0.2102 | 1.54 (0.78, 3.02) | 2.55 | 0.1103 | 2.34 | 0.1258 | 2.91 | 0.0880 |
| 403 | 26 (0.009) | 7 (0.005) | 1.56 | 0.2117 | 1.70 (0.73, 3.92) | 2.35 | 0.1253 | 2.18 | 0.1397 | 2.63 | 0.1049 |
| 404 | 100 (0.033) | 58 (0.042) | 2.14 | 0.1435 | 0.78 (0.56, 1.08) | 0.59 | 0.4424 | 0.81 | 0.3681 | 0.30 | 0.5839 |
| 405 | 11 (0.004) | 6 (0.004) | 0.13 | 0.7184 | 0.83 (0.31, 2.26) | 0.02 | 0.8875 | 0.03 | 0.8639 | 0.00 | 1.0000 |
| 407 | 25 (0.008) | 17 (0.012) | 1.64 | 0.2003 | 0.67 (0.36, 1.24) | 0.83 | 0.3623 | 0.96 | 0.3261 | 0.63 | 0.4274 |
| 408 | 7 (0.002) | 9 (0.006) | 4.63 | 0.0314 | 0.35 (0.13, 0.95) | 3.59 | 0.0581 | 3.78 | 0.0518 | 3.30 | 0.0693 |
| 701 | 347 (0.114) | 179 (0.129) | 1.82 | 0.1773 | 0.87 (0.72, 1.05) | 0.01 | 0.9203 | 2.86 | 0.0907 | 0.09 | 0.7642 |
| 801 | 94 (0.031) | 32 (0.023) | 2.05 | 0.1522 | 1.35 (0.90, 2.02) | 4.00 | 0.0455 | 0.12 | 0.7345 | 4.75 | 0.0293 |
| 901 | 33 (0.011) | 14 (0.010) | 0.05 | 0.8231 | 1.07 (0.57, 2.01) | 0.34 | 0.5598 | 3.57 | 0.0588 | 0.49 | 0.4839 |
| 1001 | 16 (0.005) | 15 (0.011) | 4.21 | 0.0402 | 0.48 (0.24, 0.98) | 2.94 | 0.0864 | 0.27 | 0.6060 | 2.59 | 0.1075 |
| 1101 | 113 (0.037) | 79 (0.057) | 8.64 | 0.0033 | 0.64 (0.48, 0.86) | 4.60 | 0.0320 | 3.16 | 0.0754 | 3.59 | 0.0581 |
| 1102 | 6 (0.002) | 5 (0.004) | 1.02 | 0.3125 | 0.55 (0.17, 1.79) | 0.66 | 0.4166 | 5.27 | 0.0217 | 0.57 | 0.4503 |
| 1103 | 27 (0.009) | 17 (0.012) | 1.10 | 0.2943 | 0.72 (0.39, 1.33) | 0.46 | 0.4976 | 0.73 | 0.3938 | 0.31 | 0.5777 |
| 1104 | 71 (0.023) | 40 (0.029) | 1.16 | 0.2815 | 0.80 (0.54, 1.19) | 0.24 | 0.6242 | 0.56 | 0.4556 | 0.10 | 0.7518 |
| 1201 | 32 (0.011) | 22 (0.016) | 2.24 | 0.1345 | 0.66 (0.38, 1.14) | 1.15 | 0.2835 | 0.36 | 0.5459 | 0.89 | 0.3455 |
| 1301 | 131 (0.043) | 83 (0.060) | 5.57 | 0.0183 | 0.71 (0.53, 0.94) | 2.35 | 0.1253 | 1.33 | 0.2482 | 1.62 | 0.2031 |
| 1302 | 146 (0.048) | 58 (0.042) | 0.78 | 0.3771 | 1.15 (0.85, 1.57) | 2.65 | 0.1035 | 2.21 | 0.1375 | 3.47 | 0.0625 |
| 1303 | 22 (0.007) | 15 (0.011) | 1.47 | 0.2253 | 0.67 (0.34, 1.29) | 0.75 | 0.3865 | 0.86 | 0.3524 | 0.57 | 0.4503 |
| 1401 | 45 (0.015) | 49 (0.035) | 18.95 | 1.34e-5 | 0.41 (0.27, 0.62) | 14.07 | 0.0002 | -- | -- | -- | -- |
| 1501 | 500 (0.165) | 195 (0.141) | 3.41 | 0.0648 | 1.20 (1.00, 1.44) | 10.33 | 0.0013 | 8.71 | 0.0032 | -- | -- |
| 1502 | 16 (0.005) | 14 (0.010) | 3.29 | 0.0697 | 0.52 (0.25, 1.06) | 2.21 | 0.1371 | 2.40 | 0.1213 | 1.92 | 0.1659 |
| 1601 | 43 (0.014) | 19 (0.014) | 0.01 | 0.9203 | 1.03 (0.60, 1.78) | 0.28 | 0.5967 | 0.20 | 0.6534 | 0.44 | 0.5071 |

*Number of individuals with *HLA-DRB1* genotypes passing quality control (see Methods).

(1) Test of heterogeneity with all alleles >2% included. Global Chi square = 120.70 (df=26), p-value = 9.99 x 10^-16^

1. Test of heterogeneity with *DRB1*0301* removed. Global Chi square = 58.68, p-value = 0.0002
2. Test of heterogeneity with *DRB1*0301* and **1401* removed. Global Chi square = 44.56, p-value = 0.0066
3. Test of heterogeneity with *DRB1*0301, *1401*, and **1501* removed. Global Chi square = 33.08, p-value = 0.0798
