# Supplementary material for: High-Density SNP Screening of the Major Histocompatibility Complex in Systemic Lupus Erythematosus Demonstrates Strong Evidence for Independent Susceptibility Regions
Source: PLoS Genet. 2009 Oct 23;5(10):e1000696. doi: 10.1371/journal.pgen.1000696 (PMC2758598; doi:10.1371/journal.pgen.1000696)
Supplement: Table S2 — Association results for 1,974 SNPs among 1,484 SLE cases and 650 controls. (3.70 MB DOC) [file pgen.1000696.s002.doc]

**Table S2.** Association results for 1,974 SNPs among 1,484 SLE cases and

650 controls. * MAF = minor allele frequency from CEPH families and unrelated founders from the 650 SLE trio families. † Odds ratios calculated using major allele as reference. ‡ p-value from UNPHASED v3.0.10.

| **SNP** | **Position, bp** | **CEPH MAF*** | **MAF in 1,300 founders** | **Odds ratio^†^** | **95% CI** | **p-value^‡^** |
| --- | --- | --- | --- | --- | --- | --- |
| rs381808 | 28970148 | 0.50 | 0.49 | 1.00 | (0.90, 1.10) | 0.9378 |
| rs209130 | 28975779 | 0.49 | 0.50 | 0.97 | (0.88, 1.07) | 0.5107 |
| rs1536215 | 28975894 | 0.14 | 0.11 | 0.77 | (0.66, 0.90) | 0.0009 |
| rs209122 | 28983481 | 0.50 | 0.49 | 0.98 | (0.89, 1.08) | 0.6581 |
| rs2269553 | 28984488 | 0.20 | 0.21 | 1.04 | (0.92, 1.17) | 0.5185 |
| rs3135293 | 28985226 | 0.11 | 0.11 | 1.37 | (1.18, 1.60) | 4.77E-05 |
| rs388017 | 28988595 | 0.02 | 0.03 | 1.25 | (0.90, 1.72) | 0.1785 |
| rs929042 | 28998779 | 0.20 | 0.21 | 0.99 | (0.87, 1.13) | 0.9163 |
| rs2765229 | 29000898 | 0.49 | 0.50 | 0.98 | (0.89, 1.08) | 0.6914 |
| rs2894066 | 29001906 | 0.36 | 0.40 | 1.12 | (1.01, 1.23) | 0.0281 |
| rs3763338 | 29002290 | 0.12 | 0.09 | 0.80 | (0.68, 0.96) | 0.0144 |
| rs1237485 | 29002323 | 0.50 | 0.49 | 0.97 | (0.88, 1.07) | 0.5414 |
| rs3118361 | 29006266 | 0.11 | 0.11 | 1.37 | (1.17, 1.59) | 7.65E-05 |
| rs2032502 | 29009544 | 0.12 | 0.08 | 0.81 | (0.68, 0.98) | 0.0263 |
| rs3135329 | 29013083 | 0.36 | 0.39 | 1.10 | (1.00, 1.22) | 0.0526 |
| rs3130843 | 29016193 | 0.38 | 0.40 | 1.12 | (1.01, 1.24) | 0.0259 |
| rs2071788 | 29020286 | 0.12 | 0.09 | 0.78 | (0.66, 0.93) | 0.0062 |
| rs763009 | 29023087 | 0.50 | 0.48 | 0.97 | (0.88, 1.07) | 0.5257 |
| rs6456876 | 29026915 | 0.39 | 0.40 | 0.91 | (0.83, 1.01) | 0.0624 |
| rs4947256 | 29029919 | 0.12 | 0.08 | 0.82 | (0.68, 0.98) | 0.0320 |
| rs9380100 | 29031959 | 0.12 | 0.08 | 0.80 | (0.67, 0.96) | 0.0167 |
| rs12386522 | 29036146 | 0.05 | 0.05 | 0.79 | (0.62, 1.01) | 0.0564 |
| rs9257425 | 29044535 | 0.25 | 0.27 | 1.00 | (0.89, 1.11) | 0.9351 |
| rs6933672 | 29049667 | 0.09 | 0.10 | 0.93 | (0.78, 1.10) | 0.3708 |
| rs3135322 | 29054899 | 0.50 | 0.49 | 0.97 | (0.88, 1.07) | 0.5201 |
| rs6904975 | 29060014 | 0.38 | 0.39 | 0.90 | (0.82, 1.00) | 0.0439 |
| rs3129791 | 29062272 | 0.11 | 0.11 | 1.38 | (1.18, 1.61) | 3.56E-05 |
| rs10946966 | 29065562 | 0.11 | 0.10 | 0.84 | (0.72, 0.99) | 0.0361 |
| rs7776164 | 29066437 | 0.07 | 0.08 | 1.08 | (0.90, 1.29) | 0.3880 |
| rs6456879 | 29070583 | 0.17 | 0.16 | 0.81 | (0.71, 0.93) | 0.0022 |
| rs2269555 | 29072826 | 0.40 | 0.41 | 0.91 | (0.82, 1.00) | 0.0510 |
| rs9468459 | 29073670 | 0.05 | 0.04 | 0.84 | (0.65, 1.09) | 0.1793 |
| rs6920392 | 29075017 | 0.39 | 0.40 | 0.91 | (0.82, 1.00) | 0.0551 |
| rs6906909 | 29076125 | 0.39 | 0.40 | 0.91 | (0.83, 1.01) | 0.0742 |
| rs9257453 | 29076909 | 0.07 | 0.08 | 1.07 | (0.89, 1.28) | 0.4684 |
| rs6925178 | 29081194 | 0.23 | 0.24 | 0.86 | (0.76, 0.96) | 0.0088 |
| rs9257461 | 29085904 | 0.39 | 0.40 | 0.92 | (0.83, 1.02) | 0.0957 |
| rs6930903 | 29089223 | 0.23 | 0.24 | 0.87 | (0.78, 0.98) | 0.0168 |
| rs9468464 | 29092491 | 0.05 | 0.05 | 0.80 | (0.64, 1.01) | 0.0582 |
| rs9380103 | 29093184 | 0.12 | 0.09 | 0.78 | (0.65, 0.93) | 0.0053 |
| rs6941946 | 29096562 | 0.38 | 0.40 | 0.92 | (0.83, 1.01) | 0.0796 |
| rs2394513 | 29100487 | 0.06 | 0.07 | 1.05 | (0.87, 1.26) | 0.6470 |
| rs10484544 | 29103698 | 0.14 | 0.16 | 1.04 | (0.91, 1.19) | 0.5959 |
| rs4713197 | 29105564 | 0.12 | 0.11 | 0.85 | (0.72, 0.99) | 0.0349 |
| rs7755402 | 29110041 | 0.14 | 0.16 | 1.02 | (0.89, 1.16) | 0.7874 |
| rs9468473 | 29114229 | 0.23 | 0.23 | 0.88 | (0.78, 0.99) | 0.0267 |
| rs7749435 | 29115826 | 0.23 | 0.23 | 0.88 | (0.78, 0.98) | 0.0240 |
| rs6919044 | 29117547 | 0.23 | 0.23 | 0.87 | (0.78, 0.98) | 0.0223 |
| rs6456889 | 29118803 | 0.23 | 0.23 | 0.88 | (0.79, 1.00) | 0.0413 |
| rs7341218 | 29120095 | 0.16 | 0.16 | 0.98 | (0.85, 1.12) | 0.7263 |
| rs2143574 | 29122005 | 0.38 | 0.39 | 0.90 | (0.81, 1.00) | 0.0398 |
| rs3130756 | 29125080 | 0.49 | 0.49 | 0.96 | (0.87, 1.06) | 0.3886 |
| rs7747023 | 29133659 | 0.17 | 0.16 | 0.82 | (0.72, 0.94) | 0.0035 |
| rs3117143 | 29139121 | 0.11 | 0.11 | 1.37 | (1.17, 1.59) | 6.38E-05 |
| rs12665818 | 29140163 | 0.14 | 0.15 | 1.01 | (0.88, 1.16) | 0.8510 |
| rs2050231 | 29150368 | 0.38 | 0.40 | 0.93 | (0.84, 1.03) | 0.1727 |
| rs9348821 | 29155182 | 0.17 | 0.16 | 0.83 | (0.72, 0.94) | 0.0049 |
| rs11758255 | 29158693 | 0.23 | 0.23 | 0.88 | (0.78, 0.99) | 0.0394 |
| rs7752270 | 29161977 | 0.06 | 0.07 | 0.96 | (0.80, 1.16) | 0.6923 |
| rs9380109 | 29165950 | 0.38 | 0.40 | 0.91 | (0.83, 1.01) | 0.0766 |
| rs2013972 | 29166976 | 0.38 | 0.40 | 0.91 | (0.82, 1.00) | 0.0590 |
| rs12665108 | 29170173 | 0.14 | 0.15 | 0.99 | (0.86, 1.14) | 0.8880 |
| rs4538737 | 29172751 | 0.16 | 0.16 | 1.01 | (0.88, 1.16) | 0.8735 |
| rs9295790 | 29178407 | 0.23 | 0.23 | 0.88 | (0.78, 0.98) | 0.0249 |
| rs3131091 | 29181073 | 0.49 | 0.50 | 0.95 | (0.87, 1.05) | 0.3457 |
| rs6456908 | 29183051 | 0.23 | 0.23 | 0.87 | (0.78, 0.98) | 0.0181 |
| rs3116838 | 29186130 | 0.14 | 0.16 | 1.00 | (0.87, 1.14) | 0.9840 |
| rs3129106 | 29189140 | 0.19 | 0.14 | 0.91 | (0.79, 1.05) | 0.2203 |
| rs3130766 | 29194808 | 0.49 | 0.50 | 0.96 | (0.87, 1.06) | 0.4386 |
| rs2064162 | 29196794 | 0.38 | 0.40 | 0.92 | (0.84, 1.02) | 0.1182 |
| rs3130773 | 29203887 | 0.11 | 0.10 | 1.35 | (1.15, 1.58) | 0.0002 |
| rs3130778 | 29207558 | 0.38 | 0.40 | 0.92 | (0.83, 1.01) | 0.0914 |
| rs7738990 | 29209100 | 0.06 | 0.07 | 1.05 | (0.86, 1.26) | 0.6513 |
| rs3129119 | 29210840 | 0.28 | 0.27 | 1.05 | (0.94, 1.18) | 0.3625 |
| rs9468487 | 29215031 | 0.05 | 0.06 | 0.87 | (0.70, 1.08) | 0.1929 |
| rs9393945 | 29216270 | 0.12 | 0.09 | 0.79 | (0.66, 0.94) | 0.0090 |
| rs3130720 | 29223727 | 0.44 | 0.45 | 1.07 | (0.97, 1.18) | 0.2055 |
| rs3130724 | 29226509 | 0.23 | 0.22 | 1.06 | (0.94, 1.19) | 0.3619 |
| rs3129126 | 29229610 | 0.49 | 0.50 | 0.96 | (0.87, 1.06) | 0.4614 |
| rs2142906 | 29237962 | 0.44 | 0.46 | 1.07 | (0.97, 1.18) | 0.1525 |
| rs2394520 | 29245199 | 0.22 | 0.23 | 1.03 | (0.92, 1.16) | 0.5635 |
| rs3129151 | 29246294 | 0.23 | 0.22 | 1.06 | (0.94, 1.19) | 0.3392 |
| rs3129152 | 29246756 | 0.23 | 0.22 | 1.07 | (0.95, 1.20) | 0.2622 |
| rs3129154 | 29247765 | 0.44 | 0.46 | 1.08 | (0.97, 1.19) | 0.1491 |
| rs3116856 | 29249828 | 0.44 | 0.46 | 1.07 | (0.97, 1.18) | 0.1824 |
| rs3129159 | 29250033 | 0.44 | 0.46 | 1.07 | (0.97, 1.18) | 0.1982 |
| rs3116820 | 29259650 | 0.44 | 0.45 | 1.05 | (0.96, 1.16) | 0.2942 |
| rs9257593 | 29260773 | 0.43 | 0.44 | 1.06 | (0.96, 1.16) | 0.2661 |
| rs3129171 | 29263728 | 0.24 | 0.23 | 1.07 | (0.96, 1.20) | 0.2408 |
| rs3129173 | 29267608 | 0.11 | 0.11 | 1.34 | (1.15, 1.56) | 0.0002 |
| rs1977074 | 29271813 | 0.49 | 0.49 | 0.98 | (0.89, 1.09) | 0.7543 |
| rs3116830 | 29275554 | 0.11 | 0.11 | 1.36 | (1.17, 1.59) | 7.12E-05 |
| rs3129093 | 29278240 | 0.23 | 0.22 | 1.05 | (0.94, 1.18) | 0.3740 |
| rs2394546 | 29283578 | 0.14 | 0.15 | 1.00 | (0.87, 1.15) | 0.9994 |
| rs1883329 | 29285081 | 0.20 | 0.23 | 1.03 | (0.91, 1.15) | 0.6516 |
| rs3130817 | 29287216 | 0.23 | 0.22 | 1.06 | (0.94, 1.19) | 0.3352 |
| rs2894083 | 29290287 | 0.43 | 0.44 | 1.06 | (0.96, 1.17) | 0.2352 |
| rs3130803 | 29297134 | 0.44 | 0.45 | 1.06 | (0.96, 1.17) | 0.2225 |
| rs714470 | 29299390 | 0.43 | 0.44 | 1.06 | (0.96, 1.17) | 0.2448 |
| rs3130812 | 29301764 | 0.43 | 0.44 | 1.05 | (0.95, 1.16) | 0.3246 |
| rs3130814 | 29304160 | 0.39 | 0.40 | 1.04 | (0.95, 1.15) | 0.3972 |
| rs2207338 | 29307803 | 0.38 | 0.37 | 1.06 | (0.96, 1.18) | 0.2339 |
| rs7742939 | 29313312 | 0.44 | 0.45 | 1.06 | (0.96, 1.17) | 0.2381 |
| rs3129105 | 29321049 | 0.49 | 0.49 | 0.99 | (0.90, 1.09) | 0.8097 |
| rs3117330 | 29333774 | 0.38 | 0.37 | 1.06 | (0.96, 1.17) | 0.2663 |
| rs2024679 | 29335096 | 0.49 | 0.50 | 0.99 | (0.90, 1.09) | 0.8536 |
| rs10484545 | 29342489 | 0.05 | 0.06 | 1.06 | (0.87, 1.28) | 0.5779 |
| rs3130830 | 29344701 | 0.48 | 0.49 | 1.01 | (0.91, 1.11) | 0.8805 |
| rs3117326 | 29348357 | 0.11 | 0.09 | 1.33 | (1.12, 1.58) | 0.0012 |
| rs6456942 | 29359379 | 0.49 | 0.49 | 0.99 | (0.90, 1.09) | 0.8757 |
| rs9468508 | 29361735 | 0.05 | 0.05 | 0.77 | (0.61, 0.98) | 0.0298 |
| rs1884123 | 29364399 | 0.38 | 0.38 | 1.05 | (0.95, 1.16) | 0.3715 |
| rs4446587 | 29367602 | 0.48 | 0.48 | 0.99 | (0.90, 1.09) | 0.7917 |
| rs3117425 | 29368410 | 0.11 | 0.11 | 1.30 | (1.12, 1.51) | 0.0006 |
| rs7383161 | 29372634 | 0.43 | 0.42 | 1.00 | (0.91, 1.11) | 0.9541 |
| rs4594993 | 29378477 | 0.04 | 0.07 | 1.01 | (0.83, 1.21) | 0.9479 |
| rs9257694 | 29382465 | 0.48 | 0.49 | 1.03 | (0.94, 1.14) | 0.5479 |
| rs10456370 | 29388715 | 0.49 | 0.47 | 1.01 | (0.92, 1.11) | 0.8601 |
| rs9393954 | 29390330 | 0.13 | 0.12 | 0.82 | (0.70, 0.96) | 0.0112 |
| rs720831 | 29392497 | 0.39 | 0.37 | 1.02 | (0.92, 1.13) | 0.7324 |
| rs4713209 | 29395268 | 0.13 | 0.12 | 0.82 | (0.70, 0.95) | 0.0082 |
| rs16894722 | 29400009 | 0.04 | 0.03 | 0.89 | (0.67, 1.19) | 0.4247 |
| rs12660111 | 29404127 | 0.10 | 0.15 | 0.98 | (0.85, 1.12) | 0.7562 |
| rs6930435 | 29409201 | 0.24 | 0.24 | 1.15 | (1.03, 1.29) | 0.0151 |
| rs3130805 | 29414435 | 0.16 | 0.15 | 1.17 | (1.02, 1.34) | 0.0219 |
| rs9348827 | 29416372 | 0.13 | 0.11 | 0.82 | (0.70, 0.95) | 0.0107 |
| rs1014258 | 29418660 | 0.24 | 0.24 | 1.14 | (1.01, 1.27) | 0.0287 |
| rs6930033 | 29431884 | 0.10 | 0.15 | 0.98 | (0.85, 1.12) | 0.7472 |
| rs4713210 | 29433412 | 0.13 | 0.11 | 0.80 | (0.68, 0.94) | 0.0058 |
| rs7754402 | 29436813 | 0.37 | 0.34 | 1.02 | (0.91, 1.13) | 0.7797 |
| rs9380120 | 29443516 | 0.13 | 0.11 | 0.81 | (0.69, 0.95) | 0.0083 |
| rs4713211 | 29444033 | 0.32 | 0.34 | 1.10 | (0.99, 1.22) | 0.0737 |
| rs6934993 | 29445479 | 0.23 | 0.25 | 1.08 | (0.96, 1.21) | 0.1957 |
| rs4713213 | 29446941 | 0.46 | 0.47 | 0.97 | (0.88, 1.07) | 0.5865 |
| rs7772982 | 29448986 | 0.18 | 0.18 | 0.91 | (0.80, 1.04) | 0.1516 |
| rs7753474 | 29449287 | 0.18 | 0.18 | 0.90 | (0.80, 1.02) | 0.1114 |
| rs3749971 | 29450754 | 0.12 | 0.11 | 1.32 | (1.13, 1.53) | 0.0003 |
| rs2144425 | 29452918 | 0.48 | 0.48 | 1.01 | (0.91, 1.11) | 0.8967 |
| rs238880 | 29455023 | 0.49 | 0.47 | 1.01 | (0.91, 1.11) | 0.8612 |
| rs1419640 | 29458833 | 0.43 | 0.47 | 1.04 | (0.94, 1.14) | 0.4474 |
| rs238872 | 29459852 | 0.45 | 0.48 | 1.03 | (0.94, 1.14) | 0.4928 |
| rs3117444 | 29460922 | 0.45 | 0.47 | 1.03 | (0.94, 1.14) | 0.5244 |
| rs3129681 | 29462362 | 0.45 | 0.47 | 1.04 | (0.94, 1.14) | 0.4436 |
| rs3094549 | 29463127 | 0.30 | 0.31 | 0.95 | (0.86, 1.06) | 0.3866 |
| rs1419638 | 29463498 | 0.42 | 0.36 | 0.92 | (0.83, 1.02) | 0.1227 |
| rs1419635 | 29463897 | 0.28 | 0.28 | 0.98 | (0.88, 1.09) | 0.6860 |
| rs4711185 | 29464025 | 0.42 | 0.38 | 0.92 | (0.84, 1.02) | 0.1334 |
| rs4452630 | 29466912 | 0.48 | 0.44 | 0.87 | (0.79, 0.96) | 0.0064 |
| rs9257823 | 29469714 | 0.48 | 0.43 | 0.89 | (0.80, 0.98) | 0.0184 |
| rs1362063 | 29470272 | 0.48 | 0.44 | 0.89 | (0.81, 0.98) | 0.0212 |
| rs2073154 | 29472794 | 0.48 | 0.44 | 0.87 | (0.79, 0.96) | 0.0062 |
| rs2073151 | 29472930 | 0.48 | 0.43 | 0.89 | (0.80, 0.98) | 0.0209 |
| rs4713218 | 29473953 | 0.48 | 0.44 | 0.89 | (0.81, 0.98) | 0.0192 |
| rs2158279 | 29474244 | 0.49 | 0.44 | 0.88 | (0.80, 0.97) | 0.0133 |
| rs4711187 | 29475569 | 0.48 | 0.44 | 0.87 | (0.79, 0.96) | 0.0076 |
| rs9405124 | 29476792 | 0.28 | 0.28 | 0.98 | (0.88, 1.10) | 0.7708 |
| rs2394604 | 29477247 | 0.19 | 0.15 | 0.80 | (0.70, 0.93) | 0.0024 |
| rs1419634 | 29477651 | 0.48 | 0.44 | 0.89 | (0.80, 0.98) | 0.0159 |
| rs429479 | 29480302 | 0.12 | 0.11 | 1.38 | (1.19, 1.60) | 2.87E-05 |
| rs994321 | 29480335 | 0.40 | 0.37 | 1.11 | (1.01, 1.23) | 0.0363 |
| rs720497 | 29490412 | 0.40 | 0.37 | 1.12 | (1.01, 1.23) | 0.0359 |
| rs1362073 | 29491299 | 0.29 | 0.30 | 0.97 | (0.87, 1.08) | 0.5690 |
| rs12207410 | 29494585 | 0.28 | 0.28 | 0.98 | (0.88, 1.09) | 0.7355 |
| rs6456947 | 29496285 | 0.31 | 0.32 | 0.92 | (0.83, 1.02) | 0.1010 |
| rs2523421 | 29498419 | 0.13 | 0.08 | 0.98 | (0.83, 1.16) | 0.7942 |
| rs1419643 | 29499915 | 0.40 | 0.37 | 1.11 | (1.01, 1.23) | 0.0375 |
| rs7754054 | 29500482 | 0.29 | 0.30 | 0.97 | (0.87, 1.08) | 0.5810 |
| rs2074470 | 29502406 | 0.31 | 0.32 | 0.94 | (0.84, 1.06) | 0.3159 |
| rs10946990 | 29504625 | 0.28 | 0.28 | 0.98 | (0.88, 1.10) | 0.7900 |
| rs3131025 | 29506794 | 0.31 | 0.31 | 0.91 | (0.82, 1.01) | 0.0782 |
| rs7349863 | 29506838 | 0.28 | 0.28 | 0.98 | (0.87, 1.09) | 0.6785 |
| rs9257858 | 29508215 | 0.22 | 0.20 | 0.85 | (0.75, 0.97) | 0.0146 |
| rs16894908 | 29510414 | 0.02 | 0.03 | 0.83 | (0.61, 1.13) | 0.2337 |
| rs1535039 | 29519411 | 0.12 | 0.11 | 1.37 | (1.18, 1.59) | 4.07E-05 |
| rs7739243 | 29522592 | 0.02 | 0.02 | 1.04 | (0.74, 1.46) | 0.8358 |
| rs2523442 | 29525796 | 0.28 | 0.25 | 0.93 | (0.83, 1.04) | 0.1952 |
| rs3094574 | 29530071 | 0.40 | 0.42 | 1.03 | (0.94, 1.14) | 0.5117 |
| rs9257863 | 29533563 | 0.20 | 0.16 | 0.83 | (0.72, 0.95) | 0.0073 |
| rs3117194 | 29533987 | 0.40 | 0.42 | 1.04 | (0.94, 1.15) | 0.4381 |
| rs2523439 | 29535499 | 0.28 | 0.25 | 0.89 | (0.79, 1.01) | 0.0640 |
| rs2021729 | 29537371 | 0.28 | 0.26 | 0.91 | (0.81, 1.01) | 0.0816 |
| rs3128854 | 29539677 | 0.40 | 0.42 | 1.03 | (0.93, 1.14) | 0.5612 |
| rs1345228 | 29540369 | 0.19 | 0.16 | 0.82 | (0.71, 0.93) | 0.0031 |
| rs2107192 | 29541921 | 0.28 | 0.25 | 0.92 | (0.82, 1.03) | 0.1494 |
| rs2107191 | 29542274 | 0.40 | 0.42 | 1.04 | (0.94, 1.14) | 0.4521 |
| rs4713226 | 29542393 | 0.13 | 0.10 | 0.87 | (0.74, 1.04) | 0.1187 |
| rs2746149 | 29543334 | 0.12 | 0.11 | 1.39 | (1.20, 1.62) | 1.35E-05 |
| rs16894948 | 29548236 | 0.02 | 0.03 | 0.91 | (0.69, 1.19) | 0.4848 |
| rs2746150 | 29550680 | 0.12 | 0.10 | 1.44 | (1.23, 1.68) | 7.39E-06 |
| rs3094573 | 29554611 | 0.41 | 0.41 | 1.03 | (0.94, 1.14) | 0.5001 |
| rs17415601 | 29560390 | 0.06 | 0.05 | 0.74 | (0.58, 0.93) | 0.0093 |
| rs2066951 | 29562468 | 0.00 | 0.02 | 0.87 | (0.61, 1.24) | 0.4265 |
| rs1233493 | 29566220 | 0.12 | 0.12 | 1.38 | (1.19, 1.60) | 1.69E-05 |
| rs1233492 | 29566456 | 0.28 | 0.26 | 0.93 | (0.83, 1.04) | 0.1759 |
| rs1233491 | 29569709 | 0.12 | 0.11 | 1.37 | (1.18, 1.59) | 4.69E-05 |
| rs1233490 | 29569893 | 0.28 | 0.26 | 0.93 | (0.83, 1.04) | 0.1695 |
| rs1233489 | 29571022 | 0.12 | 0.11 | 1.35 | (1.16, 1.57) | 9.39E-05 |
| rs1345227 | 29574935 | 0.38 | 0.37 | 1.03 | (0.93, 1.13) | 0.5967 |
| rs1233487 | 29576788 | 0.31 | 0.28 | 1.04 | (0.93, 1.16) | 0.5097 |
| rs757256 | 29580895 | 0.43 | 0.45 | 1.09 | (0.98, 1.20) | 0.1039 |
| rs3131020 | 29583881 | 0.43 | 0.45 | 1.10 | (1.00, 1.21) | 0.0637 |
| rs1233478 | 29585800 | 0.16 | 0.19 | 1.22 | (1.08, 1.37) | 0.0013 |
| rs3094572 | 29588972 | 0.38 | 0.39 | 1.02 | (0.92, 1.12) | 0.7524 |
| rs1592410 | 29591947 | 0.43 | 0.45 | 1.08 | (0.98, 1.20) | 0.1139 |
| rs9257890 | 29594887 | 0.19 | 0.15 | 0.81 | (0.70, 0.93) | 0.0026 |
| rs724078 | 29597027 | 0.30 | 0.33 | 0.93 | (0.84, 1.03) | 0.1549 |
| rs969931 | 29602876 | 0.47 | 0.49 | 1.15 | (1.04, 1.26) | 0.0054 |
| rs414282 | 29614239 | 0.47 | 0.46 | 1.15 | (1.04, 1.26) | 0.0064 |
| rs362540 | 29618304 | 0.48 | 0.49 | 0.87 | (0.79, 0.96) | 0.0056 |
| rs407161 | 29621786 | 0.48 | 0.49 | 0.88 | (0.80, 0.97) | 0.0093 |
| rs398616 | 29622209 | 0.48 | 0.49 | 1.14 | (1.04, 1.26) | 0.0058 |
| rs3094576 | 29624221 | 0.16 | 0.14 | 0.94 | (0.81, 1.08) | 0.3653 |
| rs376681 | 29625072 | 0.48 | 0.48 | 1.13 | (1.02, 1.24) | 0.0178 |
| rs453658 | 29627827 | 0.48 | 0.48 | 1.13 | (1.03, 1.25) | 0.0119 |
| rs2534791 | 29630691 | 0.27 | 0.26 | 0.91 | (0.82, 1.02) | 0.1221 |
| rs404240 | 29631936 | 0.13 | 0.12 | 1.42 | (1.22, 1.65) | 7.30E-06 |
| rs2534790 | 29632147 | 0.27 | 0.26 | 0.92 | (0.82, 1.03) | 0.1458 |
| rs362536 | 29634919 | 0.48 | 0.50 | 0.88 | (0.80, 0.97) | 0.0113 |
| rs64036 | 29635246 | 0.00 | 0.02 | 1.01 | (0.72, 1.41) | 0.9600 |
| rs362513 | 29636297 | 0.48 | 0.46 | 1.13 | (1.03, 1.25) | 0.0110 |
| rs1233405 | 29637733 | 0.48 | 0.47 | 1.16 | (1.05, 1.28) | 0.0030 |
| rs362527 | 29639797 | 0.07 | 0.05 | 0.96 | (0.78, 1.20) | 0.7317 |
| rs2523447 | 29640718 | 0.07 | 0.04 | 0.89 | (0.68, 1.17) | 0.4049 |
| rs388234 | 29641274 | 0.38 | 0.36 | 0.97 | (0.87, 1.07) | 0.5399 |
| rs3094577 | 29641849 | 0.34 | 0.34 | 0.98 | (0.88, 1.08) | 0.6717 |
| rs1119080 | 29644664 | 0.38 | 0.36 | 0.97 | (0.88, 1.07) | 0.5252 |
| rs1235162 | 29645203 | 0.13 | 0.12 | 1.40 | (1.21, 1.62) | 5.57E-06 |
| rs1233399 | 29647461 | 0.26 | 0.19 | 0.98 | (0.87, 1.12) | 0.8111 |
| rs1003581 | 29648183 | 0.13 | 0.16 | 0.92 | (0.81, 1.05) | 0.2237 |
| rs362509 | 29648840 | 0.38 | 0.36 | 0.96 | (0.87, 1.07) | 0.4803 |
| rs362525 | 29651625 | 0.13 | 0.16 | 0.91 | (0.79, 1.04) | 0.1586 |
| rs1233397 | 29653694 | 0.26 | 0.21 | 0.91 | (0.80, 1.03) | 0.1250 |
| rs1233394 | 29657240 | 0.15 | 0.13 | 1.05 | (0.91, 1.22) | 0.4780 |
| rs1233391 | 29658597 | 0.22 | 0.20 | 0.92 | (0.82, 1.04) | 0.1979 |
| rs1233388 | 29661706 | 0.22 | 0.21 | 0.91 | (0.80, 1.02) | 0.1107 |
| rs362521 | 29664738 | 0.07 | 0.06 | 0.69 | (0.56, 0.87) | 0.0010 |
| rs1233386 | 29666169 | 0.15 | 0.14 | 1.01 | (0.87, 1.16) | 0.9298 |
| rs1233384 | 29667274 | 0.15 | 0.14 | 1.01 | (0.87, 1.16) | 0.9359 |
| rs17508548 | 29671046 | 0.10 | 0.12 | 0.85 | (0.74, 0.99) | 0.0380 |
| rs9468559 | 29673266 | 0.00 | 0.02 | 1.10 | (0.76, 1.59) | 0.6197 |
| rs3025643 | 29677934 | 0.35 | 0.30 | 0.96 | (0.86, 1.07) | 0.4517 |
| rs2267633 | 29678820 | 0.10 | 0.12 | 0.86 | (0.74, 1.00) | 0.0518 |
| rs10946999 | 29679588 | 0.13 | 0.12 | 0.81 | (0.70, 0.95) | 0.0073 |
| rs740884 | 29682610 | 0.07 | 0.06 | 0.94 | (0.76, 1.17) | 0.5763 |
| rs740882 | 29683435 | 0.10 | 0.11 | 0.86 | (0.74, 1.01) | 0.0727 |
| rs29230 | 29684372 | 0.17 | 0.18 | 0.89 | (0.78, 1.02) | 0.0954 |
| rs29262 | 29684571 | 0.10 | 0.09 | 1.00 | (0.84, 1.19) | 0.9931 |
| rs29258 | 29685596 | 0.10 | 0.10 | 0.97 | (0.82, 1.14) | 0.6854 |
| rs29257 | 29686009 | 0.10 | 0.10 | 1.00 | (0.85, 1.17) | 0.9821 |
| rs29255 | 29687523 | 0.07 | 0.06 | 0.74 | (0.59, 0.92) | 0.0063 |
| rs29227 | 29688567 | 0.10 | 0.10 | 1.00 | (0.85, 1.19) | 0.9823 |
| rs6919973 | 29695853 | 0.10 | 0.10 | 0.98 | (0.83, 1.15) | 0.7908 |
| rs29223 | 29696523 | 0.10 | 0.10 | 1.00 | (0.85, 1.17) | 0.9899 |
| rs715044 | 29701767 | 0.07 | 0.06 | 0.69 | (0.54, 0.87) | 0.0016 |
| rs2021749 | 29706098 | 0.10 | 0.10 | 0.99 | (0.85, 1.17) | 0.9414 |
| rs9461540 | 29712243 | 0.12 | 0.15 | 0.89 | (0.77, 1.02) | 0.1005 |
| rs3095267 | 29715025 | 0.24 | 0.22 | 1.11 | (0.99, 1.25) | 0.0739 |
| rs29218 | 29715408 | 0.24 | 0.22 | 1.10 | (0.98, 1.24) | 0.1014 |
| rs1233374 | 29716673 | 0.08 | 0.10 | 0.98 | (0.83, 1.15) | 0.7692 |
| rs29273 | 29718968 | 0.17 | 0.18 | 0.91 | (0.80, 1.03) | 0.1341 |
| rs29232 | 29719410 | 0.33 | 0.37 | 0.85 | (0.77, 0.94) | 0.0020 |
| rs3129073 | 29723801 | 0.24 | 0.21 | 1.10 | (0.98, 1.24) | 0.1148 |
| rs439812 | 29724586 | 0.31 | 0.21 | 0.99 | (0.88, 1.12) | 0.9148 |
| rs29269 | 29725726 | 0.17 | 0.19 | 0.90 | (0.79, 1.02) | 0.0810 |
| rs29228 | 29731718 | 0.24 | 0.22 | 1.10 | (0.98, 1.24) | 0.1052 |
| rs29234 | 29732091 | 0.05 | 0.05 | 1.05 | (0.84, 1.31) | 0.6885 |
| rs3130250 | 29732980 | 0.11 | 0.15 | 0.99 | (0.87, 1.13) | 0.9027 |
| rs2535260 | 29736862 | 0.18 | 0.20 | 0.93 | (0.82, 1.05) | 0.2130 |
| rs2256266 | 29740297 | 0.31 | 0.21 | 0.98 | (0.87, 1.10) | 0.7493 |
| rs17434385 | 29743389 | 0.03 | 0.03 | 0.73 | (0.54, 0.99) | 0.0411 |
| rs2071653 | 29743907 | 0.14 | 0.20 | 1.00 | (0.89, 1.13) | 0.9935 |
| rs1122947 | 29746413 | 0.16 | 0.18 | 0.87 | (0.77, 1.00) | 0.0426 |
| rs375984 | 29752481 | 0.28 | 0.23 | 1.17 | (1.04, 1.31) | 0.0108 |
| rs2747421 | 29753097 | 0.28 | 0.24 | 1.13 | (1.01, 1.27) | 0.0312 |
| rs396660 | 29754144 | 0.34 | 0.33 | 1.10 | (1.00, 1.22) | 0.0601 |
| rs445150 | 29754858 | 0.34 | 0.33 | 1.10 | (1.00, 1.22) | 0.0591 |
| rs2535236 | 29759664 | 0.27 | 0.32 | 0.94 | (0.84, 1.04) | 0.2057 |
| rs3117294 | 29761308 | 0.29 | 0.35 | 0.95 | (0.85, 1.05) | 0.2779 |
| rs7772169 | 29769435 | 0.43 | 0.42 | 0.97 | (0.88, 1.07) | 0.5609 |
| rs3131886 | 29772431 | 0.48 | 0.46 | 1.01 | (0.91, 1.11) | 0.9202 |
| rs7382153 | 29774090 | 0.37 | 0.49 | 1.06 | (0.96, 1.16) | 0.2789 |
| rs7776082 | 29775252 | 0.48 | 0.46 | 1.03 | (0.93, 1.14) | 0.5684 |
| rs9258102 | 29776892 | 0.16 | 0.19 | 0.92 | (0.81, 1.04) | 0.1584 |
| rs9258117 | 29779074 | 0.16 | 0.17 | 0.89 | (0.78, 1.02) | 0.0858 |
| rs3131863 | 29781462 | 0.18 | 0.28 | 1.05 | (0.94, 1.17) | 0.4176 |
| rs1476572 | 29782720 | 0.18 | 0.27 | 1.04 | (0.93, 1.16) | 0.4628 |
| rs1632964 | 29790218 | 0.30 | 0.25 | 1.08 | (0.97, 1.21) | 0.1782 |
| rs2524272 | 29790379 | 0.18 | 0.28 | 1.03 | (0.92, 1.14) | 0.6202 |
| rs1627567 | 29790845 | 0.18 | 0.28 | 1.03 | (0.92, 1.15) | 0.6051 |
| rs1632962 | 29791787 | 0.30 | 0.26 | 1.07 | (0.96, 1.20) | 0.2067 |
| rs2517911 | 29792613 | 0.30 | 0.27 | 1.08 | (0.97, 1.21) | 0.1772 |
| rs7759272 | 29792973 | 0.08 | 0.12 | 1.04 | (0.90, 1.21) | 0.5706 |
| rs9258158 | 29796159 | 0.08 | 0.12 | 1.04 | (0.90, 1.21) | 0.5741 |
| rs3757325 | 29797181 | 0.08 | 0.12 | 1.05 | (0.90, 1.22) | 0.5620 |
| rs1362126 | 29798998 | 0.45 | 0.40 | 0.94 | (0.85, 1.04) | 0.2658 |
| rs1736926 | 29800541 | 0.13 | 0.17 | 1.00 | (0.88, 1.14) | 0.9933 |
| rs2076177 | 29801092 | 0.07 | 0.12 | 1.03 | (0.89, 1.20) | 0.6701 |
| rs2235383 | 29801478 | 0.07 | 0.12 | 1.04 | (0.89, 1.21) | 0.6076 |
| rs1628578 | 29803481 | 0.30 | 0.27 | 1.09 | (0.97, 1.21) | 0.1439 |
| rs3817826 | 29804097 | 0.07 | 0.11 | 1.05 | (0.91, 1.23) | 0.4999 |
| rs3736694 | 29804828 | 0.07 | 0.11 | 1.08 | (0.92, 1.27) | 0.3369 |
| rs1611350 | 29806800 | 0.33 | 0.31 | 1.09 | (0.98, 1.21) | 0.1174 |
| rs2523402 | 29807135 | 0.47 | 0.40 | 0.95 | (0.86, 1.05) | 0.3085 |
| rs6910733 | 29807364 | 0.05 | 0.12 | 1.02 | (0.87, 1.19) | 0.8095 |
| rs2523400 | 29807763 | 0.44 | 0.46 | 1.04 | (0.94, 1.14) | 0.4869 |
| rs2523399 | 29808058 | 0.44 | 0.45 | 1.05 | (0.95, 1.17) | 0.3429 |
| rs1610601 | 29808162 | 0.28 | 0.25 | 1.09 | (0.97, 1.22) | 0.1451 |
| rs2735054 | 29808594 | 0.44 | 0.46 | 1.04 | (0.94, 1.14) | 0.4643 |
| rs2735052 | 29809543 | 0.49 | 0.43 | 0.95 | (0.87, 1.05) | 0.3421 |
| rs2523395 | 29810489 | 0.49 | 0.43 | 0.93 | (0.84, 1.03) | 0.1397 |
| rs2394159 | 29810658 | 0.47 | 0.42 | 0.93 | (0.84, 1.03) | 0.1420 |
| rs2394160 | 29811241 | 0.49 | 0.42 | 0.95 | (0.86, 1.05) | 0.3180 |
| rs9258205 | 29811802 | 0.07 | 0.12 | 1.02 | (0.87, 1.19) | 0.8169 |
| rs7751815 | 29813063 | 0.07 | 0.11 | 1.05 | (0.90, 1.22) | 0.5537 |
| rs1610603 | 29816201 | 0.13 | 0.17 | 1.00 | (0.88, 1.13) | 0.9763 |
| rs1610613 | 29818942 | 0.13 | 0.17 | 1.01 | (0.89, 1.15) | 0.8802 |
| rs1633088 | 29821559 | 0.29 | 0.27 | 1.07 | (0.96, 1.20) | 0.2145 |
| rs909728 | 29827540 | 0.45 | 0.40 | 1.00 | (0.90, 1.11) | 0.9580 |
| rs9391630 | 29830494 | 0.32 | 0.21 | 0.98 | (0.87, 1.10) | 0.7253 |
| rs1610628 | 29836188 | 0.13 | 0.17 | 1.00 | (0.88, 1.14) | 0.9766 |
| rs1737069 | 29838709 | 0.18 | 0.25 | 1.06 | (0.94, 1.18) | 0.3198 |
| rs1737043 | 29844026 | 0.19 | 0.29 | 0.95 | (0.85, 1.05) | 0.3021 |
| rs1610726 | 29846644 | 0.36 | 0.33 | 1.13 | (1.02, 1.26) | 0.0167 |
| rs1362068 | 29850087 | 0.38 | 0.39 | 1.10 | (1.00, 1.22) | 0.0595 |
| rs2517930 | 29853054 | 0.39 | 0.31 | 0.92 | (0.83, 1.03) | 0.1318 |
| rs1633019 | 29855540 | 0.36 | 0.34 | 1.13 | (1.02, 1.26) | 0.0196 |
| rs1002044 | 29861708 | 0.33 | 0.31 | 1.12 | (1.01, 1.25) | 0.0341 |
| rs6921921 | 29863430 | 0.17 | 0.23 | 1.05 | (0.93, 1.17) | 0.4495 |
| rs1633013 | 29865974 | 0.33 | 0.32 | 1.11 | (1.00, 1.23) | 0.0579 |
| rs1737002 | 29870882 | 0.33 | 0.30 | 1.12 | (1.00, 1.24) | 0.0436 |
| rs1736994 | 29874575 | 0.33 | 0.30 | 1.14 | (1.03, 1.28) | 0.0149 |
| rs7741100 | 29876102 | 0.07 | 0.05 | 0.91 | (0.73, 1.14) | 0.4164 |
| rs1610714 | 29877808 | 0.33 | 0.31 | 1.09 | (0.98, 1.21) | 0.1088 |
| rs1736981 | 29878301 | 0.19 | 0.29 | 0.97 | (0.87, 1.08) | 0.5432 |
| rs1736980 | 29879858 | 0.27 | 0.26 | 1.16 | (1.03, 1.29) | 0.0110 |
| rs3998758 | 29883641 | 0.43 | 0.35 | 0.94 | (0.84, 1.04) | 0.2037 |
| rs1610703 | 29889592 | 0.38 | 0.38 | 1.08 | (0.97, 1.19) | 0.1627 |
| rs1633105 | 29891361 | 0.45 | 0.43 | 1.04 | (0.94, 1.15) | 0.4232 |
| rs2735028 | 29893517 | 0.37 | 0.38 | 1.05 | (0.95, 1.16) | 0.3674 |
| rs1633017 | 29896749 | 0.08 | 0.14 | 0.96 | (0.83, 1.11) | 0.5698 |
| rs1610678 | 29897169 | 0.50 | 0.49 | 0.92 | (0.84, 1.02) | 0.1175 |
| rs2394694 | 29897978 | 0.50 | 0.48 | 0.92 | (0.84, 1.02) | 0.1073 |
| rs1736935 | 29902422 | 0.50 | 0.48 | 0.92 | (0.83, 1.02) | 0.1080 |
| rs1736920 | 29905152 | 0.50 | 0.48 | 0.91 | (0.83, 1.01) | 0.0701 |
| rs1632933 | 29905912 | 0.48 | 0.48 | 1.10 | (1.00, 1.21) | 0.0589 |
| rs915668 | 29906438 | 0.50 | 0.48 | 0.91 | (0.83, 1.00) | 0.0600 |
| rs1063320 | 29906728 | 0.50 | 0.48 | 0.91 | (0.83, 1.01) | 0.0696 |
| rs1233330 | 29907082 | 0.08 | 0.14 | 0.96 | (0.83, 1.12) | 0.6168 |
| rs2517896 | 29908121 | 0.09 | 0.09 | 0.93 | (0.79, 1.10) | 0.4135 |
| rs2517892 | 29909453 | 0.25 | 0.28 | 0.97 | (0.87, 1.09) | 0.6482 |
| rs2523793 | 29910529 | 0.21 | 0.22 | 0.91 | (0.80, 1.03) | 0.1307 |
| rs2523790 | 29911629 | 0.25 | 0.27 | 0.97 | (0.87, 1.09) | 0.6409 |
| rs2394180 | 29913178 | 0.12 | 0.12 | 0.95 | (0.82, 1.10) | 0.4797 |
| rs2735014 | 29913788 | 0.20 | 0.20 | 0.92 | (0.81, 1.04) | 0.1988 |
| rs2254077 | 29914173 | 0.25 | 0.28 | 0.96 | (0.86, 1.08) | 0.5333 |
| rs2517887 | 29915372 | 0.21 | 0.21 | 0.93 | (0.82, 1.05) | 0.2520 |
| rs2735005 | 29916443 | 0.25 | 0.28 | 0.96 | (0.86, 1.07) | 0.4349 |
| rs2735003 | 29916613 | 0.21 | 0.21 | 0.96 | (0.85, 1.09) | 0.5774 |
| rs2523776 | 29917173 | 0.09 | 0.09 | 0.94 | (0.79, 1.11) | 0.4792 |
| rs2734990 | 29920484 | 0.23 | 0.24 | 0.95 | (0.85, 1.06) | 0.3775 |
| rs3900268 | 29921359 | 0.28 | 0.26 | 1.15 | (1.03, 1.28) | 0.0158 |
| rs3873260 | 29921661 | 0.23 | 0.24 | 0.95 | (0.85, 1.07) | 0.4387 |
| rs3128912 | 29923616 | 0.08 | 0.14 | 0.97 | (0.84, 1.12) | 0.6961 |
| rs2394186 | 29924400 | 0.23 | 0.14 | 0.93 | (0.81, 1.08) | 0.3476 |
| rs2734985 | 29926641 | 0.25 | 0.25 | 1.19 | (1.06, 1.33) | 0.0032 |
| rs2734981 | 29929671 | 0.25 | 0.26 | 1.19 | (1.06, 1.33) | 0.0023 |
| rs2734980 | 29929875 | 0.25 | 0.25 | 1.18 | (1.06, 1.32) | 0.0029 |
| rs2428510 | 29931006 | 0.43 | 0.44 | 1.10 | (0.99, 1.21) | 0.0599 |
| rs6919513 | 29931973 | 0.43 | 0.44 | 1.09 | (0.98, 1.20) | 0.0998 |
| rs3094657 | 29936289 | 0.17 | 0.23 | 0.98 | (0.87, 1.10) | 0.6937 |
| rs1611723 | 29938484 | 0.40 | 0.41 | 1.07 | (0.97, 1.18) | 0.1802 |
| rs1611732 | 29938987 | 0.43 | 0.44 | 1.09 | (0.98, 1.20) | 0.0936 |
| rs1611737 | 29939550 | 0.43 | 0.45 | 1.08 | (0.98, 1.19) | 0.1206 |
| rs2508046 | 29941012 | 0.13 | 0.14 | 0.92 | (0.80, 1.06) | 0.2320 |
| rs2734971 | 29942428 | 0.47 | 0.47 | 0.89 | (0.81, 0.99) | 0.0277 |
| rs2844821 | 29946621 | 0.37 | 0.27 | 0.92 | (0.82, 1.03) | 0.1450 |
| rs886398 | 29947247 | 0.10 | 0.09 | 0.95 | (0.80, 1.13) | 0.5702 |
| rs1611647 | 29948135 | 0.21 | 0.18 | 1.19 | (1.04, 1.36) | 0.0101 |
| rs2523809 | 29957598 | 0.13 | 0.12 | 1.00 | (0.86, 1.16) | 0.9679 |
| rs1611627 | 29965650 | 0.43 | 0.42 | 1.06 | (0.96, 1.18) | 0.2289 |
| rs2860580 | 30014670 | 0.35 | 0.41 | 1.21 | (1.09, 1.34) | 0.0003 |
| rs3094141 | 30017786 | 0.08 | 0.13 | 1.01 | (0.88, 1.17) | 0.8594 |
| rs3823339 | 30020947 | 0.07 | 0.05 | 0.99 | (0.78, 1.26) | 0.9540 |
| rs1061235 | 30021277 | 0.03 | 0.03 | 1.18 | (0.89, 1.54) | 0.2472 |
| rs2499 | 30021521 | 0.08 | 0.13 | 0.97 | (0.84, 1.12) | 0.6391 |
| rs7745413 | 30023448 | 0.22 | 0.19 | 0.95 | (0.84, 1.08) | 0.4521 |
| rs1632876 | 30026598 | 0.08 | 0.14 | 0.97 | (0.84, 1.13) | 0.7243 |
| rs1655912 | 30028444 | 0.08 | 0.16 | 1.02 | (0.89, 1.16) | 0.8099 |
| rs3893538 | 30032404 | 0.20 | 0.19 | 0.86 | (0.76, 0.98) | 0.0253 |
| rs2571400 | 30035701 | 0.48 | 0.49 | 0.93 | (0.84, 1.02) | 0.1168 |
| rs7739434 | 30038598 | 0.09 | 0.18 | 0.98 | (0.87, 1.11) | 0.7695 |
| rs3873283 | 30040979 | 0.10 | 0.15 | 0.99 | (0.86, 1.13) | 0.8605 |
| rs6457109 | 30041240 | 0.05 | 0.11 | 0.93 | (0.79, 1.09) | 0.3480 |
| rs4713270 | 30042676 | 0.36 | 0.28 | 0.93 | (0.83, 1.04) | 0.1785 |
| rs12193100 | 30044893 | 0.36 | 0.28 | 0.92 | (0.82, 1.03) | 0.1470 |
| rs2517673 | 30045221 | 0.09 | 0.09 | 1.13 | (0.95, 1.35) | 0.1623 |
| rs2523969 | 30046756 | 0.12 | 0.14 | 0.99 | (0.86, 1.15) | 0.9523 |
| rs2735079 | 30046911 | 0.29 | 0.37 | 0.99 | (0.89, 1.10) | 0.8247 |
| rs2523962 | 30047523 | 0.35 | 0.31 | 1.12 | (1.01, 1.25) | 0.0344 |
| rs2523957 | 30048239 | 0.29 | 0.42 | 0.91 | (0.81, 1.01) | 0.0680 |
| rs2256902 | 30048351 | 0.08 | 0.09 | 1.04 | (0.88, 1.24) | 0.6267 |
| rs5009448 | 30048467 | 0.21 | 0.28 | 0.98 | (0.88, 1.10) | 0.7618 |
| rs2256919 | 30048729 | 0.29 | 0.40 | 0.97 | (0.88, 1.07) | 0.5263 |
| rs2245961 | 30051983 | 0.23 | 0.36 | 0.98 | (0.89, 1.09) | 0.7267 |
| rs2844802 | 30056791 | 0.32 | 0.29 | 1.09 | (0.98, 1.22) | 0.0999 |
| rs379221 | 30058119 | 0.11 | 0.15 | 0.99 | (0.87, 1.14) | 0.9546 |
| rs2735067 | 30059085 | 0.32 | 0.30 | 1.10 | (0.99, 1.23) | 0.0727 |
| rs4947244 | 30062343 | 0.36 | 0.27 | 0.93 | (0.83, 1.04) | 0.1910 |
| rs5025708 | 30063178 | 0.07 | 0.12 | 0.96 | (0.83, 1.12) | 0.6069 |
| rs9260973 | 30069559 | 0.07 | 0.10 | 0.98 | (0.83, 1.16) | 0.8218 |
| rs6457121 | 30071044 | 0.49 | 0.49 | 0.94 | (0.85, 1.03) | 0.1772 |
| rs6911279 | 30073323 | 0.09 | 0.12 | 0.94 | (0.81, 1.09) | 0.4226 |
| rs7758512 | 30078568 | 0.09 | 0.12 | 0.96 | (0.83, 1.11) | 0.5770 |
| rs7750714 | 30082109 | 0.07 | 0.05 | 0.88 | (0.70, 1.10) | 0.2472 |
| rs2240619 | 30083432 | 0.49 | 0.49 | 0.94 | (0.86, 1.04) | 0.2307 |
| rs356973 | 30090003 | 0.04 | 0.06 | 1.00 | (0.81, 1.24) | 0.9964 |
| rs9261145 | 30092844 | 0.09 | 0.12 | 0.96 | (0.82, 1.11) | 0.5539 |
| rs3869065 | 30095453 | 0.49 | 0.49 | 0.94 | (0.85, 1.03) | 0.1707 |
| rs165255 | 30097674 | 0.32 | 0.28 | 1.10 | (0.98, 1.23) | 0.1012 |
| rs166325 | 30101175 | 0.06 | 0.06 | 1.02 | (0.82, 1.26) | 0.8587 |
| rs7746866 | 30106161 | 0.09 | 0.12 | 0.96 | (0.83, 1.12) | 0.6430 |
| rs259937 | 30115472 | 0.06 | 0.06 | 0.98 | (0.79, 1.22) | 0.8739 |
| rs259938 | 30115982 | 0.36 | 0.33 | 1.09 | (0.98, 1.21) | 0.0962 |
| rs259939 | 30119560 | 0.36 | 0.33 | 1.11 | (1.00, 1.23) | 0.0535 |
| rs259942 | 30123146 | 0.15 | 0.13 | 0.96 | (0.83, 1.11) | 0.6066 |
| rs259945 | 30127185 | 0.08 | 0.08 | 1.01 | (0.85, 1.21) | 0.8929 |
| rs7761314 | 30130132 | 0.07 | 0.10 | 0.92 | (0.78, 1.10) | 0.3636 |
| rs3869070 | 30131847 | 0.41 | 0.47 | 0.92 | (0.83, 1.01) | 0.0829 |
| rs259919 | 30133482 | 0.40 | 0.32 | 1.11 | (1.00, 1.23) | 0.0496 |
| rs3132129 | 30135839 | 0.07 | 0.09 | 1.03 | (0.87, 1.21) | 0.7629 |
| rs3757329 | 30136403 | 0.07 | 0.10 | 0.90 | (0.76, 1.07) | 0.2182 |
| rs7769930 | 30136786 | 0.07 | 0.08 | 1.01 | (0.85, 1.21) | 0.8820 |
| rs1150741 | 30137630 | 0.32 | 0.30 | 1.08 | (0.97, 1.21) | 0.1356 |
| rs1150740 | 30138668 | 0.08 | 0.09 | 0.98 | (0.82, 1.17) | 0.8201 |
| rs1150739 | 30139324 | 0.42 | 0.42 | 1.09 | (0.98, 1.20) | 0.0929 |
| rs3807031 | 30141863 | 0.31 | 0.19 | 0.97 | (0.86, 1.09) | 0.5827 |
| rs2074479 | 30148988 | 0.09 | 0.12 | 0.96 | (0.82, 1.11) | 0.5696 |
| rs3807033 | 30151934 | 0.05 | 0.09 | 1.05 | (0.89, 1.24) | 0.5703 |
| rs3807035 | 30152806 | 0.12 | 0.15 | 0.98 | (0.85, 1.13) | 0.7527 |
| rs1150735 | 30153178 | 0.40 | 0.37 | 1.13 | (1.02, 1.25) | 0.0200 |
| rs2394734 | 30154225 | 0.14 | 0.17 | 0.99 | (0.87, 1.12) | 0.8565 |
| rs3134881 | 30154343 | 0.07 | 0.10 | 0.93 | (0.78, 1.10) | 0.3704 |
| rs7382061 | 30155944 | 0.43 | 0.40 | 0.91 | (0.83, 1.01) | 0.0726 |
| rs6909253 | 30163622 | 0.43 | 0.40 | 0.92 | (0.83, 1.02) | 0.1024 |
| rs1264708 | 30165133 | 0.20 | 0.22 | 0.98 | (0.87, 1.10) | 0.7401 |
| rs9261387 | 30169340 | 0.07 | 0.08 | 1.01 | (0.84, 1.21) | 0.9309 |
| rs6923832 | 30170037 | 0.07 | 0.06 | 0.91 | (0.74, 1.13) | 0.4132 |
| rs6457144 | 30171347 | 0.43 | 0.39 | 0.88 | (0.79, 0.97) | 0.0137 |
| rs1156534 | 30173131 | 0.18 | 0.19 | 0.99 | (0.88, 1.12) | 0.8861 |
| rs916570 | 30174010 | 0.27 | 0.28 | 1.16 | (1.04, 1.29) | 0.0086 |
| rs1264701 | 30174337 | 0.18 | 0.19 | 1.00 | (0.89, 1.13) | 0.9638 |
| rs1264698 | 30175156 | 0.24 | 0.26 | 1.14 | (1.02, 1.27) | 0.0237 |
| rs7763159 | 30175660 | 0.07 | 0.12 | 0.92 | (0.79, 1.07) | 0.2952 |
| rs2240070 | 30179089 | 0.38 | 0.31 | 0.94 | (0.85, 1.04) | 0.2512 |
| rs2248289 | 30180700 | 0.24 | 0.26 | 1.10 | (0.99, 1.23) | 0.0903 |
| rs2023472 | 30183843 | 0.38 | 0.42 | 1.11 | (1.01, 1.23) | 0.0299 |
| rs2240068 | 30184609 | 0.48 | 0.41 | 1.01 | (0.91, 1.11) | 0.8305 |
| rs2074483 | 30184734 | 0.46 | 0.43 | 1.06 | (0.96, 1.17) | 0.2961 |
| rs2523989 | 30186254 | 0.20 | 0.16 | 1.38 | (1.21, 1.57) | 9.92E-07 |
| rs2239529 | 30186309 | 0.20 | 0.16 | 1.38 | (1.22, 1.57) | 8.32E-07 |
| rs3734838 | 30188210 | 0.09 | 0.09 | 0.85 | (0.72, 1.01) | 0.0653 |
| rs2523984 | 30189982 | 0.20 | 0.17 | 1.35 | (1.19, 1.53) | 3.35E-06 |
| rs2523979 | 30191494 | 0.18 | 0.13 | 1.52 | (1.30, 1.77) | 1.06E-07 |
| rs2523976 | 30192675 | 0.18 | 0.15 | 1.40 | (1.23, 1.60) | 5.02E-07 |
| rs1015465 | 30194319 | 0.11 | 0.13 | 0.85 | (0.74, 0.99) | 0.0306 |
| rs9261434 | 30195755 | 0.08 | 0.12 | 0.93 | (0.80, 1.08) | 0.3621 |
| rs10947055 | 30201343 | 0.05 | 0.08 | 0.89 | (0.74, 1.07) | 0.2040 |
| rs1419673 | 30204696 | 0.30 | 0.22 | 0.94 | (0.84, 1.06) | 0.3045 |
| rs9261457 | 30208200 | 0.30 | 0.22 | 0.94 | (0.83, 1.05) | 0.2704 |
| rs1362104 | 30209635 | 0.39 | 0.40 | 1.19 | (1.08, 1.31) | 0.0006 |
| rs2523995 | 30210163 | 0.12 | 0.12 | 0.72 | (0.61, 0.85) | 4.64E-05 |
| rs1541268 | 30211373 | 0.30 | 0.22 | 0.94 | (0.84, 1.06) | 0.3098 |
| rs2021723 | 30211902 | 0.12 | 0.13 | 0.85 | (0.73, 0.99) | 0.0319 |
| rs2107202 | 30213722 | 0.19 | 0.25 | 0.87 | (0.77, 0.98) | 0.0243 |
| rs2857440 | 30214472 | 0.30 | 0.23 | 0.93 | (0.83, 1.04) | 0.2029 |
| rs9261488 | 30217391 | 0.30 | 0.22 | 0.95 | (0.84, 1.07) | 0.3648 |
| rs9261508 | 30220683 | 0.32 | 0.24 | 0.94 | (0.83, 1.05) | 0.2581 |
| rs3815081 | 30222053 | 0.13 | 0.14 | 0.97 | (0.85, 1.12) | 0.7184 |
| rs3132676 | 30224057 | 0.12 | 0.14 | 0.93 | (0.80, 1.07) | 0.2980 |
| rs1573298 | 30224139 | 0.32 | 0.24 | 0.93 | (0.82, 1.04) | 0.1954 |
| rs1557608 | 30226561 | 0.37 | 0.27 | 0.95 | (0.85, 1.06) | 0.3701 |
| rs9468692 | 30227869 | 0.05 | 0.08 | 0.89 | (0.75, 1.07) | 0.2060 |
| rs2285797 | 30228099 | 0.32 | 0.23 | 0.93 | (0.82, 1.04) | 0.1905 |
| rs2517646 | 30230554 | 0.26 | 0.33 | 0.85 | (0.76, 0.94) | 0.0021 |
| rs2517645 | 30230602 | 0.21 | 0.17 | 1.37 | (1.21, 1.56) | 1.33E-06 |
| rs9261535 | 30235302 | 0.30 | 0.21 | 0.94 | (0.83, 1.07) | 0.3472 |
| rs12212092 | 30236421 | 0.05 | 0.08 | 0.79 | (0.66, 0.95) | 0.0106 |
| rs2157678 | 30236928 | 0.14 | 0.13 | 1.47 | (1.28, 1.69) | 9.39E-08 |
| rs2523734 | 30237655 | 0.12 | 0.14 | 0.83 | (0.71, 0.95) | 0.0083 |
| rs2074477 | 30240014 | 0.13 | 0.15 | 1.00 | (0.87, 1.14) | 0.9769 |
| rs9368624 | 30243956 | 0.15 | 0.17 | 0.99 | (0.87, 1.12) | 0.8284 |
| rs1029239 | 30246141 | 0.46 | 0.46 | 1.12 | (1.02, 1.24) | 0.0168 |
| rs6905949 | 30248504 | 0.18 | 0.17 | 0.87 | (0.77, 1.00) | 0.0425 |
| rs9380156 | 30249021 | 0.13 | 0.15 | 0.98 | (0.86, 1.13) | 0.8171 |
| rs757257 | 30250437 | 0.46 | 0.46 | 1.14 | (1.04, 1.26) | 0.0057 |
| rs2844786 | 30252399 | 0.33 | 0.29 | 0.94 | (0.84, 1.05) | 0.2386 |
| rs7774730 | 30253719 | 0.46 | 0.46 | 1.16 | (1.05, 1.28) | 0.0034 |
| rs2844782 | 30258022 | 0.33 | 0.28 | 0.91 | (0.81, 1.03) | 0.1202 |
| rs1042338 | 30260768 | 0.32 | 0.23 | 0.92 | (0.81, 1.04) | 0.1734 |
| rs6457164 | 30263087 | 0.46 | 0.47 | 1.19 | (1.08, 1.32) | 0.0005 |
| rs2074472 | 30264475 | 0.36 | 0.31 | 0.93 | (0.83, 1.04) | 0.1965 |
| rs765977 | 30265633 | 0.46 | 0.46 | 1.14 | (1.04, 1.26) | 0.0063 |
| rs718254 | 30267018 | 0.46 | 0.46 | 1.14 | (1.04, 1.26) | 0.0069 |
| rs2284164 | 30267833 | 0.46 | 0.46 | 1.15 | (1.04, 1.26) | 0.0051 |
| rs2284165 | 30268034 | 0.14 | 0.20 | 0.92 | (0.81, 1.04) | 0.1573 |
| rs4711211 | 30270788 | 0.30 | 0.23 | 0.93 | (0.83, 1.05) | 0.2323 |
| rs2523722 | 30273252 | 0.23 | 0.22 | 1.28 | (1.14, 1.44) | 1.82E-05 |
| rs2523721 | 30274245 | 0.23 | 0.23 | 1.23 | (1.10, 1.39) | 0.0004 |
| rs2072107 | 30274914 | 0.18 | 0.16 | 0.80 | (0.70, 0.92) | 0.0011 |
| rs2239531 | 30276808 | 0.13 | 0.15 | 0.97 | (0.85, 1.11) | 0.6967 |
| rs2517611 | 30277306 | 0.23 | 0.22 | 1.28 | (1.14, 1.43) | 2.04E-05 |
| rs971570 | 30280492 | 0.23 | 0.22 | 1.28 | (1.14, 1.44) | 1.77E-05 |
| rs2523713 | 30281309 | 0.23 | 0.22 | 1.30 | (1.16, 1.46) | 5.74E-06 |
| rs2021722 | 30282110 | 0.23 | 0.23 | 1.27 | (1.13, 1.42) | 3.13E-05 |
| rs3132671 | 30286266 | 0.46 | 0.46 | 1.15 | (1.05, 1.27) | 0.0036 |
| rs1345229 | 30290374 | 0.09 | 0.12 | 0.80 | (0.69, 0.94) | 0.0050 |
| rs3132668 | 30295143 | 0.46 | 0.46 | 1.17 | (1.06, 1.29) | 0.0020 |
| rs3094132 | 30295413 | 0.46 | 0.46 | 1.16 | (1.05, 1.27) | 0.0030 |
| rs3132667 | 30299566 | 0.46 | 0.46 | 1.16 | (1.06, 1.28) | 0.0019 |
| rs2040486 | 30302082 | 0.46 | 0.46 | 1.16 | (1.05, 1.28) | 0.0023 |
| rs3094140 | 30304292 | 0.46 | 0.46 | 1.17 | (1.06, 1.28) | 0.0017 |
| rs1573295 | 30307046 | 0.46 | 0.45 | 1.16 | (1.05, 1.28) | 0.0025 |
| rs916571 | 30309934 | 0.46 | 0.45 | 1.18 | (1.07, 1.30) | 0.0010 |
| rs2844779 | 30313386 | 0.33 | 0.30 | 0.95 | (0.85, 1.05) | 0.3181 |
| rs1362089 | 30317781 | 0.46 | 0.47 | 1.12 | (1.02, 1.24) | 0.0202 |
| rs3094635 | 30320795 | 0.44 | 0.49 | 1.15 | (1.05, 1.27) | 0.0039 |
| rs3132663 | 30322814 | 0.46 | 0.46 | 1.15 | (1.04, 1.26) | 0.0058 |
| rs2516723 | 30329421 | 0.46 | 0.46 | 1.10 | (1.00, 1.22) | 0.0487 |
| rs4526237 | 30332284 | 0.12 | 0.15 | 1.01 | (0.88, 1.15) | 0.9270 |
| rs928824 | 30332868 | 0.10 | 0.08 | 0.88 | (0.73, 1.05) | 0.1419 |
| rs2516714 | 30334283 | 0.34 | 0.39 | 1.22 | (1.11, 1.35) | 6.59E-05 |
| rs2523747 | 30336917 | 0.15 | 0.22 | 0.99 | (0.88, 1.11) | 0.8586 |
| rs2428507 | 30337285 | 0.34 | 0.38 | 1.22 | (1.10, 1.35) | 8.68E-05 |
| rs2844766 | 30338640 | 0.34 | 0.39 | 1.21 | (1.09, 1.33) | 0.0002 |
| rs3094073 | 30339203 | 0.19 | 0.16 | 1.37 | (1.20, 1.56) | 1.53E-06 |
| rs3129701 | 30340651 | 0.19 | 0.17 | 1.38 | (1.21, 1.58) | 1.12E-06 |
| rs3132657 | 30341492 | 0.19 | 0.17 | 1.36 | (1.20, 1.54) | 2.18E-06 |
| rs2844763 | 30343163 | 0.15 | 0.22 | 0.98 | (0.87, 1.10) | 0.7671 |
| rs2844762 | 30344733 | 0.34 | 0.39 | 1.21 | (1.10, 1.34) | 0.0001 |
| rs6457188 | 30346863 | 0.20 | 0.26 | 1.01 | (0.91, 1.13) | 0.8128 |
| rs7453068 | 30348905 | 0.15 | 0.22 | 1.00 | (0.89, 1.12) | 0.9319 |
| rs9357095 | 30350107 | 0.12 | 0.16 | 0.99 | (0.87, 1.13) | 0.8706 |
| rs3132653 | 30351215 | 0.42 | 0.39 | 0.93 | (0.84, 1.03) | 0.1501 |
| rs968909 | 30352558 | 0.20 | 0.26 | 1.02 | (0.92, 1.14) | 0.6719 |
| rs7383543 | 30360815 | 0.34 | 0.26 | 0.96 | (0.85, 1.07) | 0.4264 |
| rs1264624 | 30363068 | 0.20 | 0.25 | 1.01 | (0.90, 1.13) | 0.8422 |
| rs2285800 | 30365476 | 0.34 | 0.25 | 0.96 | (0.86, 1.08) | 0.5164 |
| rs2285799 | 30365672 | 0.05 | 0.03 | 1.10 | (0.85, 1.43) | 0.4322 |
| rs928823 | 30365825 | 0.10 | 0.08 | 0.88 | (0.74, 1.06) | 0.1545 |
| rs3734841 | 30367284 | 0.20 | 0.26 | 1.01 | (0.91, 1.13) | 0.7857 |
| rs1045251 | 30367636 | 0.15 | 0.22 | 0.99 | (0.88, 1.11) | 0.8404 |
| rs11752362 | 30368961 | 0.14 | 0.12 | 0.87 | (0.74, 1.03) | 0.1000 |
| rs1264616 | 30372286 | 0.15 | 0.22 | 1.00 | (0.89, 1.12) | 0.9818 |
| rs261950 | 30377540 | 0.20 | 0.26 | 1.01 | (0.91, 1.13) | 0.7804 |
| rs261946 | 30379313 | 0.34 | 0.25 | 0.96 | (0.86, 1.07) | 0.4603 |
| rs928822 | 30383225 | 0.42 | 0.38 | 0.95 | (0.85, 1.05) | 0.2773 |
| rs9295843 | 30389213 | 0.15 | 0.22 | 1.00 | (0.89, 1.12) | 0.9650 |
| rs6924453 | 30393934 | 0.20 | 0.26 | 1.00 | (0.90, 1.12) | 0.9742 |
| rs6900042 | 30397250 | 0.20 | 0.26 | 1.00 | (0.90, 1.12) | 0.9363 |
| rs1110464 | 30404050 | 0.47 | 0.40 | 0.96 | (0.87, 1.06) | 0.4541 |
| rs3094064 | 30404232 | 0.14 | 0.13 | 1.49 | (1.30, 1.72) | 2.29E-08 |
| rs2057727 | 30405508 | 0.15 | 0.22 | 0.99 | (0.88, 1.11) | 0.8941 |
| rs3778624 | 30410579 | 0.15 | 0.22 | 0.97 | (0.87, 1.09) | 0.6317 |
| rs3129838 | 30414532 | 0.08 | 0.11 | 0.94 | (0.80, 1.09) | 0.3977 |
| rs2240058 | 30418479 | 0.10 | 0.14 | 1.01 | (0.88, 1.16) | 0.8562 |
| rs6931763 | 30419911 | 0.14 | 0.14 | 0.83 | (0.72, 0.96) | 0.0093 |
| rs974962 | 30422586 | 0.10 | 0.14 | 0.96 | (0.84, 1.10) | 0.5616 |
| rs974961 | 30422990 | 0.34 | 0.25 | 0.97 | (0.87, 1.09) | 0.6113 |
| rs9380174 | 30424718 | 0.15 | 0.21 | 1.01 | (0.89, 1.14) | 0.9103 |
| rs3130364 | 30427133 | 0.14 | 0.13 | 1.49 | (1.29, 1.72) | 3.54E-08 |
| rs6905389 | 30427909 | 0.03 | 0.07 | 1.04 | (0.86, 1.26) | 0.6662 |
| rs3094061 | 30429168 | 0.14 | 0.15 | 1.42 | (1.24, 1.62) | 2.33E-07 |
| rs3130374 | 30429315 | 0.14 | 0.14 | 1.41 | (1.22, 1.62) | 2.27E-06 |
| rs984802 | 30429340 | 0.25 | 0.20 | 1.02 | (0.90, 1.14) | 0.7884 |
| rs3094626 | 30431602 | 0.39 | 0.39 | 1.22 | (1.10, 1.34) | 8.85E-05 |
| rs3130379 | 30433510 | 0.19 | 0.21 | 0.87 | (0.77, 0.98) | 0.0202 |
| rs2158285 | 30439006 | 0.12 | 0.14 | 0.98 | (0.85, 1.13) | 0.7601 |
| rs2040450 | 30442318 | 0.12 | 0.12 | 0.98 | (0.84, 1.14) | 0.7156 |
| rs3129808 | 30443533 | 0.19 | 0.21 | 0.86 | (0.77, 0.97) | 0.0150 |
| rs2844746 | 30447581 | 0.30 | 0.36 | 0.89 | (0.81, 0.99) | 0.0260 |
| rs3132636 | 30450491 | 0.46 | 0.44 | 1.22 | (1.10, 1.35) | 0.0001 |
| rs3129820 | 30451548 | 0.14 | 0.14 | 1.45 | (1.27, 1.66) | 7.49E-08 |
| rs3132629 | 30454072 | 0.46 | 0.45 | 1.17 | (1.06, 1.29) | 0.0017 |
| rs3132622 | 30456653 | 0.46 | 0.45 | 1.17 | (1.06, 1.29) | 0.0012 |
| rs2844743 | 30456811 | 0.12 | 0.14 | 0.99 | (0.86, 1.14) | 0.8287 |
| rs6936217 | 30460626 | 0.14 | 0.14 | 1.45 | (1.27, 1.66) | 7.07E-08 |
| rs3094702 | 30467356 | 0.46 | 0.45 | 1.17 | (1.06, 1.28) | 0.0017 |
| rs3130113 | 30471924 | 0.46 | 0.44 | 1.17 | (1.06, 1.29) | 0.0015 |
| rs1264569 | 30473299 | 0.18 | 0.22 | 0.92 | (0.82, 1.04) | 0.1673 |
| rs3130118 | 30473719 | 0.29 | 0.31 | 0.96 | (0.86, 1.07) | 0.4428 |
| rs1034323 | 30476614 | 0.43 | 0.39 | 0.91 | (0.83, 1.01) | 0.0810 |
| rs2023371 | 30480094 | 0.20 | 0.14 | 0.94 | (0.82, 1.09) | 0.4297 |
| rs2023458 | 30482498 | 0.21 | 0.19 | 0.92 | (0.81, 1.04) | 0.1782 |
| rs1264550 | 30485177 | 0.40 | 0.42 | 1.21 | (1.10, 1.33) | 0.0002 |
| rs4713324 | 30488252 | 0.41 | 0.35 | 0.90 | (0.81, 1.00) | 0.0526 |
| rs1150769 | 30490951 | 0.40 | 0.42 | 0.87 | (0.78, 0.96) | 0.0046 |
| rs4713327 | 30491747 | 0.41 | 0.34 | 0.93 | (0.84, 1.03) | 0.1589 |
| rs2074502 | 30492301 | 0.21 | 0.19 | 0.93 | (0.82, 1.06) | 0.2722 |
| rs1264542 | 30494975 | 0.40 | 0.40 | 0.83 | (0.75, 0.92) | 0.0005 |
| rs1268445 | 30495446 | 0.40 | 0.43 | 1.24 | (1.12, 1.38) | 6.67E-05 |
| rs9261947 | 30502607 | 0.20 | 0.13 | 0.95 | (0.82, 1.09) | 0.4616 |
| rs9468728 | 30505068 | 0.20 | 0.14 | 0.96 | (0.84, 1.11) | 0.6173 |
| rs1264521 | 30513935 | 0.11 | 0.15 | 0.95 | (0.83, 1.09) | 0.4368 |
| rs1269556 | 30516766 | 0.39 | 0.43 | 1.18 | (1.07, 1.31) | 0.0007 |
| rs1268444 | 30519525 | 0.40 | 0.40 | 0.85 | (0.77, 0.94) | 0.0015 |
| rs7747320 | 30521631 | 0.20 | 0.14 | 0.96 | (0.83, 1.10) | 0.5794 |
| rs1076832 | 30527810 | 0.21 | 0.16 | 0.91 | (0.79, 1.04) | 0.1499 |
| rs2844729 | 30530611 | 0.40 | 0.40 | 0.85 | (0.77, 0.94) | 0.0019 |
| rs2516677 | 30532602 | 0.40 | 0.45 | 1.19 | (1.08, 1.31) | 0.0005 |
| rs915666 | 30535461 | 0.40 | 0.43 | 1.20 | (1.09, 1.33) | 0.0004 |
| rs7764934 | 30542545 | 0.43 | 0.39 | 0.92 | (0.83, 1.02) | 0.1264 |
| rs2516670 | 30542978 | 0.12 | 0.15 | 0.97 | (0.84, 1.11) | 0.6214 |
| rs9295895 | 30546205 | 0.22 | 0.20 | 0.96 | (0.85, 1.09) | 0.5603 |
| rs6457235 | 30549631 | 0.13 | 0.15 | 0.95 | (0.82, 1.09) | 0.4074 |
| rs7775268 | 30554942 | 0.43 | 0.38 | 0.92 | (0.84, 1.02) | 0.1288 |
| rs6909620 | 30556647 | 0.22 | 0.20 | 0.97 | (0.86, 1.10) | 0.7003 |
| rs2021719 | 30557461 | 0.43 | 0.37 | 0.90 | (0.82, 1.00) | 0.0538 |
| rs3094694 | 30559883 | 0.23 | 0.20 | 1.35 | (1.20, 1.52) | 9.23E-07 |
| rs2157605 | 30562055 | 0.21 | 0.17 | 0.94 | (0.82, 1.07) | 0.3624 |
| rs1264459 | 30563899 | 0.12 | 0.16 | 0.97 | (0.85, 1.10) | 0.5999 |
| rs1059655 | 30569064 | 0.33 | 0.32 | 0.91 | (0.81, 1.01) | 0.0628 |
| rs1264456 | 30570063 | 0.33 | 0.31 | 0.91 | (0.82, 1.01) | 0.0868 |
| rs2508024 | 30573491 | 0.34 | 0.34 | 0.92 | (0.83, 1.02) | 0.1167 |
| rs3131115 | 30576770 | 0.37 | 0.34 | 1.26 | (1.12, 1.41) | 6.94E-05 |
| rs2844723 | 30578280 | 0.34 | 0.33 | 0.91 | (0.82, 1.02) | 0.0928 |
| rs2429657 | 30579499 | 0.22 | 0.24 | 0.88 | (0.78, 0.98) | 0.0243 |
| rs2516662 | 30583394 | 0.34 | 0.33 | 0.93 | (0.84, 1.03) | 0.1555 |
| rs975195 | 30585926 | 0.34 | 0.34 | 0.89 | (0.80, 0.99) | 0.0247 |
| rs2534814 | 30588856 | 0.33 | 0.32 | 0.92 | (0.83, 1.02) | 0.1126 |
| rs2534804 | 30594435 | 0.33 | 0.30 | 0.90 | (0.80, 1.00) | 0.0456 |
| rs7767714 | 30596012 | 0.00 | 0.01 | 1.18 | (0.77, 1.81) | 0.4522 |
| rs2534825 | 30603722 | 0.34 | 0.33 | 0.92 | (0.83, 1.02) | 0.1305 |
| rs2534816 | 30606843 | 0.14 | 0.14 | 0.93 | (0.81, 1.07) | 0.3132 |
| rs1362119 | 30607711 | 0.34 | 0.34 | 0.95 | (0.85, 1.05) | 0.3216 |
| rs1468079 | 30614362 | 0.34 | 0.34 | 0.92 | (0.83, 1.02) | 0.1236 |
| rs2524222 | 30619149 | 0.22 | 0.22 | 0.86 | (0.76, 0.97) | 0.0105 |
| rs1058318 | 30620142 | 0.33 | 0.31 | 0.93 | (0.84, 1.04) | 0.1968 |
| rs2844714 | 30626598 | 0.34 | 0.34 | 0.93 | (0.84, 1.03) | 0.1586 |
| rs2074505 | 30629116 | 0.34 | 0.35 | 0.88 | (0.79, 0.97) | 0.0153 |
| rs2516647 | 30634824 | 0.34 | 0.36 | 0.92 | (0.83, 1.02) | 0.0998 |
| rs2516641 | 30636664 | 0.33 | 0.32 | 0.90 | (0.81, 1.00) | 0.0513 |
| rs2074503 | 30638475 | 0.33 | 0.31 | 0.91 | (0.82, 1.01) | 0.0864 |
| rs9262113 | 30640257 | 0.13 | 0.15 | 0.94 | (0.82, 1.08) | 0.4093 |
| rs3130041 | 30644112 | 0.34 | 0.33 | 0.92 | (0.83, 1.02) | 0.1262 |
| rs2269710 | 30647931 | 0.34 | 0.34 | 0.92 | (0.83, 1.03) | 0.1333 |
| rs3132610 | 30652380 | 0.15 | 0.13 | 1.47 | (1.27, 1.69) | 9.34E-08 |
| rs1264440 | 30659265 | 0.34 | 0.32 | 0.91 | (0.82, 1.01) | 0.0864 |
| rs7749109 | 30669703 | 0.00 | 0.02 | 1.09 | (0.76, 1.56) | 0.6469 |
| rs3130245 | 30672322 | 0.08 | 0.09 | 0.92 | (0.77, 1.09) | 0.3368 |
| rs1264423 | 30679450 | 0.46 | 0.46 | 0.91 | (0.82, 1.00) | 0.0455 |
| rs1264420 | 30683582 | 0.31 | 0.28 | 0.95 | (0.85, 1.06) | 0.3648 |
| rs1264419 | 30684760 | 0.46 | 0.46 | 0.87 | (0.79, 0.96) | 0.0051 |
| rs2267637 | 30686314 | 0.08 | 0.11 | 0.97 | (0.83, 1.14) | 0.7256 |
| rs2267640 | 30688872 | 0.00 | 0.02 | 1.02 | (0.69, 1.50) | 0.9227 |
| rs2270172 | 30700393 | 0.01 | 0.02 | 1.09 | (0.78, 1.52) | 0.6105 |
| rs4713340 | 30707681 | 0.13 | 0.15 | 0.92 | (0.81, 1.06) | 0.2586 |
| rs12665339 | 30709211 | 0.23 | 0.16 | 0.90 | (0.78, 1.03) | 0.1279 |
| rs2394390 | 30709800 | 0.02 | 0.02 | 1.30 | (0.94, 1.80) | 0.1168 |
| rs3094104 | 30717608 | 0.02 | 0.04 | 1.07 | (0.85, 1.36) | 0.5525 |
| rs1140809 | 30719655 | 0.46 | 0.47 | 0.89 | (0.80, 0.98) | 0.0146 |
| rs17281677 | 30730438 | 0.03 | 0.03 | 1.11 | (0.83, 1.50) | 0.4777 |
| rs9262138 | 30735846 | 0.09 | 0.07 | 0.90 | (0.74, 1.09) | 0.2865 |
| rs3094097 | 30741854 | 0.02 | 0.06 | 1.03 | (0.84, 1.26) | 0.7555 |
| rs3129996 | 30759566 | 0.08 | 0.09 | 0.93 | (0.78, 1.10) | 0.3756 |
| rs1075496 | 30766218 | 0.38 | 0.46 | 1.08 | (0.97, 1.19) | 0.1500 |
| rs6929626 | 30787312 | 0.02 | 0.02 | 0.83 | (0.58, 1.18) | 0.2983 |
| rs3094093 | 30787607 | 0.07 | 0.06 | 0.97 | (0.80, 1.19) | 0.7980 |
| rs9262152 | 30788895 | 0.13 | 0.15 | 0.92 | (0.80, 1.06) | 0.2344 |
| rs8233 | 30800944 | 0.19 | 0.24 | 1.24 | (1.10, 1.38) | 0.0003 |
| rs3094127 | 30805426 | 0.20 | 0.24 | 1.32 | (1.17, 1.48) | 2.68E-06 |
| rs9501029 | 30805719 | 0.03 | 0.05 | 0.95 | (0.75, 1.19) | 0.6481 |
| rs1064627 | 30806520 | 0.19 | 0.22 | 1.18 | (1.04, 1.33) | 0.0079 |
| rs9968884 | 30808674 | 0.03 | 0.04 | 0.93 | (0.72, 1.21) | 0.6023 |
| rs2535320 | 30812962 | 0.07 | 0.04 | 1.04 | (0.80, 1.35) | 0.7681 |
| rs1059612 | 30816934 | 0.16 | 0.13 | 1.54 | (1.34, 1.77) | 1.55E-09 |
| rs8512 | 30819336 | 0.15 | 0.19 | 0.83 | (0.73, 0.94) | 0.0028 |
| rs2284174 | 30821559 | 0.18 | 0.22 | 1.30 | (1.15, 1.46) | 1.30E-05 |
| rs4713358 | 30824096 | 0.00 | 0.03 | 0.93 | (0.69, 1.25) | 0.6255 |
| rs2535324 | 30826014 | 0.32 | 0.36 | 1.11 | (1.00, 1.23) | 0.0447 |
| rs3129973 | 30829122 | 0.17 | 0.14 | 1.49 | (1.30, 1.70) | 6.09E-09 |
| rs2394401 | 30832409 | 0.10 | 0.11 | 0.92 | (0.79, 1.07) | 0.2865 |
| rs3131036 | 30836269 | 0.28 | 0.28 | 1.20 | (1.08, 1.34) | 0.0008 |
| rs3095338 | 30839380 | 0.18 | 0.17 | 1.38 | (1.22, 1.57) | 5.62E-07 |
| rs3131038 | 30842059 | 0.26 | 0.25 | 1.24 | (1.11, 1.39) | 0.0001 |
| rs12526186 | 30844130 | 0.13 | 0.18 | 0.83 | (0.73, 0.95) | 0.0062 |
| rs3094116 | 30846387 | 0.28 | 0.29 | 1.23 | (1.11, 1.37) | 0.0001 |
| rs3130666 | 30848139 | 0.03 | 0.03 | 1.04 | (0.79, 1.37) | 0.7825 |
| rs4248148 | 30850113 | 0.09 | 0.11 | 0.86 | (0.74, 1.01) | 0.0735 |
| rs3130667 | 30851220 | 0.38 | 0.34 | 0.89 | (0.81, 0.99) | 0.0328 |
| rs3130673 | 30854498 | 0.18 | 0.14 | 1.44 | (1.25, 1.65) | 1.77E-07 |
| rs9468830 | 30857691 | 0.23 | 0.31 | 0.86 | (0.77, 0.96) | 0.0060 |
| rs7382989 | 30861125 | 0.23 | 0.31 | 0.85 | (0.77, 0.95) | 0.0035 |
| rs12527415 | 30862519 | 0.23 | 0.31 | 0.84 | (0.75, 0.93) | 0.0012 |
| rs3131043 | 30866445 | 0.42 | 0.47 | 1.05 | (0.95, 1.16) | 0.2937 |
| rs3131064 | 30871872 | 0.20 | 0.19 | 1.38 | (1.22, 1.56) | 2.18E-07 |
| rs1264375 | 30874190 | 0.28 | 0.31 | 1.13 | (1.02, 1.25) | 0.0210 |
| rs3129988 | 30877457 | 0.08 | 0.06 | 1.02 | (0.82, 1.27) | 0.8461 |
| rs4713372 | 30878757 | 0.27 | 0.30 | 0.85 | (0.76, 0.95) | 0.0031 |
| rs4711240 | 30881218 | 0.17 | 0.14 | 1.02 | (0.89, 1.18) | 0.7635 |
| rs1264362 | 30884569 | 0.34 | 0.28 | 0.95 | (0.85, 1.06) | 0.3220 |
| rs12200562 | 30884600 | 0.16 | 0.12 | 1.05 | (0.90, 1.22) | 0.5476 |
| rs9380197 | 30886182 | 0.27 | 0.31 | 0.88 | (0.79, 0.98) | 0.0258 |
| rs4327730 | 30888915 | 0.16 | 0.12 | 1.04 | (0.90, 1.21) | 0.5696 |
| rs2394412 | 30890214 | 0.27 | 0.30 | 0.87 | (0.78, 0.97) | 0.0124 |
| rs9380198 | 30893865 | 0.10 | 0.16 | 0.80 | (0.70, 0.91) | 0.0010 |
| rs9348843 | 30894655 | 0.27 | 0.29 | 0.89 | (0.80, 1.00) | 0.0417 |
| rs4713382 | 30895154 | 0.10 | 0.16 | 0.80 | (0.70, 0.92) | 0.0011 |
| rs4713383 | 30895220 | 0.10 | 0.16 | 0.78 | (0.68, 0.89) | 0.0003 |
| rs3094111 | 30896170 | 0.18 | 0.19 | 0.90 | (0.80, 1.03) | 0.1141 |
| rs1264352 | 30897626 | 0.19 | 0.18 | 1.42 | (1.25, 1.60) | 2.26E-08 |
| rs4713389 | 30898583 | 0.10 | 0.16 | 0.80 | (0.70, 0.92) | 0.0016 |
| rs4713391 | 30900214 | 0.18 | 0.14 | 1.03 | (0.90, 1.19) | 0.6623 |
| rs12192828 | 30900471 | 0.17 | 0.12 | 1.06 | (0.92, 1.24) | 0.4214 |
| rs7751869 | 30901293 | 0.10 | 0.17 | 0.80 | (0.70, 0.92) | 0.0012 |
| rs915664 | 30902596 | 0.33 | 0.28 | 0.96 | (0.86, 1.07) | 0.4023 |
| rs3130785 | 30904717 | 0.18 | 0.17 | 0.97 | (0.85, 1.11) | 0.6924 |
| rs3094110 | 30907022 | 0.38 | 0.39 | 0.88 | (0.80, 0.97) | 0.0141 |
| rs1264344 | 30908556 | 0.43 | 0.44 | 0.92 | (0.84, 1.02) | 0.1157 |
| rs1264341 | 30910444 | 0.18 | 0.14 | 1.53 | (1.34, 1.75) | 4.10E-10 |
| rs3130649 | 30911233 | 0.42 | 0.40 | 0.87 | (0.79, 0.96) | 0.0080 |
| rs3095352 | 30913900 | 0.42 | 0.42 | 0.83 | (0.76, 0.92) | 0.0003 |
| rs3130787 | 30917843 | 0.39 | 0.38 | 0.84 | (0.76, 0.93) | 0.0008 |
| rs2535335 | 30920476 | 0.34 | 0.38 | 0.95 | (0.86, 1.05) | 0.3017 |
| rs2250264 | 30929166 | 0.23 | 0.23 | 0.93 | (0.82, 1.04) | 0.1839 |
| rs7772269 | 30933199 | 0.08 | 0.07 | 0.96 | (0.80, 1.16) | 0.6625 |
| rs2844657 | 30937501 | 0.26 | 0.22 | 1.37 | (1.22, 1.54) | 8.08E-08 |
| rs7761138 | 30938271 | 0.08 | 0.07 | 0.99 | (0.82, 1.19) | 0.8831 |
| rs3130791 | 30939822 | 0.40 | 0.41 | 0.84 | (0.76, 0.93) | 0.0005 |
| rs3095354 | 30944090 | 0.40 | 0.41 | 0.84 | (0.76, 0.93) | 0.0006 |
| rs2844654 | 30946667 | 0.32 | 0.34 | 0.97 | (0.88, 1.07) | 0.5510 |
| rs3130796 | 30948929 | 0.40 | 0.40 | 0.84 | (0.76, 0.93) | 0.0009 |
| rs1264331 | 30954809 | 0.32 | 0.34 | 0.96 | (0.87, 1.06) | 0.4476 |
| rs9295930 | 30957801 | 0.16 | 0.14 | 0.87 | (0.75, 1.01) | 0.0681 |
| rs1264323 | 30963886 | 0.43 | 0.39 | 1.15 | (1.04, 1.28) | 0.0088 |
| rs2229933 | 30965051 | 0.17 | 0.18 | 0.83 | (0.73, 0.95) | 0.0051 |
| rs3132572 | 30969708 | 0.13 | 0.11 | 0.92 | (0.78, 1.07) | 0.2766 |
| rs886422 | 30972258 | 0.18 | 0.14 | 1.54 | (1.35, 1.76) | 1.92E-10 |
| rs1049623 | 30972808 | 0.43 | 0.42 | 1.09 | (0.98, 1.20) | 0.1069 |
| rs2239518 | 30973704 | 0.16 | 0.15 | 0.87 | (0.75, 1.00) | 0.0441 |
| rs2894055 | 30976607 | 0.17 | 0.17 | 0.85 | (0.74, 0.98) | 0.0213 |
| rs9468846 | 30978742 | 0.17 | 0.18 | 0.83 | (0.73, 0.95) | 0.0058 |
| rs2894054 | 30980253 | 0.08 | 0.08 | 0.96 | (0.80, 1.15) | 0.6543 |
| rs3130780 | 30982287 | 0.23 | 0.22 | 0.91 | (0.80, 1.02) | 0.1072 |
| rs2074508 | 30984417 | 0.17 | 0.18 | 0.84 | (0.74, 0.95) | 0.0064 |
| rs2074512 | 30986898 | 0.17 | 0.18 | 0.84 | (0.74, 0.95) | 0.0067 |
| rs1264308 | 30987966 | 0.18 | 0.14 | 1.55 | (1.36, 1.78) | 2.30E-10 |
| rs1264307 | 30988736 | 0.32 | 0.32 | 0.97 | (0.87, 1.08) | 0.5216 |
| rs1264300 | 30990835 | 0.32 | 0.33 | 0.99 | (0.89, 1.10) | 0.8428 |
| rs1264299 | 30991899 | 0.32 | 0.35 | 0.95 | (0.86, 1.06) | 0.3578 |
| rs7756286 | 30995290 | 0.32 | 0.34 | 0.96 | (0.87, 1.06) | 0.4372 |
| rs2249464 | 30996140 | 0.48 | 0.50 | 0.90 | (0.81, 0.99) | 0.0266 |
| rs753725 | 30998850 | 0.48 | 0.50 | 0.88 | (0.80, 0.97) | 0.0102 |
| rs9394021 | 31001106 | 0.17 | 0.16 | 0.85 | (0.73, 0.97) | 0.0181 |
| rs2532934 | 31002738 | 0.48 | 0.50 | 0.89 | (0.81, 0.98) | 0.0204 |
| rs3873332 | 31003969 | 0.16 | 0.15 | 0.85 | (0.74, 0.97) | 0.0196 |
| rs2517459 | 31005001 | 0.25 | 0.25 | 0.83 | (0.74, 0.94) | 0.0022 |
| rs2532929 | 31005753 | 0.34 | 0.37 | 0.94 | (0.85, 1.04) | 0.2382 |
| rs2253705 | 31008073 | 0.25 | 0.23 | 0.89 | (0.80, 1.01) | 0.0607 |
| rs2844650 | 31010512 | 0.13 | 0.11 | 0.90 | (0.77, 1.06) | 0.2063 |
| rs3132571 | 31013292 | 0.38 | 0.39 | 1.16 | (1.05, 1.28) | 0.0029 |
| rs6933400 | 31015155 | 0.12 | 0.12 | 0.88 | (0.75, 1.02) | 0.0952 |
| rs6933909 | 31015245 | 0.12 | 0.12 | 0.90 | (0.78, 1.05) | 0.1839 |
| rs2844702 | 31020460 | 0.32 | 0.35 | 0.96 | (0.87, 1.06) | 0.4204 |
| rs2517451 | 31022730 | 0.13 | 0.11 | 0.92 | (0.79, 1.08) | 0.3051 |
| rs3130782 | 31022822 | 0.18 | 0.17 | 1.33 | (1.17, 1.51) | 1.01E-05 |
| rs2517449 | 31027680 | 0.32 | 0.35 | 0.96 | (0.87, 1.07) | 0.4808 |
| rs3132580 | 31028103 | 0.18 | 0.17 | 1.29 | (1.14, 1.47) | 9.02E-05 |
| rs2240804 | 31028869 | 0.32 | 0.33 | 0.97 | (0.87, 1.07) | 0.5106 |
| rs2240803 | 31028936 | 0.18 | 0.16 | 0.85 | (0.74, 0.97) | 0.0183 |
| rs3757340 | 31029861 | 0.28 | 0.27 | 0.85 | (0.76, 0.95) | 0.0044 |
| rs12212418 | 31032003 | 0.28 | 0.27 | 0.86 | (0.77, 0.96) | 0.0066 |
| rs11753326 | 31033964 | 0.09 | 0.10 | 0.92 | (0.78, 1.09) | 0.3187 |
| rs3131934 | 31039823 | 0.19 | 0.16 | 1.43 | (1.25, 1.63) | 1.40E-07 |
| rs3132579 | 31048968 | 0.21 | 0.19 | 1.45 | (1.28, 1.63) | 1.75E-09 |
| rs3131931 | 31053244 | 0.27 | 0.29 | 1.34 | (1.20, 1.48) | 5.89E-08 |
| rs2844682 | 31054127 | 0.11 | 0.13 | 0.92 | (0.79, 1.06) | 0.2337 |
| rs2844680 | 31054475 | 0.40 | 0.41 | 0.88 | (0.80, 0.98) | 0.0130 |
| rs2844679 | 31055344 | 0.11 | 0.13 | 0.90 | (0.78, 1.04) | 0.1381 |
| rs2517424 | 31057975 | 0.11 | 0.13 | 0.92 | (0.80, 1.07) | 0.2733 |
| rs2530699 | 31061219 | 0.11 | 0.13 | 0.88 | (0.76, 1.02) | 0.0944 |
| rs2844677 | 31063338 | 0.05 | 0.06 | 0.94 | (0.76, 1.16) | 0.5601 |
| rs1634731 | 31063660 | 0.23 | 0.19 | 0.91 | (0.80, 1.04) | 0.1521 |
| rs2517416 | 31063908 | 0.11 | 0.13 | 0.94 | (0.81, 1.09) | 0.4014 |
| rs3873342 | 31065745 | 0.08 | 0.09 | 0.95 | (0.80, 1.13) | 0.5717 |
| rs2249168 | 31066233 | 0.11 | 0.13 | 0.92 | (0.80, 1.06) | 0.2352 |
| rs2530695 | 31066473 | 0.11 | 0.13 | 0.92 | (0.80, 1.06) | 0.2477 |
| rs2517411 | 31068246 | 0.11 | 0.13 | 0.93 | (0.81, 1.07) | 0.3054 |
| rs2844673 | 31069905 | 0.11 | 0.13 | 0.93 | (0.80, 1.08) | 0.3383 |
| rs2252926 | 31074283 | 0.11 | 0.14 | 0.89 | (0.77, 1.02) | 0.0998 |
| rs2429295 | 31077477 | 0.11 | 0.13 | 0.92 | (0.80, 1.06) | 0.2551 |
| rs1632856 | 31079715 | 0.28 | 0.33 | 1.27 | (1.15, 1.41) | 5.59E-06 |
| rs1634717 | 31080568 | 0.39 | 0.46 | 1.22 | (1.10, 1.34) | 6.13E-05 |
| rs1632854 | 31083628 | 0.39 | 0.46 | 1.22 | (1.11, 1.34) | 5.14E-05 |
| rs1634722 | 31087063 | 0.25 | 0.25 | 0.93 | (0.83, 1.04) | 0.1856 |
| rs1634725 | 31089987 | 0.28 | 0.32 | 1.31 | (1.18, 1.46) | 2.15E-07 |
| rs7755802 | 31090188 | 0.42 | 0.39 | 0.86 | (0.77, 0.95) | 0.0019 |
| rs1619376 | 31091305 | 0.25 | 0.23 | 0.92 | (0.82, 1.03) | 0.1466 |
| rs3871466 | 31091662 | 0.19 | 0.12 | 0.80 | (0.69, 0.94) | 0.0056 |
| rs4713411 | 31095155 | 0.42 | 0.39 | 0.84 | (0.76, 0.93) | 0.0007 |
| rs9262549 | 31105671 | 0.40 | 0.32 | 1.24 | (1.12, 1.37) | 4.89E-05 |
| rs6457300 | 31106721 | 0.24 | 0.33 | 0.90 | (0.81, 1.00) | 0.0525 |
| rs4713422 | 31107881 | 0.28 | 0.34 | 0.92 | (0.83, 1.02) | 0.1206 |
| rs4248154 | 31110595 | 0.13 | 0.16 | 0.90 | (0.79, 1.02) | 0.1055 |
| rs2523870 | 31122095 | 0.37 | 0.36 | 0.93 | (0.84, 1.02) | 0.1340 |
| rs3873352 | 31130092 | 0.09 | 0.09 | 1.00 | (0.85, 1.18) | 0.9804 |
| rs2523849 | 31133030 | 0.13 | 0.15 | 0.96 | (0.84, 1.10) | 0.5333 |
| rs9262636 | 31133827 | 0.22 | 0.21 | 0.97 | (0.86, 1.09) | 0.5584 |
| rs2517521 | 31135315 | 0.20 | 0.13 | 0.95 | (0.82, 1.10) | 0.4694 |
| rs2517512 | 31137664 | 0.08 | 0.12 | 0.98 | (0.84, 1.14) | 0.8084 |
| rs2523841 | 31138262 | 0.13 | 0.15 | 0.95 | (0.83, 1.09) | 0.4977 |
| rs2394423 | 31140021 | 0.22 | 0.21 | 0.98 | (0.87, 1.10) | 0.6791 |
| rs2517500 | 31141488 | 0.35 | 0.37 | 0.94 | (0.85, 1.04) | 0.2070 |
| rs2517497 | 31144762 | 0.35 | 0.37 | 0.95 | (0.86, 1.06) | 0.3642 |
| rs2523890 | 31147511 | 0.34 | 0.38 | 0.96 | (0.86, 1.06) | 0.3956 |
| rs2523882 | 31150196 | 0.23 | 0.28 | 1.02 | (0.91, 1.14) | 0.7172 |
| rs2249231 | 31151211 | 0.35 | 0.34 | 1.00 | (0.90, 1.12) | 0.9537 |
| rs2523843 | 31154507 | 0.35 | 0.37 | 0.95 | (0.86, 1.05) | 0.2731 |
| rs9380215 | 31157634 | 0.07 | 0.06 | 0.97 | (0.79, 1.19) | 0.7548 |
| rs2535311 | 31160663 | 0.35 | 0.37 | 0.94 | (0.85, 1.04) | 0.1961 |
| rs3130955 | 31162490 | 0.38 | 0.39 | 1.09 | (0.98, 1.20) | 0.1042 |
| rs4947296 | 31166157 | 0.07 | 0.06 | 0.94 | (0.76, 1.16) | 0.5752 |
| rs2517450 | 31169418 | 0.48 | 0.48 | 0.82 | (0.74, 0.90) | 7.11E-05 |
| rs2517423 | 31172806 | 0.48 | 0.48 | 0.81 | (0.74, 0.90) | 2.96E-05 |
| rs7381697 | 31176005 | 0.21 | 0.15 | 0.88 | (0.77, 1.01) | 0.0696 |
| rs7381897 | 31181882 | 0.48 | 0.48 | 0.82 | (0.74, 0.91) | 0.0001 |
| rs6457327 | 31182009 | 0.38 | 0.37 | 0.89 | (0.80, 0.98) | 0.0200 |
| rs1064190 | 31183094 | 0.48 | 0.48 | 0.82 | (0.74, 0.90) | 5.99E-05 |
| rs2517529 | 31184957 | 0.44 | 0.46 | 1.25 | (1.14, 1.38) | 7.06E-06 |
| rs1265063 | 31185917 | 0.48 | 0.47 | 0.82 | (0.75, 0.91) | 0.0001 |
| rs1265059 | 31186474 | 0.48 | 0.48 | 0.82 | (0.74, 0.90) | 4.52E-05 |
| rs3132556 | 31186788 | 0.21 | 0.21 | 1.45 | (1.27, 1.64) | 1.08E-08 |
| rs2233986 | 31187055 | 0.31 | 0.37 | 1.32 | (1.19, 1.47) | 2.72E-07 |
| rs2233969 | 31188411 | 0.21 | 0.15 | 0.90 | (0.78, 1.04) | 0.1406 |
| rs2270190 | 31188565 | 0.14 | 0.10 | 0.85 | (0.72, 1.00) | 0.0489 |
| rs2233965 | 31188878 | 0.20 | 0.14 | 0.88 | (0.77, 1.02) | 0.0813 |
| rs2233956 | 31189184 | 0.24 | 0.20 | 1.44 | (1.28, 1.62) | 2.30E-09 |
| rs1265048 | 31189388 | 0.25 | 0.31 | 1.01 | (0.91, 1.12) | 0.8008 |
| rs3823402 | 31189722 | 0.19 | 0.17 | 0.90 | (0.79, 1.02) | 0.1086 |
| rs3130977 | 31189968 | 0.36 | 0.34 | 1.31 | (1.18, 1.45) | 2.56E-07 |
| rs3130553 | 31190264 | 0.48 | 0.49 | 0.81 | (0.73, 0.89) | 1.36E-05 |
| rs3130980 | 31190383 | 0.28 | 0.33 | 0.84 | (0.76, 0.93) | 0.0010 |
| rs3094216 | 31192027 | 0.30 | 0.25 | 1.29 | (1.15, 1.44) | 1.06E-05 |
| rs3130982 | 31192054 | 0.40 | 0.48 | 0.84 | (0.75, 0.92) | 0.0005 |
| rs1042127 | 31192149 | 0.15 | 0.16 | 0.96 | (0.84, 1.10) | 0.5512 |
| rs707913 | 31192766 | 0.14 | 0.20 | 0.89 | (0.79, 1.02) | 0.0855 |
| rs3132552 | 31193248 | 0.30 | 0.27 | 0.92 | (0.82, 1.03) | 0.1419 |
| rs3094214 | 31193361 | 0.40 | 0.49 | 0.84 | (0.76, 0.93) | 0.0006 |
| rs3132550 | 31194027 | 0.30 | 0.24 | 1.30 | (1.16, 1.45) | 4.86E-06 |
| rs3130989 | 31194356 | 0.45 | 0.41 | 1.21 | (1.09, 1.34) | 0.0005 |
| rs2284177 | 31197574 | 0.15 | 0.17 | 0.96 | (0.84, 1.09) | 0.5158 |
| rs3095314 | 31197610 | 0.45 | 0.41 | 1.21 | (1.10, 1.33) | 0.0002 |
| rs3095312 | 31198816 | 0.28 | 0.35 | 0.86 | (0.77, 0.95) | 0.0045 |
| rs3131000 | 31199252 | 0.28 | 0.35 | 0.86 | (0.78, 0.96) | 0.0053 |
| rs3094207 | 31199388 | 0.25 | 0.31 | 0.84 | (0.75, 0.93) | 0.0010 |
| rs3094204 | 31199971 | 0.41 | 0.49 | 0.74 | (0.66, 0.82) | 2.34E-08 |
| rs3095307 | 31200028 | 0.43 | 0.48 | 0.84 | (0.76, 0.93) | 0.0004 |
| rs3778638 | 31200103 | 0.18 | 0.18 | 0.94 | (0.83, 1.07) | 0.3565 |
| rs3094198 | 31201306 | 0.31 | 0.41 | 0.87 | (0.79, 0.96) | 0.0056 |
| rs3815087 | 31201566 | 0.17 | 0.20 | 0.83 | (0.73, 0.95) | 0.0075 |
| rs3778639 | 31201755 | 0.11 | 0.11 | 0.75 | (0.64, 0.88) | 0.0004 |
| rs3778640 | 31202099 | 0.11 | 0.09 | 0.85 | (0.71, 1.01) | 0.0653 |
| rs3130558 | 31205162 | 0.30 | 0.27 | 0.95 | (0.85, 1.06) | 0.3368 |
| rs3130559 | 31205280 | 0.14 | 0.21 | 0.90 | (0.79, 1.01) | 0.0743 |
| rs3130564 | 31209653 | 0.28 | 0.22 | 1.32 | (1.18, 1.48) | 3.28E-06 |
| rs1265100 | 31213289 | 0.11 | 0.15 | 0.97 | (0.85, 1.12) | 0.7038 |
| rs2074478 | 31213612 | 0.19 | 0.18 | 0.88 | (0.77, 1.00) | 0.0442 |
| rs3130573 | 31214247 | 0.33 | 0.36 | 1.21 | (1.10, 1.34) | 0.0001 |
| rs1265095 | 31214622 | 0.49 | 0.46 | 0.87 | (0.79, 0.96) | 0.0037 |
| rs3094663 | 31215066 | 0.33 | 0.30 | 0.88 | (0.79, 0.98) | 0.0220 |
| rs1063646 | 31215627 | 0.16 | 0.14 | 0.94 | (0.82, 1.09) | 0.4015 |
| rs3130454 | 31216464 | 0.33 | 0.29 | 0.87 | (0.78, 0.97) | 0.0122 |
| rs1265087 | 31217789 | 0.32 | 0.35 | 1.20 | (1.09, 1.33) | 0.0003 |
| rs130073 | 31219159 | 0.33 | 0.29 | 0.89 | (0.79, 0.99) | 0.0379 |
| rs1265080 | 31220054 | 0.49 | 0.49 | 0.84 | (0.76, 0.93) | 0.0011 |
| rs2073719 | 31220904 | 0.29 | 0.24 | 0.89 | (0.79, 1.00) | 0.0512 |
| rs3094225 | 31221031 | 0.33 | 0.26 | 0.88 | (0.79, 0.99) | 0.0277 |
| rs3132538 | 31221821 | 0.27 | 0.20 | 0.88 | (0.78, 0.99) | 0.0344 |
| rs746647 | 31222161 | 0.32 | 0.34 | 1.23 | (1.11, 1.36) | 8.94E-05 |
| rs2240064 | 31222552 | 0.48 | 0.44 | 0.88 | (0.80, 0.97) | 0.0119 |
| rs1265115 | 31225054 | 0.31 | 0.33 | 0.79 | (0.71, 0.88) | 1.88E-05 |
| rs1265114 | 31225167 | 0.32 | 0.35 | 1.20 | (1.09, 1.33) | 0.0004 |
| rs1265112 | 31225998 | 0.32 | 0.34 | 1.17 | (1.05, 1.30) | 0.0040 |
| rs2073717 | 31230105 | 0.45 | 0.38 | 0.90 | (0.81, 1.00) | 0.0486 |
| rs3130453 | 31232828 | 0.47 | 0.49 | 0.92 | (0.83, 1.01) | 0.0722 |
| rs743401 | 31233137 | 0.45 | 0.38 | 0.92 | (0.83, 1.01) | 0.0764 |
| rs3095239 | 31234769 | 0.50 | 0.46 | 0.93 | (0.84, 1.03) | 0.1426 |
| rs3094187 | 31234923 | 0.50 | 0.46 | 0.94 | (0.85, 1.04) | 0.2033 |
| rs1150765 | 31235541 | 0.27 | 0.27 | 1.20 | (1.07, 1.34) | 0.0012 |
| rs7750641 | 31237289 | 0.16 | 0.12 | 1.63 | (1.40, 1.90) | 3.95E-10 |
| rs2073724 | 31237686 | 0.05 | 0.09 | 0.96 | (0.81, 1.14) | 0.6659 |
| rs1065461 | 31238481 | 0.30 | 0.23 | 0.94 | (0.84, 1.06) | 0.3040 |
| rs1419881 | 31238572 | 0.47 | 0.49 | 1.08 | (0.98, 1.19) | 0.1313 |
| rs13409 | 31240119 | 0.43 | 0.46 | 1.10 | (1.00, 1.21) | 0.0584 |
| rs2394882 | 31240628 | 0.35 | 0.31 | 0.97 | (0.87, 1.08) | 0.5318 |
| rs2106074 | 31241488 | 0.35 | 0.32 | 0.97 | (0.88, 1.08) | 0.6196 |
| rs3130931 | 31242867 | 0.38 | 0.29 | 0.89 | (0.80, 0.99) | 0.0267 |
| rs3130502 | 31244645 | 0.30 | 0.24 | 0.93 | (0.83, 1.04) | 0.2173 |
| rs3130503 | 31245144 | 0.27 | 0.21 | 0.88 | (0.78, 0.99) | 0.0376 |
| rs885950 | 31248131 | 0.43 | 0.45 | 1.13 | (1.03, 1.25) | 0.0131 |
| rs1265158 | 31248720 | 0.41 | 0.38 | 1.12 | (1.01, 1.24) | 0.0249 |
| rs887468 | 31249502 | 0.30 | 0.31 | 1.19 | (1.08, 1.33) | 0.0009 |
| rs9468877 | 31250882 | 0.13 | 0.13 | 0.95 | (0.82, 1.09) | 0.4712 |
| rs3131018 | 31251561 | 0.42 | 0.36 | 0.88 | (0.79, 0.97) | 0.0133 |
| rs915660 | 31251814 | 0.13 | 0.18 | 0.88 | (0.77, 1.00) | 0.0504 |
| rs4947305 | 31252408 | 0.28 | 0.37 | 0.88 | (0.80, 0.97) | 0.0141 |
| rs3868542 | 31253818 | 0.28 | 0.36 | 0.89 | (0.81, 0.99) | 0.0352 |
| rs887464 | 31253899 | 0.40 | 0.46 | 1.14 | (1.03, 1.26) | 0.0075 |
| rs3871248 | 31253970 | 0.28 | 0.36 | 0.88 | (0.79, 0.97) | 0.0131 |
| rs1052989 | 31254382 | 0.13 | 0.19 | 0.88 | (0.77, 0.99) | 0.0380 |
| rs4713438 | 31254825 | 0.14 | 0.17 | 1.01 | (0.89, 1.15) | 0.8505 |
| rs3130457 | 31255173 | 0.27 | 0.26 | 1.22 | (1.10, 1.36) | 0.0003 |
| rs10947142 | 31258414 | 0.28 | 0.23 | 0.97 | (0.86, 1.09) | 0.5667 |
| rs6921663 | 31262599 | 0.16 | 0.24 | 0.87 | (0.78, 0.98) | 0.0223 |
| rs7759909 | 31266668 | 0.06 | 0.10 | 1.12 | (0.96, 1.30) | 0.1563 |
| rs4713447 | 31270942 | 0.30 | 0.41 | 0.93 | (0.84, 1.02) | 0.1270 |
| rs3094609 | 31273545 | 0.23 | 0.14 | 0.92 | (0.80, 1.07) | 0.2795 |
| rs3868075 | 31275794 | 0.30 | 0.41 | 0.93 | (0.84, 1.02) | 0.1321 |
| rs4122189 | 31275906 | 0.16 | 0.24 | 0.89 | (0.79, 0.99) | 0.0393 |
| rs9263875 | 31278893 | 0.35 | 0.37 | 0.83 | (0.74, 0.92) | 0.0004 |
| rs12205648 | 31281657 | 0.23 | 0.15 | 0.92 | (0.80, 1.06) | 0.2503 |
| rs7451190 | 31282506 | 0.41 | 0.46 | 0.90 | (0.82, 0.99) | 0.0282 |
| rs7451258 | 31282569 | 0.18 | 0.14 | 0.86 | (0.74, 0.99) | 0.0326 |
| rs7452908 | 31284900 | 0.22 | 0.18 | 0.87 | (0.76, 0.99) | 0.0387 |
| rs12195455 | 31286985 | 0.23 | 0.15 | 0.92 | (0.80, 1.06) | 0.2520 |
| rs3869109 | 31292175 | 0.46 | 0.46 | 1.09 | (0.99, 1.20) | 0.0857 |
| rs7383336 | 31295054 | 0.31 | 0.31 | 1.16 | (1.05, 1.29) | 0.0048 |
| rs12662501 | 31298829 | 0.13 | 0.14 | 1.00 | (0.87, 1.15) | 1.0000 |
| rs6904669 | 31300775 | 0.33 | 0.41 | 0.90 | (0.82, 1.00) | 0.0464 |
| rs3130521 | 31304355 | 0.43 | 0.38 | 0.90 | (0.81, 0.99) | 0.0340 |
| rs7745906 | 31311987 | 0.13 | 0.17 | 0.87 | (0.77, 0.99) | 0.0408 |
| rs3130685 | 31314185 | 0.45 | 0.45 | 1.17 | (1.06, 1.29) | 0.0015 |
| rs2894189 | 31325794 | 0.45 | 0.41 | 0.97 | (0.87, 1.07) | 0.4963 |
| rs3095254 | 31329647 | 0.38 | 0.35 | 0.98 | (0.88, 1.08) | 0.6818 |
| rs2894196 | 31338090 | 0.05 | 0.06 | 0.90 | (0.73, 1.10) | 0.3243 |
| rs7747738 | 31338903 | 0.03 | 0.03 | 1.01 | (0.76, 1.33) | 0.9707 |
| rs2853961 | 31339968 | 0.38 | 0.41 | 1.19 | (1.08, 1.31) | 0.0006 |
| rs3130542 | 31340090 | 0.28 | 0.21 | 0.87 | (0.77, 0.99) | 0.0274 |
| rs2844623 | 31340522 | 0.17 | 0.16 | 0.98 | (0.86, 1.13) | 0.7878 |
| rs2394953 | 31341332 | 0.28 | 0.22 | 0.88 | (0.78, 0.99) | 0.0282 |
| rs2524100 | 31343748 | 0.13 | 0.11 | 0.94 | (0.81, 1.11) | 0.4573 |
| rs2853950 | 31344154 | 0.39 | 0.42 | 0.92 | (0.83, 1.01) | 0.0819 |
| rs1049853 | 31344879 | 0.13 | 0.10 | 0.97 | (0.83, 1.14) | 0.6762 |
| rs2074489 | 31348107 | 0.16 | 0.23 | 0.94 | (0.83, 1.05) | 0.2784 |
| rs4361609 | 31348614 | 0.08 | 0.13 | 1.02 | (0.88, 1.18) | 0.8005 |
| rs7759127 | 31348967 | 0.13 | 0.11 | 0.96 | (0.82, 1.11) | 0.5354 |
| rs2844621 | 31349516 | 0.38 | 0.27 | 0.95 | (0.85, 1.06) | 0.3049 |
| rs3132486 | 31351149 | 0.49 | 0.47 | 0.95 | (0.86, 1.04) | 0.2540 |
| rs2524069 | 31352768 | 0.18 | 0.17 | 1.38 | (1.21, 1.58) | 1.19E-06 |
| rs6906846 | 31353715 | 0.33 | 0.29 | 1.03 | (0.93, 1.15) | 0.5779 |
| rs7381988 | 31354682 | 0.26 | 0.17 | 0.91 | (0.79, 1.03) | 0.1365 |
| rs7382297 | 31355046 | 0.23 | 0.15 | 0.92 | (0.81, 1.06) | 0.2555 |
| rs2853939 | 31358621 | 0.48 | 0.43 | 1.11 | (1.00, 1.22) | 0.0446 |
| rs12191877 | 31360904 | 0.13 | 0.14 | 0.86 | (0.74, 0.99) | 0.0336 |
| rs2853933 | 31362067 | 0.48 | 0.44 | 1.11 | (1.01, 1.23) | 0.0337 |
| rs2524043 | 31364991 | 0.13 | 0.11 | 0.97 | (0.84, 1.13) | 0.6768 |
| rs2524156 | 31368376 | 0.48 | 0.44 | 1.13 | (1.02, 1.24) | 0.0194 |
| rs3906272 | 31370903 | 0.13 | 0.14 | 0.79 | (0.69, 0.91) | 0.0014 |
| rs2853926 | 31371030 | 0.27 | 0.27 | 0.90 | (0.81, 1.00) | 0.0537 |
| rs2894207 | 31371730 | 0.19 | 0.19 | 0.85 | (0.75, 0.96) | 0.0110 |
| rs3905495 | 31373518 | 0.28 | 0.32 | 0.90 | (0.81, 1.01) | 0.0705 |
| rs7760988 | 31381009 | 0.08 | 0.09 | 0.77 | (0.64, 0.93) | 0.0051 |
| rs3873386 | 31381724 | 0.27 | 0.36 | 0.97 | (0.88, 1.07) | 0.5565 |
| rs2854008 | 31420517 | 0.30 | 0.26 | 1.34 | (1.19, 1.50) | 5.34E-07 |
| rs7453967 | 31422222 | 0.25 | 0.17 | 0.96 | (0.84, 1.10) | 0.6079 |
| rs2923006 | 31425078 | 0.50 | 0.47 | 0.85 | (0.77, 0.94) | 0.0022 |
| rs4540292 | 31425161 | 0.34 | 0.38 | 0.92 | (0.84, 1.02) | 0.1045 |
| rs2156875 | 31425326 | 0.50 | 0.47 | 0.85 | (0.77, 0.94) | 0.0013 |
| rs2844586 | 31426003 | 0.05 | 0.05 | 1.01 | (0.80, 1.27) | 0.9539 |
| rs2523619 | 31426123 | 0.19 | 0.21 | 0.85 | (0.75, 0.97) | 0.0114 |
| rs2442728 | 31427334 | 0.40 | 0.40 | 1.10 | (0.99, 1.22) | 0.0725 |
| rs2596503 | 31428789 | 0.26 | 0.19 | 0.95 | (0.84, 1.09) | 0.4762 |
| rs1058026 | 31429664 | 0.13 | 0.14 | 0.92 | (0.80, 1.06) | 0.2674 |
| rs3819294 | 31430466 | 0.08 | 0.09 | 0.75 | (0.62, 0.89) | 0.0012 |
| rs2523589 | 31435313 | 0.47 | 0.48 | 0.83 | (0.76, 0.92) | 0.0003 |
| rs2523567 | 31437994 | 0.24 | 0.25 | 1.34 | (1.20, 1.49) | 2.67E-07 |
| rs2844580 | 31441282 | 0.43 | 0.41 | 1.12 | (1.01, 1.24) | 0.0301 |
| rs2596574 | 31442153 | 0.07 | 0.07 | 1.00 | (0.82, 1.21) | 0.9751 |
| rs2844575 | 31442924 | 0.46 | 0.45 | 0.83 | (0.75, 0.92) | 0.0003 |
| rs2844573 | 31443433 | 0.37 | 0.32 | 1.20 | (1.08, 1.34) | 0.0009 |
| rs6936035 | 31449135 | 0.13 | 0.20 | 0.99 | (0.88, 1.12) | 0.8703 |
| rs6933050 | 31451611 | 0.18 | 0.20 | 0.85 | (0.75, 0.96) | 0.0091 |
| rs2844535 | 31458282 | 0.26 | 0.28 | 1.23 | (1.11, 1.37) | 0.0001 |
| rs2844533 | 31458781 | 0.29 | 0.26 | 0.95 | (0.85, 1.06) | 0.3532 |
| rs2523480 | 31463681 | 0.21 | 0.13 | 0.94 | (0.81, 1.10) | 0.4442 |
| rs9266791 | 31466922 | 0.38 | 0.46 | 0.89 | (0.81, 0.98) | 0.0202 |
| rs2523459 | 31473287 | 0.24 | 0.30 | 0.94 | (0.85, 1.05) | 0.2793 |
| rs2523454 | 31475844 | 0.37 | 0.33 | 1.09 | (0.98, 1.21) | 0.1257 |
| rs2523452 | 31476519 | 0.24 | 0.32 | 0.93 | (0.83, 1.03) | 0.1571 |
| rs2844521 | 31476943 | 0.24 | 0.31 | 0.95 | (0.86, 1.06) | 0.3592 |
| rs3763288 | 31478346 | 0.07 | 0.09 | 0.65 | (0.54, 0.79) | 1.27E-05 |
| rs2256328 | 31489616 | 0.13 | 0.13 | 1.07 | (0.92, 1.23) | 0.3765 |
| rs2848713 | 31492458 | 0.06 | 0.05 | 0.97 | (0.78, 1.20) | 0.7641 |
| rs2596530 | 31495352 | 0.44 | 0.48 | 1.13 | (1.03, 1.25) | 0.0130 |
| rs2848716 | 31495946 | 0.09 | 0.12 | 0.84 | (0.72, 0.98) | 0.0284 |
| rs2844513 | 31496193 | 0.35 | 0.41 | 0.93 | (0.84, 1.03) | 0.1879 |
| rs2516448 | 31498389 | 0.44 | 0.47 | 1.15 | (1.04, 1.27) | 0.0076 |
| rs2524279 | 31500885 | 0.07 | 0.08 | 0.80 | (0.65, 0.97) | 0.0237 |
| rs3093960 | 31511497 | 0.21 | 0.14 | 0.95 | (0.83, 1.10) | 0.5001 |
| rs2516470 | 31515310 | 0.37 | 0.33 | 0.96 | (0.86, 1.06) | 0.4126 |
| rs2523693 | 31526103 | 0.49 | 0.49 | 0.84 | (0.76, 0.93) | 0.0005 |
| rs2516459 | 31527021 | 0.49 | 0.48 | 0.85 | (0.78, 0.94) | 0.0018 |
| rs3131622 | 31528479 | 0.43 | 0.43 | 1.18 | (1.07, 1.30) | 0.0012 |
| rs3131621 | 31533478 | 0.36 | 0.32 | 1.22 | (1.10, 1.36) | 0.0003 |
| rs2596480 | 31533964 | 0.06 | 0.07 | 1.08 | (0.89, 1.31) | 0.4223 |
| rs2523685 | 31534235 | 0.17 | 0.20 | 0.97 | (0.86, 1.10) | 0.6649 |
| rs2254618 | 31535118 | 0.19 | 0.24 | 1.00 | (0.89, 1.11) | 0.9322 |
| rs2596472 | 31536946 | 0.28 | 0.26 | 0.96 | (0.86, 1.07) | 0.4510 |
| rs3094228 | 31537906 | 0.21 | 0.24 | 1.46 | (1.30, 1.64) | 9.94E-11 |
| rs3099840 | 31538700 | 0.18 | 0.23 | 1.39 | (1.24, 1.56) | 1.89E-08 |
| rs2596473 | 31538778 | 0.43 | 0.43 | 0.91 | (0.82, 1.01) | 0.0625 |
| rs2395029 | 31539759 | 0.05 | 0.03 | 0.68 | (0.50, 0.91) | 0.0087 |
| rs2243621 | 31539799 | 0.17 | 0.16 | 0.89 | (0.78, 1.01) | 0.0805 |
| rs2284178 | 31540104 | 0.43 | 0.43 | 0.91 | (0.82, 1.00) | 0.0441 |
| rs3094014 | 31541537 | 0.18 | 0.21 | 1.39 | (1.24, 1.56) | 2.44E-08 |
| rs2244546 | 31543812 | 0.11 | 0.09 | 0.87 | (0.73, 1.04) | 0.1162 |
| rs2523676 | 31543970 | 0.21 | 0.17 | 0.82 | (0.72, 0.93) | 0.0022 |
| rs2859448 | 31544252 | 0.25 | 0.25 | 0.96 | (0.85, 1.07) | 0.4540 |
| rs2844508 | 31544478 | 0.36 | 0.31 | 0.96 | (0.87, 1.07) | 0.4844 |
| rs2523674 | 31544768 | 0.37 | 0.50 | 0.83 | (0.75, 0.91) | 0.0002 |
| rs2395031 | 31545284 | 0.08 | 0.04 | 1.26 | (0.98, 1.62) | 0.0724 |
| rs2523668 | 31546075 | 0.22 | 0.13 | 0.84 | (0.73, 0.97) | 0.0147 |
| rs2244839 | 31546347 | 0.18 | 0.25 | 0.95 | (0.85, 1.07) | 0.3897 |
| rs7758090 | 31546773 | 0.09 | 0.06 | 1.08 | (0.88, 1.33) | 0.4406 |
| rs1055569 | 31548061 | 0.27 | 0.31 | 0.98 | (0.88, 1.09) | 0.7447 |
| rs2516440 | 31548476 | 0.28 | 0.32 | 0.98 | (0.88, 1.09) | 0.6882 |
| rs3828886 | 31548531 | 0.23 | 0.25 | 0.98 | (0.87, 1.09) | 0.6645 |
| rs4413654 | 31549328 | 0.23 | 0.25 | 0.98 | (0.88, 1.10) | 0.7870 |
| rs6940467 | 31550116 | 0.13 | 0.09 | 1.00 | (0.84, 1.18) | 0.9833 |
| rs12660382 | 31551302 | 0.15 | 0.19 | 0.89 | (0.78, 1.01) | 0.0659 |
| rs2523651 | 31556133 | 0.44 | 0.35 | 0.91 | (0.82, 1.01) | 0.0781 |
| rs3749945 | 31556797 | 0.07 | 0.05 | 1.04 | (0.84, 1.28) | 0.7456 |
| rs2523647 | 31557757 | 0.22 | 0.23 | 0.90 | (0.80, 1.02) | 0.0888 |
| rs2516507 | 31559192 | 0.50 | 0.39 | 0.92 | (0.83, 1.03) | 0.1429 |
| rs2523705 | 31559659 | 0.13 | 0.20 | 0.88 | (0.77, 0.99) | 0.0337 |
| rs2516500 | 31561619 | 0.31 | 0.35 | 1.20 | (1.08, 1.33) | 0.0004 |
| rs3130914 | 31565365 | 0.38 | 0.40 | 1.23 | (1.11, 1.36) | 9.74E-05 |
| rs2516415 | 31567721 | 0.32 | 0.36 | 1.24 | (1.12, 1.37) | 3.37E-05 |
| rs3130922 | 31569068 | 0.41 | 0.32 | 0.94 | (0.85, 1.05) | 0.2616 |
| rs2516408 | 31571470 | 0.31 | 0.35 | 1.27 | (1.14, 1.41) | 5.34E-06 |
| rs2534679 | 31571769 | 0.31 | 0.35 | 1.27 | (1.14, 1.40) | 5.38E-06 |
| rs6916394 | 31572029 | 0.26 | 0.29 | 0.84 | (0.76, 0.94) | 0.0018 |
| rs3828903 | 31572718 | 0.26 | 0.30 | 0.78 | (0.70, 0.87) | 8.15E-06 |
| rs2534674 | 31573082 | 0.43 | 0.36 | 0.91 | (0.83, 1.01) | 0.0835 |
| rs7383312 | 31576525 | 0.43 | 0.36 | 0.92 | (0.83, 1.02) | 0.0972 |
| rs12216124 | 31578127 | 0.33 | 0.22 | 0.99 | (0.88, 1.12) | 0.9182 |
| rs7382817 | 31578854 | 0.26 | 0.29 | 0.84 | (0.76, 0.94) | 0.0013 |
| rs2855812 | 31580699 | 0.26 | 0.27 | 1.30 | (1.16, 1.45) | 3.42E-06 |
| rs3093953 | 31582667 | 0.33 | 0.21 | 1.00 | (0.89, 1.12) | 0.9549 |
| rs3132468 | 31583465 | 0.35 | 0.25 | 0.91 | (0.81, 1.02) | 0.0917 |
| rs2246618 | 31586965 | 0.31 | 0.35 | 1.25 | (1.13, 1.39) | 1.22E-05 |
| rs2246626 | 31587105 | 0.34 | 0.38 | 0.86 | (0.78, 0.95) | 0.0032 |
| rs6457443 | 31588377 | 0.06 | 0.09 | 1.01 | (0.86, 1.20) | 0.8697 |
| rs2516399 | 31589278 | 0.06 | 0.10 | 0.97 | (0.83, 1.15) | 0.7444 |
| rs2246986 | 31590182 | 0.06 | 0.10 | 0.97 | (0.82, 1.14) | 0.7128 |
| rs2844494 | 31591394 | 0.37 | 0.32 | 0.90 | (0.81, 0.99) | 0.0359 |
| rs4959079 | 31596858 | 0.05 | 0.06 | 0.84 | (0.69, 1.03) | 0.0961 |
| rs3132454 | 31597623 | 0.28 | 0.36 | 0.90 | (0.81, 1.00) | 0.0432 |
| rs3093993 | 31598704 | 0.30 | 0.25 | 0.93 | (0.83, 1.04) | 0.1946 |
| rs3095226 | 31600004 | 0.29 | 0.25 | 0.90 | (0.80, 1.01) | 0.0711 |
| rs3093986 | 31601400 | 0.25 | 0.19 | 0.98 | (0.87, 1.11) | 0.7603 |
| rs2734574 | 31601867 | 0.42 | 0.39 | 1.15 | (1.05, 1.27) | 0.0045 |
| rs2516483 | 31604455 | 0.10 | 0.17 | 0.97 | (0.85, 1.10) | 0.5963 |
| rs3115537 | 31605814 | 0.25 | 0.19 | 0.96 | (0.85, 1.09) | 0.5653 |
| rs3130056 | 31607333 | 0.25 | 0.19 | 0.97 | (0.86, 1.10) | 0.6219 |
| rs11796 | 31609191 | 0.37 | 0.36 | 1.14 | (1.03, 1.26) | 0.0155 |
| rs2075580 | 31611954 | 0.32 | 0.24 | 0.94 | (0.83, 1.05) | 0.2575 |
| rs3130057 | 31612566 | 0.25 | 0.19 | 0.97 | (0.86, 1.10) | 0.6428 |
| rs2071596 | 31614670 | 0.15 | 0.16 | 0.84 | (0.73, 0.96) | 0.0104 |
| rs2239709 | 31615426 | 0.07 | 0.06 | 0.82 | (0.67, 1.00) | 0.0539 |
| rs3130059 | 31617263 | 0.37 | 0.36 | 1.16 | (1.05, 1.28) | 0.0047 |
| rs2844509 | 31618903 | 0.15 | 0.24 | 0.92 | (0.82, 1.03) | 0.1533 |
| rs2251824 | 31619836 | 0.16 | 0.15 | 0.91 | (0.79, 1.04) | 0.1694 |
| rs2239705 | 31621381 | 0.10 | 0.17 | 0.95 | (0.84, 1.08) | 0.4709 |
| rs2523503 | 31621538 | 0.16 | 0.16 | 0.82 | (0.72, 0.94) | 0.0052 |
| rs2071592 | 31623319 | 0.36 | 0.33 | 1.10 | (0.99, 1.22) | 0.0741 |
| rs2071591 | 31623778 | 0.37 | 0.36 | 1.15 | (1.04, 1.27) | 0.0081 |
| rs6916921 | 31628405 | 0.05 | 0.10 | 0.90 | (0.76, 1.06) | 0.2128 |
| rs2255798 | 31629281 | 0.14 | 0.12 | 0.93 | (0.80, 1.08) | 0.3430 |
| rs6929796 | 31630648 | 0.15 | 0.16 | 0.85 | (0.74, 0.97) | 0.0146 |
| rs2857605 | 31632830 | 0.28 | 0.19 | 0.96 | (0.85, 1.09) | 0.5235 |
| rs2239707 | 31633298 | 0.37 | 0.31 | 0.95 | (0.86, 1.06) | 0.3341 |
| rs2230365 | 31633427 | 0.13 | 0.14 | 0.87 | (0.76, 1.01) | 0.0593 |
| rs4947324 | 31636109 | 0.09 | 0.09 | 0.92 | (0.77, 1.09) | 0.3211 |
| rs2516390 | 31637862 | 0.38 | 0.39 | 0.95 | (0.86, 1.05) | 0.2950 |
| rs928815 | 31639194 | 0.38 | 0.39 | 0.95 | (0.86, 1.05) | 0.3073 |
| rs2857602 | 31641357 | 0.38 | 0.40 | 0.93 | (0.84, 1.02) | 0.1370 |
| rs2844484 | 31644203 | 0.38 | 0.40 | 0.91 | (0.83, 1.01) | 0.0804 |
| rs2009658 | 31646223 | 0.16 | 0.15 | 0.93 | (0.81, 1.07) | 0.2824 |
| rs915654 | 31646476 | 0.32 | 0.32 | 0.85 | (0.77, 0.95) | 0.0025 |
| rs909253 | 31648292 | 0.36 | 0.36 | 1.14 | (1.03, 1.27) | 0.0100 |
| rs2857713 | 31648535 | 0.26 | 0.23 | 0.94 | (0.84, 1.06) | 0.3301 |
| rs1799964 | 31650287 | 0.21 | 0.21 | 0.87 | (0.77, 0.99) | 0.0314 |
| rs1800630 | 31650455 | 0.16 | 0.14 | 0.92 | (0.79, 1.06) | 0.2292 |
| rs3093662 | 31652168 | 0.07 | 0.07 | 0.87 | (0.73, 1.05) | 0.1587 |
| rs3093668 | 31654474 | 0.04 | 0.03 | 0.66 | (0.49, 0.89) | 0.0054 |
| rs2256965 | 31663109 | 0.41 | 0.42 | 0.97 | (0.88, 1.07) | 0.5681 |
| rs2857596 | 31675401 | 0.31 | 0.25 | 1.00 | (0.89, 1.12) | 0.9865 |
| rs2857595 | 31676448 | 0.21 | 0.22 | 1.26 | (1.13, 1.42) | 5.51E-05 |
| rs9348876 | 31683255 | 0.08 | 0.08 | 0.70 | (0.58, 0.84) | 0.0002 |
| rs2844477 | 31686751 | 0.36 | 0.35 | 0.83 | (0.75, 0.92) | 0.0004 |
| rs3132451 | 31690004 | 0.12 | 0.23 | 1.42 | (1.27, 1.59) | 1.09E-09 |
| rs2857597 | 31692979 | 0.31 | 0.25 | 0.99 | (0.89, 1.11) | 0.8654 |
| rs2857697 | 31693198 | 0.37 | 0.38 | 0.81 | (0.73, 0.90) | 3.96E-05 |
| rs2736176 | 31695540 | 0.29 | 0.30 | 0.88 | (0.79, 0.98) | 0.0185 |
| rs3763295 | 31695917 | 0.18 | 0.12 | 0.87 | (0.74, 1.01) | 0.0726 |
| rs2844472 | 31697655 | 0.36 | 0.35 | 0.84 | (0.76, 0.94) | 0.0013 |
| rs2736172 | 31698877 | 0.36 | 0.35 | 0.83 | (0.75, 0.92) | 0.0005 |
| rs2260000 | 31701455 | 0.36 | 0.35 | 0.83 | (0.75, 0.92) | 0.0006 |
| rs2736171 | 31703466 | 0.37 | 0.38 | 0.82 | (0.74, 0.90) | 9.67E-05 |
| rs1046080 | 31703861 | 0.31 | 0.27 | 0.92 | (0.82, 1.03) | 0.1446 |
| rs3132450 | 31704117 | 0.09 | 0.14 | 1.61 | (1.40, 1.84) | 4.22E-12 |
| rs3130626 | 31706468 | 0.11 | 0.23 | 1.41 | (1.26, 1.59) | 7.22E-09 |
| rs2736157 | 31708799 | 0.11 | 0.22 | 1.42 | (1.26, 1.59) | 6.65E-09 |
| rs3115663 | 31709822 | 0.11 | 0.22 | 1.45 | (1.29, 1.62) | 4.33E-10 |
| rs2261033 | 31711570 | 0.48 | 0.44 | 0.83 | (0.75, 0.91) | 0.0002 |
| rs9267522 | 31711749 | 0.11 | 0.21 | 1.41 | (1.25, 1.58) | 1.23E-08 |
| rs2736155 | 31713178 | 0.43 | 0.45 | 0.86 | (0.78, 0.95) | 0.0031 |
| rs2178899 | 31714735 | 0.08 | 0.10 | 0.97 | (0.83, 1.15) | 0.7174 |
| rs755714 | 31717792 | 0.29 | 0.29 | 0.89 | (0.80, 0.99) | 0.0267 |
| rs1077394 | 31718363 | 0.46 | 0.33 | 0.88 | (0.79, 0.98) | 0.0167 |
| rs1077393 | 31718508 | 0.43 | 0.46 | 0.83 | (0.75, 0.92) | 0.0002 |
| rs760293 | 31719756 | 0.27 | 0.17 | 1.05 | (0.92, 1.19) | 0.4920 |
| rs2077102 | 31719819 | 0.18 | 0.14 | 0.77 | (0.66, 0.89) | 0.0003 |
| rs2242656 | 31722081 | 0.28 | 0.21 | 0.96 | (0.85, 1.08) | 0.4816 |
| rs2844463 | 31723146 | 0.06 | 0.12 | 0.96 | (0.82, 1.13) | 0.6409 |
| rs805301 | 31726100 | 0.33 | 0.39 | 1.18 | (1.07, 1.30) | 0.0008 |
| rs3130050 | 31726740 | 0.23 | 0.15 | 1.01 | (0.89, 1.16) | 0.8490 |
| rs3117583 | 31727555 | 0.11 | 0.21 | 1.43 | (1.27, 1.61) | 6.83E-09 |
| rs2242655 | 31735428 | 0.18 | 0.12 | 0.86 | (0.74, 1.01) | 0.0575 |
| rs3130617 | 31735502 | 0.31 | 0.25 | 1.01 | (0.89, 1.14) | 0.9236 |
| rs7029 | 31737932 | 0.24 | 0.25 | 0.90 | (0.80, 1.00) | 0.0535 |
| rs3130618 | 31740113 | 0.11 | 0.21 | 1.43 | (1.27, 1.61) | 2.93E-09 |
| rs2295665 | 31740665 | 0.18 | 0.12 | 0.91 | (0.78, 1.06) | 0.2175 |
| rs4569 | 31745786 | 0.24 | 0.25 | 0.90 | (0.80, 1.00) | 0.0534 |
| rs3115667 | 31751378 | 0.31 | 0.25 | 1.00 | (0.89, 1.11) | 0.9083 |
| rs2280800 | 31754377 | 0.18 | 0.12 | 0.88 | (0.76, 1.03) | 0.1004 |
| rs805290 | 31756382 | 0.24 | 0.25 | 0.89 | (0.80, 1.00) | 0.0501 |
| rs707918 | 31762711 | 0.24 | 0.24 | 0.89 | (0.78, 1.00) | 0.0561 |
| rs707917 | 31765733 | 0.24 | 0.25 | 0.90 | (0.81, 1.01) | 0.0692 |
| rs805281 | 31769468 | 0.24 | 0.25 | 0.91 | (0.81, 1.02) | 0.1076 |
| rs805277 | 31770936 | 0.24 | 0.25 | 0.90 | (0.80, 1.00) | 0.0579 |
| rs805274 | 31773173 | 0.24 | 0.25 | 0.89 | (0.80, 1.00) | 0.0443 |
| rs2295663 | 31777274 | 0.07 | 0.05 | 0.69 | (0.55, 0.87) | 0.0013 |
| rs1266071 | 31777475 | 0.08 | 0.09 | 0.91 | (0.77, 1.09) | 0.3118 |
| rs2242653 | 31783744 | 0.19 | 0.13 | 0.86 | (0.74, 1.00) | 0.0454 |
| rs805287 | 31786709 | 0.23 | 0.26 | 0.91 | (0.81, 1.02) | 0.0938 |
| rs6916278 | 31786753 | 0.05 | 0.05 | 1.08 | (0.88, 1.34) | 0.4585 |
| rs805285 | 31787444 | 0.22 | 0.23 | 0.90 | (0.81, 1.02) | 0.0896 |
| rs15574 | 31794476 | 0.18 | 0.21 | 0.85 | (0.76, 0.96) | 0.0081 |
| rs1065356 | 31794987 | 0.18 | 0.21 | 0.89 | (0.79, 1.00) | 0.0522 |
| rs805292 | 31797988 | 0.18 | 0.20 | 0.88 | (0.78, 1.00) | 0.0440 |
| rs2272592 | 31806331 | 0.29 | 0.18 | 0.95 | (0.83, 1.08) | 0.3960 |
| rs3131383 | 31812273 | 0.10 | 0.14 | 1.63 | (1.41, 1.87) | 7.63E-12 |
| rs2293861 | 31819103 | 0.17 | 0.14 | 0.84 | (0.73, 0.97) | 0.0176 |
| rs3749953 | 31821103 | 0.16 | 0.10 | 0.92 | (0.79, 1.09) | 0.3375 |
| rs3117572 | 31825671 | 0.29 | 0.16 | 0.90 | (0.78, 1.04) | 0.1695 |
| rs2299851 | 31826581 | 0.12 | 0.09 | 0.82 | (0.69, 0.98) | 0.0274 |
| rs3131379 | 31829012 | 0.10 | 0.14 | 1.58 | (1.39, 1.81) | 1.42E-11 |
| rs3117574 | 31833209 | 0.10 | 0.14 | 1.60 | (1.40, 1.82) | 4.83E-12 |
| rs707939 | 31834667 | 0.32 | 0.33 | 0.88 | (0.79, 0.98) | 0.0155 |
| rs707938 | 31837338 | 0.32 | 0.34 | 1.19 | (1.08, 1.32) | 0.0007 |
| rs707937 | 31838993 | 0.20 | 0.19 | 0.89 | (0.79, 1.02) | 0.0853 |
| rs3115671 | 31842324 | 0.10 | 0.14 | 1.63 | (1.42, 1.88) | 7.48E-12 |
| rs707929 | 31850046 | 0.31 | 0.35 | 1.22 | (1.10, 1.35) | 0.0001 |
| rs707926 | 31856799 | 0.17 | 0.15 | 0.90 | (0.79, 1.04) | 0.1536 |
| rs4713488 | 31870290 | 0.04 | 0.02 | 0.70 | (0.48, 1.03) | 0.0654 |
| rs480092 | 31872878 | 0.17 | 0.15 | 0.94 | (0.82, 1.08) | 0.3583 |
| rs2075800 | 31885925 | 0.28 | 0.32 | 0.88 | (0.79, 0.97) | 0.0130 |
| rs2227955 | 31886056 | 0.05 | 0.02 | 0.95 | (0.69, 1.32) | 0.7669 |
| rs1043618 | 31891486 | 0.34 | 0.40 | 1.21 | (1.10, 1.34) | 0.0002 |
| rs2763979 | 31902571 | 0.33 | 0.37 | 1.21 | (1.10, 1.34) | 0.0001 |
| rs2471980 | 31908847 | 0.30 | 0.34 | 1.20 | (1.09, 1.33) | 0.0003 |
| rs9368699 | 31910520 | 0.06 | 0.03 | 0.62 | (0.46, 0.84) | 0.0016 |
| rs733539 | 31915468 | 0.16 | 0.10 | 0.88 | (0.74, 1.04) | 0.1321 |
| rs3115673 | 31918064 | 0.16 | 0.13 | 0.91 | (0.79, 1.05) | 0.2083 |
| rs9267576 | 31920017 | 0.23 | 0.14 | 1.00 | (0.87, 1.14) | 0.9568 |
| rs574914 | 31927355 | 0.16 | 0.14 | 0.92 | (0.81, 1.06) | 0.2624 |
| rs9267649 | 31932807 | 0.28 | 0.18 | 0.91 | (0.80, 1.04) | 0.1585 |
| rs13118 | 31935265 | 0.01 | 0.04 | 0.85 | (0.65, 1.10) | 0.2171 |
| rs4947332 | 31942176 | 0.05 | 0.02 | 0.93 | (0.68, 1.29) | 0.6812 |
| rs660550 | 31945256 | 0.47 | 0.46 | 1.21 | (1.10, 1.33) | 0.0001 |
| rs577272 | 31945942 | 0.47 | 0.46 | 1.23 | (1.12, 1.36) | 3.62E-05 |
| rs644774 | 31946469 | 0.47 | 0.46 | 1.21 | (1.09, 1.34) | 0.0004 |
| rs494620 | 31946692 | 0.38 | 0.41 | 0.82 | (0.74, 0.91) | 0.0001 |
| rs2242665 | 31947288 | 0.47 | 0.46 | 1.21 | (1.10, 1.34) | 0.0002 |
| rs3130481 | 31947735 | 0.42 | 0.48 | 1.21 | (1.09, 1.33) | 0.0002 |
| rs614549 | 31948604 | 0.33 | 0.35 | 0.85 | (0.76, 0.94) | 0.0014 |
| rs605203 | 31954991 | 0.43 | 0.36 | 1.25 | (1.13, 1.38) | 1.59E-05 |
| rs589428 | 31956199 | 0.43 | 0.38 | 1.21 | (1.09, 1.34) | 0.0003 |
| rs652888 | 31959213 | 0.20 | 0.24 | 1.32 | (1.18, 1.48) | 8.66E-07 |
| rs486416 | 31964049 | 0.43 | 0.37 | 1.23 | (1.11, 1.36) | 5.63E-05 |
| rs535586 | 31968316 | 0.43 | 0.36 | 1.24 | (1.12, 1.37) | 2.91E-05 |
| rs659445 | 31972283 | 0.43 | 0.36 | 1.25 | (1.13, 1.38) | 9.51E-06 |
| rs2763982 | 31980530 | 0.29 | 0.33 | 0.83 | (0.75, 0.93) | 0.0006 |
| rs1314000 | 31986412 | 0.09 | 0.14 | 1.62 | (1.41, 1.86) | 5.17E-12 |
| rs644045 | 31991936 | 0.38 | 0.38 | 1.31 | (1.19, 1.45) | 7.70E-08 |
| rs3130683 | 31996346 | 0.22 | 0.15 | 1.03 | (0.90, 1.19) | 0.6372 |
| rs497309 | 32000463 | 0.09 | 0.15 | 1.52 | (1.33, 1.74) | 6.65E-10 |
| rs2734335 | 32001923 | 0.43 | 0.49 | 0.83 | (0.75, 0.92) | 0.0002 |
| rs3020644 | 32002605 | 0.33 | 0.37 | 0.84 | (0.76, 0.93) | 0.0005 |
| rs7746553 | 32003952 | 0.19 | 0.14 | 0.87 | (0.76, 1.00) | 0.0521 |
| rs9332739 | 32011783 | 0.07 | 0.05 | 0.98 | (0.78, 1.22) | 0.8312 |
| rs9332730 | 32019988 | 0.08 | 0.03 | 1.03 | (0.78, 1.37) | 0.8141 |
| rs537160 | 32024379 | 0.38 | 0.37 | 1.32 | (1.19, 1.46) | 3.89E-08 |
| rs1270942 | 32026839 | 0.09 | 0.14 | 1.60 | (1.40, 1.82) | 3.70E-12 |
| rs2072633 | 32027557 | 0.45 | 0.46 | 1.25 | (1.14, 1.38) | 5.37E-06 |
| rs4151664 | 32028852 | 0.06 | 0.05 | 0.82 | (0.66, 1.03) | 0.0813 |
| rs440454 | 32035321 | 0.35 | 0.32 | 1.33 | (1.20, 1.47) | 7.87E-08 |
| rs2280774 | 32036670 | 0.29 | 0.31 | 0.83 | (0.75, 0.92) | 0.0005 |
| rs419788 | 32036778 | 0.35 | 0.33 | 1.31 | (1.18, 1.45) | 2.23E-07 |
| rs592229 | 32038420 | 0.49 | 0.48 | 1.26 | (1.14, 1.40) | 3.14E-06 |
| rs492899 | 32041497 | 0.09 | 0.07 | 0.97 | (0.80, 1.17) | 0.7168 |
| rs389884 | 32048876 | 0.09 | 0.14 | 1.62 | (1.41, 1.85) | 3.83E-12 |
| rs387608 | 32049536 | 0.08 | 0.11 | 0.95 | (0.81, 1.11) | 0.4908 |
| rs6941112 | 32054593 | 0.27 | 0.30 | 0.82 | (0.73, 0.91) | 0.0003 |
| rs389883 | 32055439 | 0.36 | 0.33 | 1.30 | (1.18, 1.45) | 3.13E-07 |
| rs389512 | 32055573 | 0.08 | 0.11 | 0.90 | (0.77, 1.05) | 0.1701 |
| rs1009382 | 32134085 | 0.38 | 0.28 | 1.02 | (0.91, 1.14) | 0.7282 |
| rs3130285 | 32134235 | 0.23 | 0.13 | 1.08 | (0.94, 1.25) | 0.2752 |
| rs1150758 | 32136127 | 0.16 | 0.19 | 1.46 | (1.30, 1.65) | 5.98E-10 |
| rs2071295 | 32146678 | 0.28 | 0.29 | 0.82 | (0.73, 0.91) | 0.0003 |
| rs7774197 | 32154253 | 0.08 | 0.04 | 0.83 | (0.64, 1.08) | 0.1627 |
| rs1150753 | 32167845 | 0.10 | 0.14 | 1.61 | (1.41, 1.85) | 1.97E-12 |
| rs2071293 | 32170665 | 0.28 | 0.29 | 0.84 | (0.75, 0.93) | 0.0012 |
| rs1269854 | 32178816 | 0.03 | 0.04 | 1.02 | (0.80, 1.29) | 0.9072 |
| rs12153855 | 32182782 | 0.11 | 0.10 | 0.82 | (0.70, 0.97) | 0.0170 |
| rs429150 | 32183541 | 0.35 | 0.43 | 0.77 | (0.69, 0.86) | 1.21E-06 |
| rs2269426 | 32184477 | 0.31 | 0.36 | 0.78 | (0.71, 0.87) | 1.85E-06 |
| rs8111 | 32191153 | 0.20 | 0.26 | 0.81 | (0.72, 0.91) | 0.0002 |
| rs8283 | 32191278 | 0.15 | 0.19 | 0.74 | (0.65, 0.84) | 3.82E-06 |
| rs204890 | 32193576 | 0.03 | 0.03 | 0.99 | (0.75, 1.30) | 0.9331 |
| rs3213469 | 32196832 | 0.20 | 0.27 | 0.81 | (0.73, 0.91) | 0.0002 |
| rs1269851 | 32200185 | 0.03 | 0.04 | 1.10 | (0.85, 1.42) | 0.4708 |
| rs204894 | 32201900 | 0.03 | 0.04 | 1.01 | (0.79, 1.30) | 0.9189 |
| rs3830076 | 32204222 | 0.07 | 0.06 | 0.85 | (0.69, 1.04) | 0.1150 |
| rs2555456 | 32210209 | 0.03 | 0.04 | 1.06 | (0.82, 1.36) | 0.6597 |
| rs4713505 | 32212979 | 0.20 | 0.28 | 0.80 | (0.72, 0.89) | 6.56E-05 |
| rs204999 | 32217957 | 0.23 | 0.30 | 1.26 | (1.14, 1.40) | 1.02E-05 |
| rs3134952 | 32221549 | 0.21 | 0.24 | 1.27 | (1.13, 1.42) | 3.13E-05 |
| rs4713506 | 32221958 | 0.19 | 0.25 | 0.82 | (0.73, 0.92) | 0.0004 |
| rs9296009 | 32222493 | 0.29 | 0.19 | 0.94 | (0.83, 1.07) | 0.3628 |
| rs3134608 | 32225949 | 0.22 | 0.24 | 1.32 | (1.18, 1.47) | 1.20E-06 |
| rs1053924 | 32228693 | 0.45 | 0.32 | 0.89 | (0.79, 0.99) | 0.0279 |
| rs3134603 | 32233980 | 0.20 | 0.14 | 1.03 | (0.89, 1.19) | 0.7244 |
| rs3134950 | 32235455 | 0.47 | 0.38 | 0.96 | (0.87, 1.07) | 0.4755 |
| rs2269424 | 32240211 | 0.15 | 0.22 | 0.85 | (0.75, 0.97) | 0.0151 |
| rs1061808 | 32244525 | 0.46 | 0.38 | 0.96 | (0.87, 1.06) | 0.4257 |
| rs3130283 | 32246523 | 0.24 | 0.16 | 0.98 | (0.85, 1.12) | 0.7471 |
| rs3130284 | 32248465 | 0.21 | 0.24 | 1.29 | (1.16, 1.44) | 4.58E-06 |
| rs3131297 | 32248983 | 0.21 | 0.24 | 1.30 | (1.17, 1.46) | 3.09E-06 |
| rs3134946 | 32253971 | 0.21 | 0.24 | 1.31 | (1.17, 1.47) | 2.16E-06 |
| rs3134945 | 32254470 | 0.21 | 0.25 | 1.25 | (1.12, 1.40) | 6.35E-05 |
| rs3132965 | 32254975 | 0.21 | 0.24 | 1.30 | (1.16, 1.45) | 4.65E-06 |
| rs3130349 | 32255674 | 0.17 | 0.21 | 1.31 | (1.17, 1.48) | 5.12E-06 |
| rs1035798 | 32259200 | 0.15 | 0.23 | 0.81 | (0.72, 0.91) | 0.0005 |
| rs3131300 | 32259912 | 0.16 | 0.20 | 1.30 | (1.14, 1.48) | 5.89E-05 |
| rs204994 | 32262976 | 0.18 | 0.24 | 1.29 | (1.16, 1.44) | 6.97E-06 |
| rs204993 | 32263559 | 0.23 | 0.29 | 1.21 | (1.09, 1.34) | 0.0005 |
| rs176095 | 32266297 | 0.18 | 0.25 | 1.27 | (1.13, 1.41) | 2.82E-05 |
| rs204991 | 32269344 | 0.13 | 0.23 | 1.36 | (1.22, 1.53) | 5.98E-08 |
| rs204990 | 32269408 | 0.13 | 0.23 | 1.36 | (1.22, 1.53) | 7.07E-08 |
| rs204989 | 32269830 | 0.13 | 0.22 | 1.38 | (1.23, 1.55) | 2.02E-08 |
| rs2071280 | 32272847 | 0.23 | 0.29 | 0.84 | (0.76, 0.94) | 0.0021 |
| rs2071278 | 32273422 | 0.11 | 0.19 | 1.35 | (1.19, 1.53) | 1.45E-06 |
| rs3134942 | 32276749 | 0.10 | 0.16 | 1.32 | (1.16, 1.52) | 4.83E-05 |
| rs2071287 | 32278411 | 0.39 | 0.49 | 0.96 | (0.87, 1.07) | 0.4798 |
| rs3132935 | 32279053 | 0.17 | 0.22 | 1.26 | (1.11, 1.43) | 0.0002 |
| rs2071277 | 32279661 | 0.39 | 0.48 | 0.97 | (0.88, 1.07) | 0.5075 |
| rs3131296 | 32280971 | 0.10 | 0.17 | 1.45 | (1.28, 1.65) | 3.34E-09 |
| rs2856435 | 32283393 | 0.05 | 0.05 | 0.78 | (0.61, 1.00) | 0.0491 |
| rs2071285 | 32288409 | 0.05 | 0.06 | 0.73 | (0.58, 0.91) | 0.0043 |
| rs206016 | 32289160 | 0.08 | 0.09 | 0.98 | (0.82, 1.16) | 0.8074 |
| rs206015 | 32290737 | 0.08 | 0.09 | 0.95 | (0.80, 1.12) | 0.5255 |
| rs404860 | 32292323 | 0.09 | 0.15 | 0.98 | (0.86, 1.12) | 0.7970 |
| rs384247 | 32292552 | 0.09 | 0.15 | 0.98 | (0.86, 1.13) | 0.8171 |
| rs3134798 | 32292683 | 0.43 | 0.26 | 0.95 | (0.85, 1.07) | 0.4133 |
| rs2854050 | 32293583 | 0.05 | 0.05 | 0.79 | (0.62, 1.00) | 0.0466 |
| rs394657 | 32295001 | 0.38 | 0.46 | 1.06 | (0.95, 1.18) | 0.3134 |
| rs429853 | 32295180 | 0.38 | 0.49 | 1.04 | (0.94, 1.14) | 0.4385 |
| rs431722 | 32295700 | 0.46 | 0.39 | 0.96 | (0.87, 1.06) | 0.4323 |
| rs430916 | 32296077 | 0.22 | 0.25 | 0.88 | (0.78, 0.98) | 0.0184 |
| rs415929 | 32297010 | 0.28 | 0.29 | 0.82 | (0.73, 0.92) | 0.0005 |
| rs45855 | 32297459 | 0.28 | 0.31 | 0.82 | (0.74, 0.92) | 0.0003 |
| rs3132946 | 32298006 | 0.24 | 0.14 | 1.08 | (0.94, 1.24) | 0.2753 |
| rs915895 | 32298195 | 0.24 | 0.36 | 1.15 | (1.03, 1.27) | 0.0087 |
| rs3830041 | 32299317 | 0.07 | 0.07 | 0.91 | (0.74, 1.10) | 0.3160 |
| rs3096702 | 32300309 | 0.47 | 0.36 | 0.94 | (0.85, 1.04) | 0.2241 |
| rs2267644 | 32300538 | 0.05 | 0.06 | 0.80 | (0.64, 0.99) | 0.0424 |
| rs499691 | 32302317 | 0.14 | 0.20 | 0.90 | (0.79, 1.01) | 0.0769 |
| rs1475961 | 32302587 | 0.33 | 0.37 | 0.90 | (0.82, 1.00) | 0.0524 |
| rs3096691 | 32302832 | 0.33 | 0.37 | 0.91 | (0.82, 1.01) | 0.0683 |
| rs532385 | 32303337 | 0.13 | 0.16 | 0.86 | (0.75, 0.99) | 0.0380 |
| rs365053 | 32303966 | 0.18 | 0.23 | 0.85 | (0.76, 0.96) | 0.0070 |
| rs495089 | 32305441 | 0.13 | 0.19 | 0.93 | (0.82, 1.05) | 0.2370 |
| rs440261 | 32306034 | 0.19 | 0.24 | 0.86 | (0.77, 0.97) | 0.0111 |
| rs404890 | 32306845 | 0.30 | 0.42 | 1.13 | (1.02, 1.24) | 0.0193 |
| rs2849015 | 32306914 | 0.30 | 0.42 | 1.11 | (1.00, 1.22) | 0.0409 |
| rs3134926 | 32308125 | 0.20 | 0.29 | 0.91 | (0.81, 1.01) | 0.0713 |
| rs12182351 | 32309685 | 0.19 | 0.24 | 0.86 | (0.76, 0.96) | 0.0081 |
| rs3130299 | 32311515 | 0.19 | 0.23 | 0.86 | (0.77, 0.97) | 0.0096 |
| rs416352 | 32315371 | 0.19 | 0.38 | 1.14 | (1.03, 1.26) | 0.0122 |
| rs382259 | 32317005 | 0.12 | 0.26 | 0.82 | (0.73, 0.92) | 0.0004 |
| rs507778 | 32317839 | 0.20 | 0.39 | 1.11 | (1.01, 1.23) | 0.0330 |
| rs419132 | 32318777 | 0.12 | 0.27 | 0.83 | (0.74, 0.93) | 0.0011 |
| rs563412 | 32323041 | 0.38 | 0.44 | 0.80 | (0.72, 0.88) | 8.52E-06 |
| rs2894240 | 32325824 | 0.38 | 0.43 | 0.81 | (0.74, 0.90) | 3.52E-05 |
| rs3115573 | 32326821 | 0.38 | 0.42 | 0.80 | (0.72, 0.88) | 4.21E-06 |
| rs4959089 | 32327703 | 0.16 | 0.15 | 1.00 | (0.87, 1.14) | 0.9564 |
| rs3115572 | 32328462 | 0.46 | 0.43 | 1.25 | (1.14, 1.38) | 4.99E-06 |
| rs3130315 | 32328663 | 0.38 | 0.42 | 0.80 | (0.73, 0.88) | 8.41E-06 |
| rs3115568 | 32332297 | 0.21 | 0.26 | 1.25 | (1.12, 1.40) | 8.70E-05 |
| rs6908927 | 32332467 | 0.21 | 0.24 | 1.34 | (1.20, 1.50) | 1.26E-07 |
| rs3130332 | 32339525 | 0.21 | 0.24 | 1.30 | (1.16, 1.46) | 8.41E-06 |
| rs3115560 | 32344120 | 0.21 | 0.24 | 1.34 | (1.20, 1.50) | 1.14E-07 |
| rs3096673 | 32345991 | 0.21 | 0.24 | 1.34 | (1.20, 1.50) | 1.34E-07 |
| rs3132945 | 32346658 | 0.21 | 0.25 | 1.36 | (1.21, 1.51) | 5.53E-08 |
| rs3115553 | 32353805 | 0.21 | 0.24 | 1.34 | (1.20, 1.50) | 1.56E-07 |
| rs9268132 | 32362632 | 0.36 | 0.40 | 0.78 | (0.71, 0.87) | 1.41E-06 |
| rs4713518 | 32365315 | 0.36 | 0.40 | 0.78 | (0.70, 0.86) | 7.32E-07 |
| rs926070 | 32365544 | 0.40 | 0.31 | 1.03 | (0.93, 1.15) | 0.5599 |
| rs9268148 | 32367505 | 0.25 | 0.18 | 1.07 | (0.94, 1.22) | 0.2855 |
| rs560505 | 32369749 | 0.35 | 0.39 | 0.79 | (0.71, 0.87) | 1.59E-06 |
| rs518374 | 32373688 | 0.36 | 0.40 | 0.78 | (0.71, 0.86) | 9.62E-07 |
| rs11751697 | 32374403 | 0.05 | 0.05 | 1.03 | (0.81, 1.30) | 0.8147 |
| rs6909427 | 32376679 | 0.21 | 0.25 | 1.34 | (1.20, 1.49) | 2.59E-07 |
| rs477005 | 32378478 | 0.35 | 0.39 | 0.78 | (0.70, 0.86) | 6.01E-07 |
| rs1265777 | 32381136 | 0.36 | 0.39 | 0.79 | (0.71, 0.87) | 1.65E-06 |
| rs7341328 | 32383172 | 0.21 | 0.23 | 1.34 | (1.18, 1.51) | 3.14E-06 |
| rs502626 | 32386244 | 0.36 | 0.40 | 0.78 | (0.71, 0.86) | 9.31E-07 |
| rs6910071 | 32390832 | 0.27 | 0.20 | 0.79 | (0.70, 0.90) | 0.0002 |
| rs547077 | 32397296 | 0.36 | 0.40 | 0.79 | (0.71, 0.87) | 1.48E-06 |
| rs508805 | 32403328 | 0.36 | 0.40 | 0.79 | (0.71, 0.87) | 2.61E-06 |
| rs3129949 | 32406792 | 0.19 | 0.23 | 1.24 | (1.11, 1.39) | 0.0002 |
| rs3132959 | 32406920 | 0.31 | 0.24 | 1.05 | (0.94, 1.18) | 0.3940 |
| rs742582 | 32407693 | 0.36 | 0.40 | 0.79 | (0.71, 0.87) | 1.76E-06 |
| rs1003878 | 32407800 | 0.19 | 0.23 | 1.24 | (1.10, 1.38) | 0.0003 |
| rs9366793 | 32409267 | 0.36 | 0.40 | 0.79 | (0.71, 0.87) | 3.04E-06 |
| rs1474728 | 32410045 | 0.36 | 0.39 | 0.79 | (0.71, 0.87) | 6.90E-06 |
| rs6929776 | 32411489 | 0.36 | 0.40 | 0.79 | (0.72, 0.88) | 4.64E-06 |
| rs1265754 | 32411670 | 0.09 | 0.13 | 1.65 | (1.42, 1.90) | 1.44E-11 |
| rs2273019 | 32414397 | 0.33 | 0.36 | 1.23 | (1.11, 1.36) | 5.06E-05 |
| rs2022534 | 32415115 | 0.36 | 0.39 | 0.79 | (0.72, 0.87) | 3.24E-06 |
| rs2143465 | 32417330 | 0.36 | 0.40 | 0.78 | (0.70, 0.86) | 6.28E-07 |
| rs3129904 | 32418374 | 0.31 | 0.25 | 0.98 | (0.87, 1.11) | 0.7676 |
| rs4576282 | 32420774 | 0.36 | 0.40 | 0.78 | (0.71, 0.87) | 1.18E-06 |
| rs969891 | 32424393 | 0.33 | 0.36 | 1.23 | (1.11, 1.36) | 5.55E-05 |
| rs3117120 | 32424782 | 0.33 | 0.36 | 1.23 | (1.11, 1.36) | 5.29E-05 |
| rs2076538 | 32425449 | 0.36 | 0.40 | 0.79 | (0.71, 0.87) | 3.10E-06 |
| rs2076537 | 32425613 | 0.33 | 0.36 | 1.22 | (1.11, 1.35) | 6.21E-05 |
| rs761188 | 32425951 | 0.33 | 0.36 | 1.22 | (1.11, 1.35) | 6.35E-05 |
| rs2022544 | 32428982 | 0.14 | 0.11 | 0.99 | (0.85, 1.15) | 0.8836 |
| rs1265762 | 32429093 | 0.48 | 0.41 | 1.03 | (0.93, 1.13) | 0.5987 |
| rs2395148 | 32429532 | 0.05 | 0.02 | 1.04 | (0.75, 1.43) | 0.8159 |
| rs1265760 | 32429850 | 0.48 | 0.41 | 1.03 | (0.93, 1.14) | 0.5653 |
| rs3129907 | 32431723 | 0.31 | 0.24 | 1.05 | (0.94, 1.18) | 0.3858 |
| rs2206618 | 32432522 | 0.18 | 0.17 | 0.97 | (0.86, 1.11) | 0.6916 |
| rs6907322 | 32432923 | 0.18 | 0.16 | 0.92 | (0.80, 1.05) | 0.2110 |
| rs2395150 | 32434023 | 0.48 | 0.41 | 1.01 | (0.91, 1.11) | 0.8879 |
| rs6904636 | 32435759 | 0.48 | 0.41 | 1.02 | (0.93, 1.13) | 0.6580 |
| rs7452809 | 32436215 | 0.18 | 0.17 | 0.98 | (0.86, 1.12) | 0.7746 |
| rs7763071 | 32437622 | 0.48 | 0.41 | 1.03 | (0.93, 1.14) | 0.5598 |
| rs6457553 | 32438673 | 0.14 | 0.12 | 1.00 | (0.87, 1.17) | 0.9584 |
| rs7770048 | 32442732 | 0.14 | 0.11 | 0.96 | (0.83, 1.13) | 0.6442 |
| rs2143462 | 32443182 | 0.16 | 0.20 | 1.39 | (1.23, 1.56) | 6.95E-08 |
| rs2073048 | 32443411 | 0.14 | 0.12 | 1.02 | (0.88, 1.18) | 0.7982 |
| rs3129937 | 32444342 | 0.16 | 0.19 | 1.36 | (1.20, 1.54) | 1.03E-06 |
| rs3129938 | 32444473 | 0.25 | 0.18 | 1.04 | (0.92, 1.18) | 0.5257 |
| rs3129939 | 32444744 | 0.16 | 0.19 | 1.37 | (1.21, 1.55) | 3.77E-07 |
| rs3129943 | 32446673 | 0.24 | 0.26 | 1.28 | (1.14, 1.43) | 1.64E-05 |
| rs2050191 | 32446879 | 0.31 | 0.24 | 1.05 | (0.94, 1.18) | 0.3885 |
| rs2050190 | 32447054 | 0.26 | 0.34 | 1.18 | (1.07, 1.31) | 0.0013 |
| rs2050189 | 32447625 | 0.18 | 0.24 | 1.23 | (1.10, 1.37) | 0.0004 |
| rs6913309 | 32447818 | 0.24 | 0.25 | 0.92 | (0.82, 1.03) | 0.1314 |
| rs2050188 | 32447875 | 0.37 | 0.36 | 0.89 | (0.80, 0.98) | 0.0197 |
| rs3129944 | 32448850 | 0.14 | 0.25 | 1.40 | (1.26, 1.57) | 1.24E-09 |
| rs7746019 | 32450515 | 0.14 | 0.25 | 1.44 | (1.29, 1.61) | 8.80E-11 |
| rs2395153 | 32453573 | 0.43 | 0.36 | 0.87 | (0.79, 0.96) | 0.0063 |
| rs8180664 | 32455468 | 0.32 | 0.26 | 0.89 | (0.79, 0.99) | 0.0394 |
| rs3117103 | 32457535 | 0.09 | 0.15 | 1.63 | (1.43, 1.85) | 2.80E-13 |
| rs6930777 | 32459544 | 0.12 | 0.09 | 0.87 | (0.73, 1.03) | 0.1019 |
| rs1555115 | 32462498 | 0.13 | 0.11 | 0.85 | (0.73, 1.00) | 0.0470 |
| rs3129948 | 32462622 | 0.36 | 0.33 | 0.86 | (0.77, 0.96) | 0.0092 |
| rs9268473 | 32463661 | 0.45 | 0.39 | 0.85 | (0.76, 0.94) | 0.0012 |
| rs3117098 | 32466491 | 0.36 | 0.32 | 0.89 | (0.80, 0.98) | 0.0208 |
| rs3817969 | 32469366 | 0.13 | 0.14 | 0.89 | (0.77, 1.04) | 0.1323 |
| rs9268480 | 32471822 | 0.32 | 0.26 | 0.92 | (0.82, 1.03) | 0.1601 |
| rs3129954 | 32473558 | 0.36 | 0.32 | 0.87 | (0.78, 0.97) | 0.0095 |
| rs4248166 | 32474399 | 0.16 | 0.15 | 0.90 | (0.79, 1.04) | 0.1475 |
| rs2294883 | 32475429 | 0.16 | 0.15 | 0.90 | (0.78, 1.03) | 0.1166 |
| rs2294881 | 32475582 | 0.16 | 0.15 | 0.92 | (0.81, 1.06) | 0.2377 |
| rs3817964 | 32475975 | 0.09 | 0.04 | 0.82 | (0.65, 1.05) | 0.1109 |
| rs2076524 | 32478662 | 0.32 | 0.27 | 0.91 | (0.81, 1.02) | 0.0881 |
| rs2076522 | 32479157 | 0.32 | 0.26 | 0.90 | (0.81, 1.01) | 0.0728 |
| rs3806156 | 32481676 | 0.37 | 0.33 | 0.90 | (0.81, 1.00) | 0.0511 |
| rs3806157 | 32481779 | 0.37 | 0.33 | 0.93 | (0.83, 1.03) | 0.1417 |
| rs3763313 | 32484449 | 0.19 | 0.17 | 0.90 | (0.79, 1.02) | 0.1072 |
| rs3763317 | 32484766 | 0.47 | 0.41 | 0.85 | (0.77, 0.94) | 0.0018 |
| rs5007259 | 32487079 | 0.48 | 0.48 | 0.84 | (0.76, 0.93) | 0.0005 |
| rs9268516 | 32487467 | 0.32 | 0.26 | 0.91 | (0.82, 1.02) | 0.1176 |
| rs4502931 | 32488760 | 0.48 | 0.48 | 0.83 | (0.75, 0.93) | 0.0008 |
| rs7759742 | 32489714 | 0.48 | 0.48 | 0.84 | (0.75, 0.93) | 0.0006 |
| rs743862 | 32489917 | 0.29 | 0.34 | 1.20 | (1.08, 1.32) | 0.0005 |
| rs3135382 | 32491419 | 0.13 | 0.20 | 1.39 | (1.24, 1.57) | 4.24E-08 |
| rs3135380 | 32492655 | 0.13 | 0.19 | 1.42 | (1.26, 1.60) | 8.81E-09 |
| rs2395161 | 32495730 | 0.13 | 0.20 | 1.45 | (1.29, 1.64) | 1.19E-09 |
| rs3135363 | 32497626 | 0.18 | 0.31 | 1.21 | (1.09, 1.35) | 0.0004 |
| rs3135353 | 32500855 | 0.11 | 0.18 | 1.50 | (1.33, 1.70) | 1.22E-10 |
| rs2187818 | 32503546 | 0.38 | 0.36 | 0.81 | (0.73, 0.90) | 4.94E-05 |
| rs3135340 | 32506850 | 0.23 | 0.29 | 1.34 | (1.20, 1.50) | 1.92E-07 |
| rs3135339 | 32507239 | 0.23 | 0.30 | 1.25 | (1.13, 1.39) | 2.65E-05 |
| rs2395172 | 32507820 | 0.23 | 0.30 | 1.26 | (1.14, 1.40) | 1.25E-05 |
| rs9268606 | 32508048 | 0.38 | 0.36 | 0.83 | (0.75, 0.92) | 0.0005 |
| rs984778 | 32508066 | 0.39 | 0.33 | 1.00 | (0.90, 1.11) | 0.9444 |
| rs9501626 | 32508322 | 0.08 | 0.12 | 0.91 | (0.78, 1.07) | 0.2475 |
| rs3129858 | 32508498 | 0.23 | 0.30 | 1.25 | (1.12, 1.39) | 3.17E-05 |
| rs3129859 | 32508917 | 0.25 | 0.33 | 1.24 | (1.12, 1.37) | 3.29E-05 |
| rs3135338 | 32509195 | 0.39 | 0.33 | 1.01 | (0.91, 1.13) | 0.7907 |
| rs3135335 | 32509823 | 0.39 | 0.31 | 1.00 | (0.89, 1.12) | 0.9404 |
| rs983561 | 32511633 | 0.23 | 0.30 | 1.25 | (1.13, 1.39) | 2.44E-05 |
| rs3129867 | 32512198 | 0.38 | 0.31 | 1.04 | (0.94, 1.16) | 0.4558 |
| rs2395175 | 32513004 | 0.23 | 0.15 | 0.82 | (0.71, 0.94) | 0.0045 |
| rs2395177 | 32513054 | 0.23 | 0.29 | 1.28 | (1.15, 1.43) | 7.60E-06 |
| rs3135395 | 32513170 | 0.39 | 0.33 | 1.01 | (0.91, 1.11) | 0.9265 |
| rs2395178 | 32513340 | 0.39 | 0.32 | 0.99 | (0.89, 1.11) | 0.9191 |
| rs3129871 | 32514320 | 0.41 | 0.35 | 1.01 | (0.91, 1.11) | 0.9303 |
| rs3129872 | 32515131 | 0.23 | 0.30 | 1.25 | (1.13, 1.39) | 1.79E-05 |
| rs2395181 | 32515382 | 0.23 | 0.30 | 1.25 | (1.13, 1.39) | 2.90E-05 |
| rs14004 | 32515687 | 0.38 | 0.37 | 0.80 | (0.73, 0.89) | 1.75E-05 |
| rs3129878 | 32516713 | 0.23 | 0.32 | 1.21 | (1.10, 1.34) | 0.0002 |
| rs3129881 | 32517462 | 0.23 | 0.30 | 1.26 | (1.13, 1.40) | 1.53E-05 |
| rs3129882 | 32517508 | 0.45 | 0.43 | 0.89 | (0.80, 0.98) | 0.0149 |
| rs3129883 | 32518115 | 0.34 | 0.26 | 0.97 | (0.87, 1.09) | 0.6473 |
| rs3135391 | 32518965 | 0.25 | 0.16 | 1.11 | (0.97, 1.27) | 0.1284 |
| rs8084 | 32519013 | 0.47 | 0.46 | 1.29 | (1.17, 1.42) | 3.57E-07 |
| rs2239806 | 32519285 | 0.13 | 0.20 | 1.46 | (1.30, 1.65) | 3.33E-10 |
| rs2239805 | 32519354 | 0.13 | 0.21 | 1.40 | (1.25, 1.58) | 2.03E-08 |
| rs2239804 | 32519501 | 0.43 | 0.43 | 0.83 | (0.75, 0.91) | 0.0002 |
| rs7192 | 32519624 | 0.42 | 0.44 | 1.32 | (1.20, 1.46) | 1.04E-08 |
| rs3129888 | 32519704 | 0.29 | 0.21 | 1.04 | (0.93, 1.18) | 0.4809 |
| rs7194 | 32520458 | 0.42 | 0.44 | 1.32 | (1.20, 1.45) | 1.87E-08 |
| rs3135388 | 32521029 | 0.25 | 0.16 | 1.09 | (0.96, 1.25) | 0.1990 |
| rs2395182 | 32521295 | 0.29 | 0.23 | 1.03 | (0.92, 1.16) | 0.5738 |
| rs2227139 | 32521437 | 0.42 | 0.44 | 1.30 | (1.18, 1.43) | 1.38E-07 |
| rs3129889 | 32521523 | 0.25 | 0.15 | 1.11 | (0.97, 1.26) | 0.1427 |
| rs3763326 | 32521535 | 0.02 | 0.02 | 0.84 | (0.59, 1.20) | 0.3335 |
| rs3763327 | 32521808 | 0.41 | 0.43 | 1.31 | (1.18, 1.44) | 9.46E-08 |
| rs3129890 | 32522251 | 0.16 | 0.28 | 1.31 | (1.18, 1.46) | 3.57E-07 |
| rs7452076 | 32523058 | 0.13 | 0.23 | 1.39 | (1.24, 1.55) | 6.58E-09 |
| rs9296032 | 32526688 | 0.38 | 0.38 | 1.35 | (1.22, 1.49) | 2.99E-09 |
| rs10947279 | 32529776 | 0.04 | 0.07 | 0.90 | (0.74, 1.09) | 0.2662 |
| rs6903608 | 32536263 | 0.33 | 0.32 | 0.92 | (0.83, 1.02) | 0.0983 |
| rs7766843 | 32538707 | 0.16 | 0.28 | 1.27 | (1.14, 1.41) | 9.56E-06 |
| rs2395185 | 32541145 | 0.39 | 0.29 | 0.92 | (0.82, 1.02) | 0.1193 |
| rs9269043 | 32546576 | 0.16 | 0.28 | 1.30 | (1.17, 1.45) | 1.46E-06 |
| rs6901541 | 32550239 | 0.14 | 0.22 | 1.37 | (1.21, 1.55) | 7.11E-07 |
| rs5020946 | 32558033 | 0.49 | 0.39 | 0.89 | (0.81, 0.99) | 0.0269 |
| rs12191360 | 32559339 | 0.33 | 0.33 | 0.90 | (0.81, 1.00) | 0.0449 |
| rs6916742 | 32561169 | 0.36 | 0.45 | 0.94 | (0.85, 1.04) | 0.2351 |
| rs2894266 | 32572876 | 0.12 | 0.17 | 1.43 | (1.25, 1.62) | 4.52E-08 |
| rs2516049 | 32678378 | 0.40 | 0.28 | 0.87 | (0.77, 0.98) | 0.0190 |
| rs660895 | 32685358 | 0.28 | 0.18 | 0.99 | (0.87, 1.12) | 0.8780 |
| rs7451962 | 32690413 | 0.28 | 0.43 | 1.04 | (0.94, 1.15) | 0.4802 |
| rs3129763 | 32698903 | 0.12 | 0.27 | 1.18 | (1.06, 1.32) | 0.0026 |
| rs9271775 | 32702306 | 0.28 | 0.19 | 1.10 | (0.97, 1.25) | 0.1547 |
| rs9272346 | 32712350 | 0.45 | 0.39 | 0.98 | (0.89, 1.09) | 0.7455 |
| rs2187668 | 32713862 | 0.09 | 0.16 | 1.52 | (1.34, 1.73) | 1.29E-10 |
| rs9272535 | 32714734 | 0.17 | 0.31 | 1.17 | (1.05, 1.30) | 0.0053 |
| rs9272723 | 32717405 | 0.44 | 0.37 | 0.90 | (0.82, 1.00) | 0.0536 |
| rs7744001 | 32734064 | 0.23 | 0.31 | 0.82 | (0.74, 0.91) | 0.0002 |
| rs6906021 | 32734289 | 0.33 | 0.50 | 1.08 | (0.98, 1.19) | 0.1176 |
| rs3891175 | 32742445 | 0.17 | 0.24 | 1.40 | (1.25, 1.57) | 6.37E-09 |
| rs4988889 | 32742929 | 0.17 | 0.25 | 1.38 | (1.23, 1.55) | 1.91E-08 |
| rs3134975 | 32760559 | 0.31 | 0.25 | 1.05 | (0.94, 1.18) | 0.3985 |
| rs4947342 | 32761048 | 0.28 | 0.23 | 1.03 | (0.91, 1.16) | 0.6401 |
| rs2856683 | 32763196 | 0.28 | 0.23 | 1.02 | (0.90, 1.14) | 0.8054 |
| rs7775228 | 32766057 | 0.10 | 0.13 | 1.01 | (0.87, 1.17) | 0.8784 |
| rs5002702 | 32767136 | 0.50 | 0.47 | 0.76 | (0.69, 0.84) | 3.55E-08 |
| rs3129719 | 32769757 | 0.31 | 0.25 | 1.05 | (0.94, 1.18) | 0.3778 |
| rs2856726 | 32774699 | 0.27 | 0.33 | 0.77 | (0.70, 0.86) | 1.06E-06 |
| rs2856718 | 32778233 | 0.27 | 0.33 | 0.75 | (0.67, 0.83) | 6.80E-08 |
| rs17499655 | 32780113 | 0.13 | 0.13 | 0.92 | (0.79, 1.06) | 0.2321 |
| rs9275555 | 32785066 | 0.28 | 0.21 | 0.92 | (0.82, 1.04) | 0.1977 |
| rs7764856 | 32788618 | 0.39 | 0.30 | 0.89 | (0.80, 0.99) | 0.0344 |
| rs2858331 | 32789255 | 0.29 | 0.37 | 0.79 | (0.71, 0.87) | 2.98E-06 |
| rs3916766 | 32789623 | 0.18 | 0.27 | 0.79 | (0.70, 0.88) | 2.42E-05 |
| rs3892710 | 32790840 | 0.15 | 0.16 | 1.01 | (0.89, 1.15) | 0.8446 |
| rs3916765 | 32793528 | 0.16 | 0.11 | 1.01 | (0.87, 1.17) | 0.9175 |
| rs3104401 | 32795336 | 0.12 | 0.12 | 0.86 | (0.74, 1.00) | 0.0436 |
| rs763026 | 32799723 | 0.17 | 0.23 | 1.32 | (1.18, 1.48) | 1.82E-06 |
| rs5029394 | 32803060 | 0.36 | 0.25 | 1.14 | (1.02, 1.27) | 0.0255 |
| rs2859113 | 32805763 | 0.48 | 0.46 | 1.30 | (1.17, 1.44) | 6.14E-07 |
| rs2859091 | 32808777 | 0.36 | 0.38 | 0.78 | (0.71, 0.87) | 1.62E-06 |
| rs6913505 | 32810359 | 0.17 | 0.23 | 1.31 | (1.16, 1.47) | 6.51E-06 |
| rs7744593 | 32812304 | 0.35 | 0.25 | 1.13 | (1.01, 1.27) | 0.0288 |
| rs7773068 | 32814212 | 0.35 | 0.24 | 1.10 | (0.98, 1.24) | 0.0976 |
| rs7773694 | 32814312 | 0.35 | 0.25 | 1.14 | (1.02, 1.27) | 0.0248 |
| rs7773955 | 32814697 | 0.36 | 0.39 | 0.76 | (0.69, 0.84) | 1.38E-07 |
| rs5018343 | 32818097 | 0.36 | 0.27 | 1.12 | (1.00, 1.24) | 0.0502 |
| rs9276431 | 32820225 | 0.48 | 0.47 | 1.36 | (1.23, 1.50) | 5.91E-10 |
| rs2239800 | 32821245 | 0.08 | 0.09 | 0.83 | (0.69, 0.99) | 0.0332 |
| rs2213573 | 32824000 | 0.35 | 0.25 | 1.11 | (0.99, 1.25) | 0.0675 |
| rs11758312 | 32824350 | 0.41 | 0.47 | 0.75 | (0.68, 0.82) | 3.46E-09 |
| rs4370403 | 32825493 | 0.08 | 0.06 | 1.12 | (0.92, 1.37) | 0.2526 |
| rs2213571 | 32827915 | 0.48 | 0.47 | 1.33 | (1.21, 1.47) | 3.94E-09 |
| rs2213569 | 32829552 | 0.48 | 0.47 | 1.34 | (1.22, 1.47) | 1.58E-09 |
| rs6918223 | 32830510 | 0.41 | 0.47 | 0.72 | (0.65, 0.79) | 1.99E-11 |
| rs7770024 | 32831319 | 0.41 | 0.47 | 0.71 | (0.65, 0.79) | 7.36E-12 |
| rs7769979 | 32831550 | 0.41 | 0.48 | 0.72 | (0.65, 0.79) | 1.35E-11 |
| rs1023449 | 32835883 | 0.48 | 0.47 | 1.34 | (1.21, 1.47) | 2.51E-09 |
| rs2071550 | 32838918 | 0.28 | 0.30 | 0.79 | (0.71, 0.88) | 1.77E-05 |
| rs1978029 | 32839688 | 0.43 | 0.47 | 0.80 | (0.73, 0.88) | 6.65E-06 |
| rs6902723 | 32839938 | 0.43 | 0.48 | 0.79 | (0.71, 0.87) | 1.53E-06 |
| rs6903130 | 32840188 | 0.43 | 0.47 | 0.79 | (0.72, 0.87) | 2.14E-06 |
| rs9276586 | 32840915 | 0.43 | 0.47 | 0.80 | (0.72, 0.88) | 4.67E-06 |
| rs7382794 | 32842008 | 0.43 | 0.46 | 0.81 | (0.73, 0.89) | 9.87E-06 |
| rs1894412 | 32842807 | 0.43 | 0.47 | 0.81 | (0.73, 0.89) | 2.85E-05 |
| rs9296044 | 32844122 | 0.37 | 0.41 | 0.77 | (0.70, 0.85) | 2.51E-07 |
| rs1383265 | 32847866 | 0.18 | 0.18 | 0.87 | (0.76, 0.99) | 0.0319 |
| rs2857197 | 32854962 | 0.39 | 0.31 | 1.17 | (1.05, 1.30) | 0.0042 |
| rs10947345 | 32857773 | 0.45 | 0.37 | 1.17 | (1.05, 1.29) | 0.0033 |
| rs719654 | 32860117 | 0.21 | 0.20 | 0.80 | (0.71, 0.91) | 0.0004 |
| rs2621393 | 32863194 | 0.39 | 0.31 | 1.19 | (1.07, 1.32) | 0.0011 |
| rs7758736 | 32866372 | 0.15 | 0.19 | 1.37 | (1.21, 1.54) | 2.49E-07 |
| rs3948793 | 32867426 | 0.28 | 0.37 | 0.98 | (0.88, 1.08) | 0.6470 |
| rs2621377 | 32871088 | 0.50 | 0.41 | 1.13 | (1.02, 1.25) | 0.0162 |
| rs7381376 | 32875651 | 0.27 | 0.36 | 1.03 | (0.93, 1.14) | 0.6202 |
| rs6899857 | 32878460 | 0.26 | 0.36 | 1.03 | (0.93, 1.14) | 0.5205 |
| rs7748681 | 32881836 | 0.27 | 0.36 | 1.03 | (0.93, 1.14) | 0.5973 |
| rs6912414 | 32882443 | 0.27 | 0.36 | 0.98 | (0.89, 1.09) | 0.7427 |
| rs2621342 | 32882982 | 0.29 | 0.30 | 0.80 | (0.71, 0.89) | 7.17E-05 |
| rs7382649 | 32884065 | 0.27 | 0.36 | 1.03 | (0.93, 1.14) | 0.5794 |
| rs2857130 | 32884392 | 0.44 | 0.35 | 1.14 | (1.03, 1.26) | 0.0140 |
| rs2621338 | 32884561 | 0.29 | 0.29 | 0.84 | (0.75, 0.94) | 0.0016 |
| rs2857128 | 32884810 | 0.44 | 0.35 | 1.14 | (1.03, 1.26) | 0.0125 |
| rs2857127 | 32884832 | 0.44 | 0.35 | 1.13 | (1.02, 1.25) | 0.0217 |
| rs6929716 | 32885118 | 0.44 | 0.35 | 1.14 | (1.03, 1.26) | 0.0134 |
| rs7454158 | 32885878 | 0.29 | 0.29 | 0.84 | (0.76, 0.94) | 0.0018 |
| rs7383433 | 32886509 | 0.44 | 0.34 | 1.15 | (1.04, 1.28) | 0.0078 |
| rs5009557 | 32887974 | 0.44 | 0.36 | 1.15 | (1.04, 1.27) | 0.0061 |
| rs11244 | 32888702 | 0.21 | 0.29 | 1.10 | (0.99, 1.23) | 0.0772 |
| rs2071479 | 32889090 | 0.04 | 0.03 | 1.01 | (0.75, 1.35) | 0.9693 |
| rs2070121 | 32889532 | 0.08 | 0.07 | 0.83 | (0.68, 1.00) | 0.0535 |
| rs2856997 | 32889754 | 0.40 | 0.38 | 0.81 | (0.73, 0.90) | 5.07E-05 |
| rs2071475 | 32890365 | 0.21 | 0.21 | 0.80 | (0.71, 0.90) | 0.0003 |
| rs2071474 | 32890560 | 0.27 | 0.28 | 0.83 | (0.74, 0.93) | 0.0009 |
| rs7383287 | 32891064 | 0.15 | 0.24 | 1.22 | (1.09, 1.36) | 0.0007 |
| rs2856995 | 32891315 | 0.40 | 0.38 | 0.81 | (0.73, 0.90) | 6.76E-05 |
| rs2621326 | 32891874 | 0.40 | 0.38 | 0.81 | (0.73, 0.89) | 2.34E-05 |
| rs2071554 | 32892654 | 0.05 | 0.04 | 0.84 | (0.66, 1.08) | 0.1670 |
| rs2071469 | 32892761 | 0.35 | 0.34 | 0.80 | (0.72, 0.89) | 2.85E-05 |
| rs2071468 | 32893013 | 0.08 | 0.07 | 0.84 | (0.69, 1.02) | 0.0753 |
| rs2857107 | 32893493 | 0.08 | 0.10 | 0.96 | (0.82, 1.12) | 0.5782 |
| rs2061952 | 32893692 | 0.21 | 0.20 | 0.81 | (0.72, 0.92) | 0.0007 |
| rs1894408 | 32894811 | 0.36 | 0.35 | 0.82 | (0.74, 0.91) | 0.0002 |
| rs3763355 | 32894860 | 0.12 | 0.07 | 1.06 | (0.87, 1.30) | 0.5542 |
| rs3763350 | 32895097 | 0.03 | 0.02 | 1.34 | (0.95, 1.90) | 0.1000 |
| rs2857106 | 32895548 | 0.17 | 0.17 | 0.78 | (0.68, 0.89) | 0.0002 |
| rs2621322 | 32896690 | 0.17 | 0.17 | 0.78 | (0.68, 0.90) | 0.0005 |
| rs2621321 | 32897458 | 0.23 | 0.25 | 0.79 | (0.70, 0.89) | 5.11E-05 |
| rs2857105 | 32898037 | 0.05 | 0.04 | 1.17 | (0.91, 1.51) | 0.2247 |
| rs2857103 | 32899277 | 0.30 | 0.29 | 0.86 | (0.77, 0.96) | 0.0055 |
| rs2856993 | 32899381 | 0.17 | 0.18 | 0.78 | (0.69, 0.89) | 0.0002 |
| rs1894411 | 32900951 | 0.08 | 0.10 | 0.93 | (0.79, 1.09) | 0.3759 |
| rs13501 | 32901501 | 0.30 | 0.30 | 0.84 | (0.76, 0.94) | 0.0018 |
| rs6905503 | 32902097 | 0.03 | 0.02 | 1.04 | (0.73, 1.49) | 0.8130 |
| rs2857101 | 32902654 | 0.23 | 0.25 | 0.81 | (0.72, 0.91) | 0.0003 |
| rs10484565 | 32903010 | 0.10 | 0.09 | 0.68 | (0.57, 0.82) | 3.72E-05 |
| rs241454 | 32904122 | 0.23 | 0.25 | 0.82 | (0.73, 0.91) | 0.0004 |
| rs241439 | 32905515 | 0.34 | 0.40 | 1.02 | (0.93, 1.13) | 0.6473 |
| rs241438 | 32905598 | 0.29 | 0.37 | 1.05 | (0.95, 1.16) | 0.3357 |
| rs241437 | 32905662 | 0.33 | 0.40 | 0.99 | (0.89, 1.09) | 0.8306 |
| rs4576294 | 32906526 | 0.11 | 0.10 | 0.96 | (0.81, 1.13) | 0.5882 |
| rs1015166 | 32906709 | 0.33 | 0.32 | 1.17 | (1.05, 1.31) | 0.0049 |
| rs2228397 | 32908202 | 0.19 | 0.24 | 0.83 | (0.73, 0.94) | 0.0034 |
| rs4148873 | 32908390 | 0.13 | 0.13 | 0.85 | (0.74, 0.98) | 0.0290 |
| rs241432 | 32910181 | 0.44 | 0.38 | 1.02 | (0.92, 1.13) | 0.7167 |
| rs241430 | 32910798 | 0.44 | 0.38 | 1.01 | (0.91, 1.11) | 0.9149 |
| rs4148871 | 32911294 | 0.27 | 0.20 | 1.06 | (0.93, 1.19) | 0.3906 |
| rs241429 | 32911818 | 0.50 | 0.45 | 1.21 | (1.10, 1.33) | 0.0001 |
| rs3819714 | 32912195 | 0.27 | 0.35 | 0.90 | (0.81, 1.00) | 0.0443 |
| rs241426 | 32912531 | 0.39 | 0.40 | 0.94 | (0.84, 1.05) | 0.2708 |
| rs3819721 | 32912776 | 0.29 | 0.23 | 0.91 | (0.81, 1.02) | 0.1082 |
| rs241424 | 32912912 | 0.45 | 0.46 | 1.06 | (0.96, 1.16) | 0.2803 |
| rs2239701 | 32913027 | 0.46 | 0.47 | 0.95 | (0.86, 1.05) | 0.3583 |
| rs2071465 | 32913448 | 0.45 | 0.43 | 0.99 | (0.90, 1.10) | 0.8805 |
| rs2071544 | 32914099 | 0.50 | 0.49 | 1.00 | (0.91, 1.11) | 0.9555 |
| rs3763366 | 32915424 | 0.48 | 0.49 | 1.00 | (0.91, 1.10) | 0.9587 |
| rs9357155 | 32917826 | 0.15 | 0.12 | 0.80 | (0.68, 0.93) | 0.0041 |
| rs2071541 | 32920836 | 0.11 | 0.12 | 0.92 | (0.79, 1.07) | 0.2638 |
| rs2071540 | 32920894 | 0.43 | 0.42 | 1.04 | (0.95, 1.15) | 0.4102 |
| rs1057373 | 32921257 | 0.08 | 0.09 | 0.91 | (0.76, 1.07) | 0.2527 |
| rs4711312 | 32922637 | 0.13 | 0.13 | 0.93 | (0.80, 1.08) | 0.3265 |
| rs1800453 | 32922953 | 0.13 | 0.14 | 0.95 | (0.82, 1.09) | 0.4513 |
| rs735883 | 32924032 | 0.42 | 0.41 | 0.99 | (0.90, 1.09) | 0.8320 |
| rs2071482 | 32924678 | 0.15 | 0.13 | 0.94 | (0.82, 1.09) | 0.4079 |
| rs12529313 | 32925108 | 0.16 | 0.16 | 0.93 | (0.82, 1.07) | 0.3116 |
| rs2395269 | 32925752 | 0.14 | 0.14 | 0.94 | (0.81, 1.08) | 0.3646 |
| rs2071538 | 32926656 | 0.23 | 0.23 | 0.94 | (0.84, 1.06) | 0.3005 |
| rs4148880 | 32926752 | 0.16 | 0.17 | 0.91 | (0.80, 1.04) | 0.1676 |
| rs2284190 | 32927495 | 0.15 | 0.13 | 0.86 | (0.74, 1.00) | 0.0561 |
| rs4713600 | 32930836 | 0.39 | 0.42 | 1.04 | (0.95, 1.15) | 0.3925 |
| rs3763347 | 32930953 | 0.39 | 0.41 | 1.03 | (0.94, 1.14) | 0.5140 |
| rs991760 | 32931545 | 0.06 | 0.06 | 0.84 | (0.68, 1.03) | 0.0988 |
| rs241419 | 32931926 | 0.01 | 0.02 | 0.89 | (0.64, 1.25) | 0.5053 |
| rs2071534 | 32932296 | 0.43 | 0.44 | 1.03 | (0.93, 1.13) | 0.5721 |
| rs9276814 | 32933068 | 0.28 | 0.28 | 1.09 | (0.98, 1.21) | 0.1319 |
| rs2071477 | 32933326 | 0.39 | 0.42 | 1.06 | (0.96, 1.17) | 0.2253 |
| rs6930981 | 32934428 | 0.43 | 0.44 | 1.04 | (0.94, 1.14) | 0.4594 |
| rs9276820 | 32937254 | 0.43 | 0.44 | 1.03 | (0.93, 1.14) | 0.5284 |
| rs7767288 | 32937991 | 0.39 | 0.42 | 1.01 | (0.91, 1.13) | 0.7924 |
| rs3752430 | 32940645 | 0.43 | 0.43 | 1.02 | (0.93, 1.13) | 0.6499 |
| rs1383267 | 32941624 | 0.39 | 0.42 | 1.04 | (0.94, 1.15) | 0.4315 |
| rs1383266 | 32942710 | 0.23 | 0.25 | 0.93 | (0.84, 1.04) | 0.2201 |
| rs7449599 | 32945674 | 0.33 | 0.35 | 1.11 | (1.01, 1.23) | 0.0371 |
| rs6928398 | 32946247 | 0.33 | 0.36 | 1.11 | (1.01, 1.23) | 0.0392 |
| rs7765772 | 32950462 | 0.33 | 0.36 | 1.10 | (0.99, 1.21) | 0.0713 |
| rs6903433 | 32950953 | 0.15 | 0.13 | 0.90 | (0.77, 1.05) | 0.1715 |
| rs7757767 | 32953851 | 0.33 | 0.36 | 1.11 | (1.00, 1.22) | 0.0474 |
| rs4267987 | 32957617 | 0.33 | 0.36 | 1.11 | (1.00, 1.22) | 0.0467 |
| rs7449931 | 32957819 | 0.33 | 0.36 | 1.09 | (0.98, 1.21) | 0.1115 |
| rs10046257 | 32962675 | 0.11 | 0.11 | 0.85 | (0.73, 1.00) | 0.0475 |
| rs2857218 | 32963318 | 0.39 | 0.40 | 1.04 | (0.94, 1.15) | 0.4349 |
| rs2018501 | 32964186 | 0.47 | 0.47 | 0.99 | (0.90, 1.09) | 0.8369 |
| rs1029295 | 32964460 | 0.11 | 0.10 | 0.85 | (0.72, 1.01) | 0.0576 |
| rs241414 | 32965779 | 0.43 | 0.43 | 1.09 | (0.99, 1.20) | 0.0872 |
| rs241407 | 32970718 | 0.11 | 0.09 | 0.93 | (0.78, 1.10) | 0.3800 |
| rs241405 | 32973776 | 0.48 | 0.49 | 0.98 | (0.89, 1.08) | 0.7138 |
| rs241402 | 32977023 | 0.43 | 0.43 | 1.05 | (0.95, 1.16) | 0.3276 |
| rs2187688 | 32979979 | 0.48 | 0.49 | 0.99 | (0.90, 1.09) | 0.8114 |
| rs241398 | 32983428 | 0.43 | 0.42 | 1.02 | (0.92, 1.14) | 0.7028 |
| rs154986 | 32987211 | 0.08 | 0.08 | 0.77 | (0.64, 0.93) | 0.0057 |
| rs154980 | 32990777 | 0.08 | 0.08 | 0.86 | (0.71, 1.04) | 0.1140 |
| rs241458 | 32996544 | 0.48 | 0.49 | 0.99 | (0.90, 1.09) | 0.8152 |
| rs194679 | 32999316 | 0.05 | 0.04 | 0.96 | (0.75, 1.23) | 0.7510 |
| rs9296059 | 33001080 | 0.09 | 0.08 | 0.84 | (0.70, 1.01) | 0.0660 |
| rs6904223 | 33002498 | 0.06 | 0.04 | 0.87 | (0.68, 1.11) | 0.2728 |
| rs11756897 | 33007117 | 0.25 | 0.23 | 0.88 | (0.78, 0.99) | 0.0328 |
| rs154978 | 33007274 | 0.48 | 0.47 | 1.01 | (0.92, 1.11) | 0.8149 |
| rs3132131 | 33007463 | 0.38 | 0.34 | 1.06 | (0.95, 1.17) | 0.2890 |
| rs154977 | 33007996 | 0.33 | 0.34 | 1.28 | (1.15, 1.42) | 2.57E-06 |
| rs154972 | 33008629 | 0.46 | 0.42 | 0.92 | (0.83, 1.02) | 0.0885 |
| rs181997 | 33008696 | 0.40 | 0.35 | 0.94 | (0.85, 1.04) | 0.2418 |
| rs3129299 | 33008765 | 0.22 | 0.16 | 0.95 | (0.83, 1.09) | 0.4868 |
| rs1465651 | 33009329 | 0.20 | 0.14 | 0.90 | (0.78, 1.03) | 0.1295 |
| rs3132132 | 33009912 | 0.20 | 0.15 | 0.84 | (0.73, 0.96) | 0.0118 |
| rs10751 | 33010561 | 0.19 | 0.13 | 0.91 | (0.79, 1.05) | 0.1746 |
| rs23544 | 33011615 | 0.50 | 0.46 | 0.85 | (0.77, 0.94) | 0.0008 |
| rs68600 | 33011702 | 0.50 | 0.47 | 0.86 | (0.78, 0.95) | 0.0018 |
| rs151719 | 33011878 | 0.23 | 0.23 | 1.21 | (1.08, 1.35) | 0.0012 |
| rs1007636 | 33012019 | 0.15 | 0.12 | 0.85 | (0.73, 1.00) | 0.0453 |
| rs2071556 | 33012579 | 0.33 | 0.37 | 1.00 | (0.90, 1.10) | 0.9895 |
| rs194675 | 33013724 | 0.47 | 0.44 | 0.86 | (0.78, 0.95) | 0.0029 |
| rs714289 | 33013789 | 0.09 | 0.06 | 1.21 | (0.99, 1.47) | 0.0612 |
| rs6920787 | 33015480 | 0.04 | 0.03 | 0.85 | (0.64, 1.14) | 0.2821 |
| rs6902982 | 33015859 | 0.04 | 0.03 | 0.83 | (0.63, 1.10) | 0.1914 |
| rs3101944 | 33019105 | 0.08 | 0.09 | 0.92 | (0.77, 1.09) | 0.3329 |
| rs2395296 | 33019792 | 0.25 | 0.31 | 0.99 | (0.88, 1.10) | 0.8320 |
| rs10484567 | 33020530 | 0.02 | 0.01 | 1.59 | (1.10, 2.31) | 0.0139 |
| rs1480380 | 33021224 | 0.08 | 0.09 | 1.37 | (1.16, 1.62) | 0.0002 |
| rs3130595 | 33022275 | 0.08 | 0.08 | 0.97 | (0.81, 1.17) | 0.7717 |
| rs194682 | 33022591 | 0.39 | 0.33 | 0.90 | (0.81, 0.99) | 0.0330 |
| rs6899309 | 33023801 | 0.03 | 0.03 | 1.13 | (0.86, 1.48) | 0.3860 |
| rs1063478 | 33025522 | 0.07 | 0.12 | 0.87 | (0.75, 1.02) | 0.0852 |
| rs1050391 | 33025835 | 0.09 | 0.08 | 1.21 | (1.02, 1.44) | 0.0304 |
| rs3135029 | 33029752 | 0.09 | 0.08 | 1.19 | (1.01, 1.41) | 0.0436 |
| rs150359 | 33030046 | 0.42 | 0.50 | 0.98 | (0.89, 1.08) | 0.6084 |
| rs209473 | 33030886 | 0.38 | 0.45 | 0.96 | (0.87, 1.06) | 0.4557 |
| rs1431393 | 33031966 | 0.08 | 0.09 | 0.85 | (0.71, 1.01) | 0.0681 |
| rs209475 | 33033563 | 0.41 | 0.33 | 0.90 | (0.81, 1.00) | 0.0506 |
| rs580962 | 33033670 | 0.36 | 0.42 | 0.93 | (0.84, 1.02) | 0.1462 |
| rs3129297 | 33035947 | 0.09 | 0.08 | 1.21 | (1.02, 1.43) | 0.0295 |
| rs129653 | 33039514 | 0.41 | 0.35 | 0.90 | (0.81, 1.00) | 0.0446 |
| rs1367727 | 33042387 | 0.08 | 0.08 | 0.89 | (0.75, 1.07) | 0.2054 |
| rs206786 | 33043157 | 0.49 | 0.41 | 0.96 | (0.87, 1.06) | 0.3570 |
| rs683208 | 33045879 | 0.41 | 0.34 | 0.92 | (0.82, 1.03) | 0.1372 |
| rs620202 | 33049888 | 0.30 | 0.28 | 0.92 | (0.83, 1.03) | 0.1373 |
| rs516535 | 33050280 | 0.36 | 0.41 | 0.91 | (0.82, 1.00) | 0.0598 |
| rs485502 | 33051323 | 0.42 | 0.49 | 0.95 | (0.86, 1.04) | 0.2763 |
| rs15912 | 33052072 | 0.08 | 0.08 | 0.92 | (0.78, 1.10) | 0.3772 |
| rs206781 | 33054111 | 0.49 | 0.40 | 0.96 | (0.86, 1.06) | 0.3859 |
| rs2071876 | 33056404 | 0.08 | 0.08 | 0.89 | (0.75, 1.07) | 0.2231 |
| rs184054 | 33057940 | 0.49 | 0.38 | 0.95 | (0.85, 1.06) | 0.3114 |
| rs3135033 | 33058402 | 0.09 | 0.09 | 1.18 | (1.00, 1.40) | 0.0554 |
| rs3135034 | 33059640 | 0.08 | 0.08 | 0.92 | (0.77, 1.10) | 0.3435 |
| rs206777 | 33060524 | 0.36 | 0.41 | 0.92 | (0.83, 1.01) | 0.0820 |
| rs206776 | 33061689 | 0.28 | 0.32 | 0.94 | (0.84, 1.04) | 0.2410 |
| rs188245 | 33063954 | 0.43 | 0.48 | 1.06 | (0.96, 1.17) | 0.2481 |
| rs7382662 | 33067158 | 0.09 | 0.08 | 1.23 | (1.03, 1.47) | 0.0241 |
| rs3128940 | 33071257 | 0.23 | 0.21 | 1.05 | (0.94, 1.19) | 0.3963 |
| rs206765 | 33072674 | 0.48 | 0.39 | 0.95 | (0.86, 1.05) | 0.2604 |
| rs176248 | 33073920 | 0.27 | 0.27 | 0.99 | (0.88, 1.10) | 0.8421 |
| rs12216336 | 33075719 | 0.27 | 0.34 | 1.03 | (0.93, 1.14) | 0.6135 |
| rs2395300 | 33076254 | 0.27 | 0.34 | 1.03 | (0.93, 1.14) | 0.5228 |
| rs2395301 | 33076671 | 0.27 | 0.34 | 1.05 | (0.95, 1.16) | 0.3488 |
| rs172274 | 33077435 | 0.29 | 0.35 | 1.06 | (0.96, 1.18) | 0.2167 |
| rs206762 | 33078428 | 0.50 | 0.43 | 1.03 | (0.94, 1.14) | 0.5574 |
| rs3128931 | 33079686 | 0.21 | 0.24 | 0.85 | (0.75, 0.95) | 0.0050 |
| rs1044429 | 33080620 | 0.11 | 0.14 | 0.94 | (0.81, 1.08) | 0.3467 |
| rs592625 | 33080668 | 0.11 | 0.15 | 0.96 | (0.83, 1.10) | 0.5115 |
| rs3129304 | 33081721 | 0.15 | 0.15 | 1.15 | (1.01, 1.32) | 0.0487 |
| rs2581 | 33082379 | 0.50 | 0.44 | 0.85 | (0.77, 0.94) | 0.0011 |
| rs399604 | 33082992 | 0.48 | 0.43 | 0.84 | (0.76, 0.92) | 0.0004 |
| rs453779 | 33083359 | 0.37 | 0.44 | 1.17 | (1.05, 1.29) | 0.0033 |
| rs2284191 | 33084632 | 0.12 | 0.08 | 1.07 | (0.90, 1.28) | 0.4480 |
| rs403414 | 33085293 | 0.17 | 0.18 | 0.96 | (0.84, 1.09) | 0.5383 |
| rs6911639 | 33086156 | 0.23 | 0.20 | 0.91 | (0.80, 1.03) | 0.1222 |
| rs429916 | 33086565 | 0.05 | 0.08 | 1.18 | (0.99, 1.41) | 0.0531 |
| rs3763342 | 33086975 | 0.12 | 0.14 | 1.06 | (0.92, 1.22) | 0.3950 |
| rs3763341 | 33086998 | 0.12 | 0.12 | 0.99 | (0.85, 1.14) | 0.8750 |
| rs4713603 | 33087587 | 0.46 | 0.45 | 1.00 | (0.91, 1.10) | 0.9801 |
| rs4713604 | 33087748 | 0.34 | 0.36 | 0.97 | (0.87, 1.07) | 0.5624 |
| rs6457699 | 33089625 | 0.46 | 0.45 | 0.99 | (0.90, 1.10) | 0.9172 |
| rs423209 | 33091452 | 0.03 | 0.03 | 1.05 | (0.80, 1.38) | 0.7048 |
| rs6936620 | 33092429 | 0.45 | 0.40 | 0.99 | (0.89, 1.09) | 0.7505 |
| rs2116264 | 33092766 | 0.12 | 0.12 | 0.99 | (0.86, 1.15) | 0.9342 |
| rs3130604 | 33093030 | 0.15 | 0.15 | 1.17 | (1.02, 1.34) | 0.0256 |
| rs2395305 | 33093354 | 0.46 | 0.45 | 1.00 | (0.90, 1.10) | 0.9775 |
| rs4713605 | 33093970 | 0.34 | 0.37 | 0.97 | (0.88, 1.07) | 0.5730 |
| rs3130607 | 33095255 | 0.15 | 0.14 | 1.16 | (1.01, 1.33) | 0.0338 |
| rs9296068 | 33096673 | 0.31 | 0.35 | 1.03 | (0.93, 1.14) | 0.5403 |
| rs3097648 | 33098948 | 0.06 | 0.13 | 0.90 | (0.78, 1.05) | 0.1800 |
| rs12055709 | 33101244 | 0.38 | 0.34 | 0.90 | (0.81, 1.00) | 0.0596 |
| rs443623 | 33104328 | 0.33 | 0.24 | 0.94 | (0.84, 1.05) | 0.2450 |
| rs6920606 | 33105652 | 0.45 | 0.47 | 0.89 | (0.80, 0.98) | 0.0250 |
| rs3130171 | 33106707 | 0.44 | 0.40 | 1.02 | (0.92, 1.12) | 0.7180 |
| rs380468 | 33109466 | 0.14 | 0.28 | 0.99 | (0.89, 1.11) | 0.9421 |
| rs6936967 | 33111043 | 0.14 | 0.28 | 0.99 | (0.89, 1.10) | 0.8501 |
| rs406477 | 33113622 | 0.10 | 0.18 | 1.09 | (0.96, 1.23) | 0.1763 |
| rs663310 | 33114847 | 0.14 | 0.27 | 1.00 | (0.90, 1.12) | 0.9256 |
| rs2116259 | 33116524 | 0.39 | 0.45 | 0.96 | (0.87, 1.06) | 0.4175 |
| rs375912 | 33124706 | 0.36 | 0.31 | 1.01 | (0.91, 1.13) | 0.7778 |
| rs3130176 | 33125435 | 0.50 | 0.42 | 0.97 | (0.87, 1.07) | 0.4768 |
| rs7743563 | 33129170 | 0.20 | 0.20 | 1.13 | (1.00, 1.27) | 0.0579 |
| rs412735 | 33132110 | 0.41 | 0.39 | 0.98 | (0.89, 1.08) | 0.6646 |
| rs435549 | 33132251 | 0.25 | 0.26 | 1.00 | (0.89, 1.11) | 0.9554 |
| rs2116263 | 33133471 | 0.03 | 0.02 | 0.95 | (0.70, 1.30) | 0.7641 |
| rs4604307 | 33133649 | 0.12 | 0.18 | 1.05 | (0.93, 1.20) | 0.4134 |
| rs4551215 | 33133678 | 0.04 | 0.02 | 0.95 | (0.68, 1.32) | 0.7395 |
| rs422544 | 33134088 | 0.23 | 0.27 | 1.03 | (0.92, 1.15) | 0.6213 |
| rs3077 | 33141000 | 0.12 | 0.18 | 1.05 | (0.92, 1.18) | 0.4648 |
| rs2301226 | 33142574 | 0.12 | 0.13 | 1.00 | (0.86, 1.16) | 0.9759 |
| rs1367728 | 33142793 | 0.10 | 0.12 | 1.03 | (0.89, 1.20) | 0.7428 |
| rs2301224 | 33146347 | 0.12 | 0.19 | 1.00 | (0.89, 1.13) | 0.9494 |
| rs2301220 | 33146744 | 0.12 | 0.18 | 1.04 | (0.91, 1.18) | 0.5451 |
| rs6914849 | 33148693 | 0.12 | 0.18 | 1.01 | (0.89, 1.15) | 0.8422 |
| rs1431399 | 33149012 | 0.12 | 0.18 | 1.03 | (0.90, 1.17) | 0.6582 |
| rs9380340 | 33150269 | 0.03 | 0.02 | 1.20 | (0.88, 1.63) | 0.2616 |
| rs987870 | 33150858 | 0.08 | 0.15 | 1.02 | (0.90, 1.17) | 0.7013 |
| rs2071353 | 33152235 | 0.11 | 0.19 | 0.97 | (0.86, 1.10) | 0.7068 |
| rs2071354 | 33152366 | 0.08 | 0.15 | 1.02 | (0.89, 1.16) | 0.8033 |
| rs1431403 | 33155009 | 0.29 | 0.28 | 1.04 | (0.94, 1.16) | 0.4277 |
| rs3097671 | 33155590 | 0.18 | 0.17 | 0.95 | (0.83, 1.09) | 0.4968 |
| rs9378177 | 33157362 | 0.03 | 0.02 | 0.91 | (0.64, 1.28) | 0.5699 |
| rs7772134 | 33157704 | 0.05 | 0.10 | 1.08 | (0.91, 1.28) | 0.3649 |
| rs9277378 | 33158257 | 0.31 | 0.29 | 1.04 | (0.93, 1.16) | 0.4768 |
| rs10947383 | 33160959 | 0.09 | 0.12 | 1.06 | (0.92, 1.23) | 0.4664 |
| rs9277535 | 33162839 | 0.28 | 0.25 | 0.92 | (0.82, 1.03) | 0.1484 |
| rs9277554 | 33163516 | 0.32 | 0.30 | 1.01 | (0.91, 1.13) | 0.8139 |
| rs3117229 | 33164047 | 0.28 | 0.24 | 0.95 | (0.85, 1.07) | 0.3855 |
| rs3128968 | 33164231 | 0.28 | 0.24 | 0.94 | (0.84, 1.06) | 0.3111 |
| rs3117228 | 33164413 | 0.32 | 0.30 | 1.03 | (0.93, 1.14) | 0.5908 |
| rs3130188 | 33165154 | 0.32 | 0.29 | 1.02 | (0.91, 1.14) | 0.7492 |
| rs3117226 | 33165637 | 0.30 | 0.26 | 1.07 | (0.96, 1.19) | 0.2408 |
| rs3097652 | 33165813 | 0.32 | 0.30 | 1.04 | (0.94, 1.16) | 0.4567 |
| rs1367730 | 33166092 | 0.31 | 0.27 | 1.06 | (0.95, 1.18) | 0.3075 |
| rs2068204 | 33166696 | 0.08 | 0.03 | 0.97 | (0.74, 1.27) | 0.8330 |
| rs3128972 | 33166752 | 0.31 | 0.27 | 1.05 | (0.94, 1.17) | 0.4063 |
| rs2179920 | 33166852 | 0.23 | 0.24 | 1.06 | (0.94, 1.19) | 0.3254 |
| rs2281389 | 33167774 | 0.19 | 0.17 | 0.95 | (0.83, 1.08) | 0.4326 |
| rs3117223 | 33168042 | 0.31 | 0.26 | 1.04 | (0.93, 1.17) | 0.4810 |
| rs2295120 | 33168747 | 0.08 | 0.03 | 0.99 | (0.76, 1.29) | 0.9196 |
| rs2295119 | 33168848 | 0.09 | 0.11 | 1.01 | (0.86, 1.18) | 0.9511 |
| rs3130190 | 33169668 | 0.32 | 0.27 | 1.07 | (0.95, 1.19) | 0.2511 |
| rs3130191 | 33169849 | 0.31 | 0.26 | 1.06 | (0.95, 1.19) | 0.2567 |
| rs3117217 | 33171249 | 0.31 | 0.25 | 1.04 | (0.93, 1.17) | 0.4508 |
| rs3117213 | 33172583 | 0.31 | 0.27 | 0.95 | (0.85, 1.07) | 0.3960 |
| rs3128921 | 33178727 | 0.27 | 0.25 | 1.03 | (0.91, 1.15) | 0.6520 |
| rs3128923 | 33179300 | 0.31 | 0.25 | 1.03 | (0.91, 1.16) | 0.6631 |
| rs3117234 | 33181962 | 0.28 | 0.24 | 1.07 | (0.95, 1.20) | 0.2349 |
| rs3130212 | 33182367 | 0.28 | 0.24 | 1.07 | (0.96, 1.21) | 0.2109 |
| rs3130215 | 33182941 | 0.44 | 0.39 | 0.93 | (0.84, 1.03) | 0.1764 |
| rs3117230 | 33183613 | 0.23 | 0.23 | 1.05 | (0.94, 1.18) | 0.3508 |
| rs9380343 | 33187144 | 0.08 | 0.03 | 1.01 | (0.76, 1.36) | 0.9308 |
| rs6937034 | 33187744 | 0.07 | 0.03 | 1.02 | (0.77, 1.35) | 0.9133 |
| rs2016780 | 33190449 | 0.31 | 0.26 | 0.94 | (0.84, 1.06) | 0.3100 |
| rs1810472 | 33191099 | 0.28 | 0.30 | 0.93 | (0.84, 1.04) | 0.2102 |
| rs1883414 | 33194426 | 0.32 | 0.31 | 0.97 | (0.87, 1.08) | 0.6057 |
| rs9277678 | 33195336 | 0.40 | 0.41 | 1.02 | (0.93, 1.13) | 0.6397 |
| rs12210068 | 33197174 | 0.22 | 0.21 | 1.03 | (0.92, 1.16) | 0.5744 |
| rs3117021 | 33200027 | 0.49 | 0.50 | 0.99 | (0.89, 1.09) | 0.7919 |
| rs3129274 | 33202847 | 0.34 | 0.32 | 1.01 | (0.91, 1.12) | 0.7942 |
| rs3117016 | 33203494 | 0.43 | 0.41 | 0.96 | (0.87, 1.06) | 0.4304 |
| rs3117009 | 33204112 | 0.49 | 0.49 | 1.00 | (0.90, 1.10) | 0.9634 |
| rs3117004 | 33204744 | 0.33 | 0.32 | 0.99 | (0.89, 1.10) | 0.8074 |
| rs3129269 | 33205592 | 0.35 | 0.37 | 0.91 | (0.82, 1.00) | 0.0632 |
| rs3116994 | 33206775 | 0.45 | 0.46 | 1.06 | (0.96, 1.17) | 0.2052 |
| rs2294477 | 33206966 | 0.41 | 0.41 | 1.03 | (0.93, 1.14) | 0.5312 |
| rs2294472 | 33207188 | 0.35 | 0.39 | 1.00 | (0.90, 1.10) | 0.9518 |
| rs6919938 | 33209580 | 0.22 | 0.22 | 1.02 | (0.90, 1.14) | 0.7610 |
| rs7750059 | 33211151 | 0.21 | 0.23 | 1.03 | (0.91, 1.15) | 0.6718 |
| rs7750000 | 33211299 | 0.21 | 0.22 | 1.03 | (0.92, 1.16) | 0.6385 |
| rs7383503 | 33215439 | 0.14 | 0.15 | 0.95 | (0.83, 1.09) | 0.4867 |
| rs3129249 | 33217534 | 0.22 | 0.22 | 1.04 | (0.92, 1.16) | 0.5300 |
| rs7750683 | 33218812 | 0.38 | 0.39 | 0.99 | (0.90, 1.10) | 0.8940 |
| rs2395365 | 33224200 | 0.38 | 0.39 | 1.03 | (0.93, 1.13) | 0.6207 |
| rs756441 | 33230149 | 0.38 | 0.39 | 1.03 | (0.93, 1.14) | 0.5994 |
| rs721844 | 33231510 | 0.14 | 0.15 | 0.95 | (0.83, 1.09) | 0.4568 |
| rs3116959 | 33232472 | 0.22 | 0.21 | 1.06 | (0.94, 1.20) | 0.3402 |
| rs3129207 | 33233290 | 0.48 | 0.50 | 1.01 | (0.92, 1.11) | 0.8180 |
| rs3129206 | 33233440 | 0.48 | 0.45 | 1.03 | (0.94, 1.14) | 0.5179 |
| rs3130161 | 33233836 | 0.15 | 0.12 | 1.00 | (0.87, 1.16) | 0.9695 |
| rs3116956 | 33237049 | 0.35 | 0.31 | 1.01 | (0.91, 1.12) | 0.8715 |
| rs9380351 | 33237724 | 0.18 | 0.17 | 0.95 | (0.83, 1.09) | 0.4318 |
| rs2235498 | 33238408 | 0.22 | 0.23 | 0.90 | (0.80, 1.01) | 0.0678 |
| rs986522 | 33243940 | 0.43 | 0.47 | 0.94 | (0.85, 1.04) | 0.2493 |
| rs986521 | 33244123 | 0.30 | 0.24 | 0.92 | (0.82, 1.03) | 0.1433 |
| rs2855448 | 33244553 | 0.38 | 0.34 | 1.02 | (0.92, 1.13) | 0.6758 |
| rs2855437 | 33246933 | 0.33 | 0.30 | 0.91 | (0.81, 1.01) | 0.0767 |
| rs3762013 | 33250517 | 0.48 | 0.44 | 0.93 | (0.85, 1.03) | 0.1849 |
| rs1799908 | 33252221 | 0.38 | 0.42 | 0.99 | (0.89, 1.09) | 0.7549 |
| rs2855425 | 33252351 | 0.32 | 0.28 | 0.93 | (0.83, 1.04) | 0.1778 |
| rs2855459 | 33262634 | 0.16 | 0.14 | 1.00 | (0.87, 1.15) | 0.9846 |
| rs2855429 | 33266167 | 0.32 | 0.26 | 0.92 | (0.82, 1.03) | 0.1577 |
| rs2269346 | 33266876 | 0.05 | 0.07 | 0.70 | (0.57, 0.86) | 0.0006 |
| rs2072915 | 33270060 | 0.30 | 0.31 | 1.10 | (0.99, 1.22) | 0.0722 |
| rs2744537 | 33270193 | 0.32 | 0.27 | 0.91 | (0.82, 1.02) | 0.0950 |
| rs2076310 | 33274012 | 0.23 | 0.25 | 0.90 | (0.80, 1.01) | 0.0762 |
| rs1547387 | 33277873 | 0.13 | 0.10 | 1.09 | (0.93, 1.28) | 0.2694 |
| rs383711 | 33281976 | 0.06 | 0.08 | 0.71 | (0.58, 0.87) | 0.0008 |
| rs421446 | 33282761 | 0.26 | 0.29 | 0.95 | (0.85, 1.06) | 0.3481 |
| rs213208 | 33285988 | 0.23 | 0.24 | 0.90 | (0.80, 1.01) | 0.0775 |
| rs2854028 | 33287667 | 0.23 | 0.24 | 0.96 | (0.85, 1.08) | 0.4774 |
| rs1567464 | 33290848 | 0.13 | 0.12 | 1.04 | (0.90, 1.21) | 0.6240 |
| rs213213 | 33291708 | 0.32 | 0.31 | 0.98 | (0.88, 1.10) | 0.7403 |
| rs1977090 | 33293556 | 0.13 | 0.12 | 1.03 | (0.89, 1.20) | 0.7404 |
| rs213212 | 33293896 | 0.28 | 0.26 | 0.93 | (0.83, 1.03) | 0.1704 |
| rs1606015 | 33300845 | 0.28 | 0.28 | 0.96 | (0.86, 1.07) | 0.4690 |
| rs213194 | 33303582 | 0.27 | 0.26 | 0.91 | (0.81, 1.02) | 0.1183 |
| rs213198 | 33306749 | 0.17 | 0.11 | 1.01 | (0.86, 1.17) | 0.9349 |
| rs213220 | 33310618 | 0.48 | 0.47 | 0.93 | (0.84, 1.03) | 0.1378 |
| rs9277952 | 33312252 | 0.12 | 0.12 | 0.92 | (0.80, 1.07) | 0.3019 |
| rs213226 | 33317288 | 0.48 | 0.48 | 0.96 | (0.87, 1.06) | 0.3569 |
| rs461338 | 33326158 | 0.18 | 0.14 | 0.94 | (0.82, 1.09) | 0.4222 |
| rs462618 | 33330141 | 0.18 | 0.14 | 0.94 | (0.82, 1.08) | 0.3719 |
| rs213202 | 33340033 | 0.33 | 0.36 | 1.09 | (0.99, 1.21) | 0.0779 |
| rs213199 | 33343733 | 0.33 | 0.37 | 1.08 | (0.98, 1.20) | 0.1084 |
| rs213203 | 33346382 | 0.48 | 0.47 | 0.93 | (0.84, 1.03) | 0.1393 |
| rs464921 | 33348484 | 0.18 | 0.14 | 0.96 | (0.83, 1.10) | 0.5632 |
| rs213204 | 33349054 | 0.33 | 0.37 | 1.08 | (0.98, 1.20) | 0.1057 |
| rs213207 | 33349147 | 0.48 | 0.47 | 0.92 | (0.83, 1.01) | 0.0683 |
| rs458679 | 33350470 | 0.18 | 0.16 | 0.89 | (0.78, 1.02) | 0.0925 |
| rs458434 | 33350803 | 0.18 | 0.14 | 0.94 | (0.82, 1.08) | 0.3852 |
| rs463302 | 33352694 | 0.18 | 0.15 | 0.94 | (0.82, 1.08) | 0.4122 |
| rs3445 | 33354496 | 0.18 | 0.15 | 0.94 | (0.82, 1.08) | 0.3855 |
| rs455567 | 33360093 | 0.50 | 0.46 | 1.11 | (1.01, 1.22) | 0.0305 |
| rs466384 | 33362643 | 0.17 | 0.13 | 0.95 | (0.82, 1.10) | 0.4823 |
| rs446735 | 33363081 | 0.50 | 0.46 | 1.12 | (1.01, 1.23) | 0.0266 |
| rs456261 | 33366421 | 0.49 | 0.48 | 1.12 | (1.02, 1.24) | 0.0185 |
| rs1059288 | 33375650 | 0.49 | 0.47 | 1.12 | (1.02, 1.24) | 0.0234 |
| rs2071888 | 33380833 | 0.49 | 0.46 | 1.13 | (1.02, 1.24) | 0.0154 |
| rs2239841 | 33389820 | 0.26 | 0.31 | 0.89 | (0.80, 0.99) | 0.0252 |
| rs3106189 | 33389980 | 0.48 | 0.48 | 1.13 | (1.02, 1.25) | 0.0129 |
| rs2239839 | 33396053 | 0.26 | 0.31 | 0.91 | (0.82, 1.01) | 0.0583 |
| rs1059231 | 33396249 | 0.26 | 0.32 | 0.91 | (0.82, 1.01) | 0.0549 |
| rs3130266 | 33411967 | 0.48 | 0.48 | 1.13 | (1.02, 1.24) | 0.0127 |
| rs3130267 | 33414772 | 0.48 | 0.46 | 1.08 | (0.98, 1.19) | 0.1333 |
| rs3130270 | 33416199 | 0.48 | 0.48 | 1.12 | (1.02, 1.23) | 0.0174 |
| rs3130014 | 33420286 | 0.29 | 0.31 | 1.02 | (0.92, 1.14) | 0.6335 |
| rs3130013 | 33421405 | 0.19 | 0.10 | 1.07 | (0.91, 1.25) | 0.4349 |
| rs211474 | 33428591 | 0.28 | 0.31 | 1.12 | (1.01, 1.25) | 0.0293 |
| rs3117324 | 33432505 | 0.46 | 0.41 | 1.14 | (1.04, 1.26) | 0.0068 |
| rs3106192 | 33434228 | 0.43 | 0.46 | 0.90 | (0.81, 0.99) | 0.0244 |
| rs211453 | 33438109 | 0.35 | 0.36 | 1.15 | (1.04, 1.27) | 0.0055 |
| rs3130275 | 33440115 | 0.33 | 0.37 | 0.91 | (0.82, 1.01) | 0.0544 |
| rs3130276 | 33444908 | 0.33 | 0.38 | 0.88 | (0.79, 0.97) | 0.0130 |
| rs9278027 | 33448488 | 0.12 | 0.13 | 0.92 | (0.80, 1.07) | 0.2829 |
| rs2747479 | 33454164 | 0.35 | 0.36 | 1.12 | (1.02, 1.24) | 0.0194 |
| rs2747476 | 33459229 | 0.19 | 0.10 | 1.07 | (0.91, 1.26) | 0.4120 |
| rs9278036 | 33460338 | 0.12 | 0.13 | 0.94 | (0.81, 1.09) | 0.3822 |
| rs456993 | 33466256 | 0.33 | 0.37 | 0.92 | (0.83, 1.01) | 0.0768 |
| rs465506 | 33466986 | 0.34 | 0.38 | 0.91 | (0.82, 1.01) | 0.0717 |
| rs211457 | 33473618 | 0.16 | 0.10 | 1.04 | (0.89, 1.22) | 0.6001 |
| rs4231 | 33491952 | 0.12 | 0.13 | 0.96 | (0.83, 1.11) | 0.5700 |
| rs211456 | 33497359 | 0.41 | 0.38 | 1.11 | (1.01, 1.23) | 0.0295 |
| rs413722 | 33506598 | 0.12 | 0.07 | 0.94 | (0.77, 1.15) | 0.5563 |
| rs453590 | 33511400 | 0.41 | 0.36 | 1.19 | (1.07, 1.32) | 0.0012 |
| rs10807124 | 33512042 | 0.23 | 0.29 | 0.95 | (0.85, 1.06) | 0.3373 |
| rs2247385 | 33529555 | 0.42 | 0.39 | 1.14 | (1.04, 1.26) | 0.0066 |
| rs2772372 | 33535328 | 0.12 | 0.10 | 0.93 | (0.79, 1.10) | 0.3892 |
| rs2772373 | 33537650 | 0.33 | 0.38 | 0.92 | (0.83, 1.02) | 0.0861 |
| rs7748370 | 33572039 | 0.28 | 0.25 | 1.01 | (0.90, 1.13) | 0.8577 |
| rs769051 | 33581872 | 0.42 | 0.35 | 1.00 | (0.91, 1.11) | 0.9835 |
| rs68191 | 33588716 | 0.10 | 0.09 | 0.93 | (0.79, 1.10) | 0.4198 |
| rs210192 | 33589182 | 0.38 | 0.33 | 0.97 | (0.87, 1.07) | 0.5467 |
| rs210179 | 33593214 | 0.49 | 0.47 | 0.88 | (0.79, 0.97) | 0.0143 |
| rs6929647 | 33596527 | 0.01 | 0.01 | 1.17 | (0.74, 1.84) | 0.5008 |
| rs210188 | 33599265 | 0.39 | 0.32 | 0.99 | (0.88, 1.10) | 0.7853 |
| rs449242 | 33604692 | 0.39 | 0.33 | 0.97 | (0.88, 1.08) | 0.6078 |
| rs9469481 | 33613572 | 0.39 | 0.31 | 0.98 | (0.88, 1.09) | 0.7364 |
| rs396516 | 33614045 | 0.39 | 0.32 | 0.98 | (0.89, 1.09) | 0.7382 |
| rs210196 | 33615701 | 0.38 | 0.30 | 0.99 | (0.89, 1.10) | 0.8216 |
| rs210203 | 33619207 | 0.41 | 0.34 | 0.98 | (0.89, 1.09) | 0.7602 |
| rs210152 | 33623498 | 0.19 | 0.17 | 1.16 | (1.02, 1.31) | 0.0200 |
| rs5000098 | 33627209 | 0.28 | 0.26 | 0.98 | (0.88, 1.10) | 0.7683 |
| rs9366824 | 33629555 | 0.27 | 0.31 | 0.91 | (0.82, 1.01) | 0.0748 |
| rs210170 | 33633658 | 0.33 | 0.36 | 1.06 | (0.96, 1.17) | 0.2442 |
| rs210132 | 33644648 | 0.39 | 0.38 | 0.96 | (0.87, 1.06) | 0.4249 |
| rs210133 | 33644775 | 0.33 | 0.36 | 1.04 | (0.94, 1.15) | 0.3892 |
| rs17627049 | 33645780 | 0.28 | 0.24 | 0.97 | (0.86, 1.09) | 0.6033 |
| rs210134 | 33648187 | 0.32 | 0.29 | 1.10 | (0.99, 1.23) | 0.0622 |
| rs513349 | 33649697 | 0.35 | 0.42 | 0.93 | (0.84, 1.03) | 0.1727 |
| rs210139 | 33651387 | 0.34 | 0.40 | 0.96 | (0.86, 1.06) | 0.3897 |
| rs4559081 | 33663326 | 0.16 | 0.19 | 0.95 | (0.84, 1.08) | 0.4497 |
| rs375555 | 33665719 | 0.20 | 0.20 | 1.01 | (0.89, 1.14) | 0.9307 |
| rs210122 | 33682613 | 0.38 | 0.44 | 1.01 | (0.91, 1.11) | 0.8630 |
| rs499384 | 33690068 | 0.14 | 0.11 | 0.91 | (0.78, 1.07) | 0.2589 |
| rs3748080 | 33695932 | 0.19 | 0.10 | 1.18 | (1.01, 1.38) | 0.0404 |
| rs3748079 | 33696125 | 0.18 | 0.24 | 1.06 | (0.94, 1.19) | 0.3905 |
| rs4711332 | 33703377 | 0.23 | 0.14 | 1.05 | (0.91, 1.20) | 0.4996 |
| rs4713646 | 33714501 | 0.22 | 0.24 | 1.05 | (0.93, 1.17) | 0.4090 |
| rs12205634 | 33719645 | 0.21 | 0.24 | 1.03 | (0.92, 1.15) | 0.6543 |
| rs6457738 | 33725442 | 0.42 | 0.38 | 1.04 | (0.94, 1.15) | 0.4897 |
| rs1570760 | 33730911 | 0.22 | 0.25 | 1.01 | (0.90, 1.13) | 0.9218 |
| rs999943 | 33732711 | 0.23 | 0.32 | 0.99 | (0.89, 1.10) | 0.8486 |
| rs2296343 | 33734695 | 0.33 | 0.28 | 1.03 | (0.92, 1.16) | 0.5905 |
| rs2296340 | 33738450 | 0.18 | 0.10 | 1.13 | (0.96, 1.32) | 0.1516 |
| rs2296339 | 33738602 | 0.42 | 0.46 | 1.03 | (0.94, 1.14) | 0.5121 |
| rs2296338 | 33738794 | 0.48 | 0.42 | 1.00 | (0.91, 1.11) | 0.9896 |
| rs2296337 | 33739674 | 0.32 | 0.28 | 1.01 | (0.90, 1.13) | 0.8875 |
| rs2296336 | 33744638 | 0.37 | 0.28 | 1.04 | (0.93, 1.17) | 0.4592 |
| rs2077163 | 33744885 | 0.48 | 0.47 | 0.94 | (0.85, 1.04) | 0.1826 |
| rs2229634 | 33746158 | 0.37 | 0.29 | 1.04 | (0.94, 1.16) | 0.4235 |
| rs3736893 | 33747738 | 0.34 | 0.33 | 0.96 | (0.86, 1.06) | 0.4245 |
| rs2229638 | 33756206 | 0.17 | 0.09 | 1.13 | (0.95, 1.34) | 0.1592 |
| rs2296330 | 33758599 | 0.20 | 0.25 | 0.92 | (0.82, 1.04) | 0.1682 |
| rs2296329 | 33758721 | 0.19 | 0.20 | 0.99 | (0.87, 1.12) | 0.8555 |
| rs3818521 | 33765224 | 0.38 | 0.46 | 0.96 | (0.87, 1.06) | 0.3938 |
| rs2229642 | 33767450 | 0.43 | 0.48 | 0.98 | (0.88, 1.08) | 0.6394 |
| rs3818527 | 33769013 | 0.40 | 0.47 | 0.95 | (0.86, 1.04) | 0.2497 |
| rs658087 | 33772998 | 0.20 | 0.14 | 0.96 | (0.83, 1.11) | 0.5552 |
| rs4713658 | 33775095 | 0.43 | 0.49 | 0.96 | (0.87, 1.06) | 0.3844 |
| rs626156 | 33778460 | 0.41 | 0.38 | 0.97 | (0.88, 1.07) | 0.5525 |
| rs525623 | 33780581 | 0.15 | 0.13 | 1.17 | (1.02, 1.35) | 0.0249 |
| rs2281829 | 33783620 | 0.39 | 0.47 | 0.96 | (0.87, 1.06) | 0.3676 |
| rs542441 | 33783829 | 0.41 | 0.39 | 0.98 | (0.89, 1.09) | 0.6989 |
| rs3818532 | 33787785 | 0.13 | 0.19 | 0.95 | (0.84, 1.08) | 0.4677 |
| rs9394163 | 33790392 | 0.40 | 0.46 | 0.97 | (0.88, 1.07) | 0.5164 |
| rs549652 | 33794081 | 0.23 | 0.14 | 0.98 | (0.85, 1.13) | 0.7963 |
| rs13214874 | 33794346 | 0.13 | 0.19 | 0.98 | (0.86, 1.11) | 0.6952 |
| rs630792 | 33798288 | 0.13 | 0.08 | 0.90 | (0.75, 1.07) | 0.2217 |
| rs12203688 | 33801602 | 0.13 | 0.18 | 0.95 | (0.84, 1.09) | 0.4714 |
| rs877187 | 33814189 | 0.10 | 0.16 | 1.04 | (0.91, 1.18) | 0.6002 |
| rs6942022 | 33818262 | 0.12 | 0.07 | 1.12 | (0.93, 1.35) | 0.2200 |
| rs602399 | 33821757 | 0.22 | 0.19 | 1.01 | (0.89, 1.15) | 0.8411 |
| rs4713675 | 33826396 | 0.45 | 0.46 | 0.98 | (0.89, 1.09) | 0.7766 |
| rs1536500 | 33831361 | 0.23 | 0.22 | 0.89 | (0.79, 1.00) | 0.0513 |
| rs1536501 | 33835863 | 0.22 | 0.19 | 0.99 | (0.87, 1.12) | 0.8407 |
| rs755495 | 33842106 | 0.25 | 0.25 | 0.98 | (0.88, 1.10) | 0.7848 |
| rs747694 | 33844468 | 0.47 | 0.47 | 0.99 | (0.90, 1.09) | 0.8626 |
| rs4711350 | 33849694 | 0.21 | 0.19 | 0.98 | (0.87, 1.11) | 0.8131 |
| rs2182658 | 33856809 | 0.27 | 0.22 | 0.97 | (0.86, 1.09) | 0.6660 |
| rs2395402 | 33861651 | 0.48 | 0.47 | 0.97 | (0.88, 1.07) | 0.5603 |
| rs756139 | 33863519 | 0.24 | 0.21 | 0.98 | (0.87, 1.11) | 0.7958 |
| rs2395401 | 33867449 | 0.21 | 0.20 | 0.94 | (0.83, 1.07) | 0.3724 |
| rs2274459 | 33870220 | 0.10 | 0.17 | 0.97 | (0.85, 1.10) | 0.6130 |
| rs751727 | 33872136 | 0.21 | 0.19 | 1.01 | (0.89, 1.14) | 0.9028 |
| rs3828783 | 33875705 | 0.19 | 0.20 | 0.92 | (0.81, 1.04) | 0.1764 |
| rs2281820 | 33876875 | 0.46 | 0.44 | 0.97 | (0.88, 1.07) | 0.5309 |
| rs943462 | 33877556 | 0.25 | 0.25 | 0.99 | (0.89, 1.11) | 0.9174 |
| rs2395400 | 33878054 | 0.25 | 0.24 | 0.95 | (0.84, 1.08) | 0.4403 |
| rs3806110 | 33878607 | 0.39 | 0.34 | 1.05 | (0.95, 1.17) | 0.3690 |
| rs2281816 | 33880313 | 0.33 | 0.28 | 1.01 | (0.90, 1.12) | 0.9282 |
| rs3763260 | 33882048 | 0.33 | 0.27 | 1.04 | (0.93, 1.16) | 0.5012 |
| rs1547668 | 33883424 | 0.21 | 0.21 | 0.95 | (0.84, 1.07) | 0.3969 |
