# Supplementary material for: High-Density SNP Screening of the Major Histocompatibility Complex in Systemic Lupus Erythematosus Demonstrates Strong Evidence for Independent Susceptibility Regions
Source: PLoS Genet. 2009 Oct 23;5(10):e1000696. doi: 10.1371/journal.pgen.1000696 (PMC2758598; doi:10.1371/journal.pgen.1000696)
Supplement: Table S3 — List of MHC SNPs (n = 171) associated with SLE with p<0.01 based on conditional haplotype method among 1,484 SLE cases and 650 controls. (0.16 MB DOC) [file pgen.1000696.s003.doc]

**Table S3.** List of MHC SNPs (n = 171) associated with SLE with p < 0.01 based on conditional haplotype method among 1,484 SLE cases and 650 controls.

| **SNP (rs#)** | **Position (base pair)** |
| --- | --- |
| rs1536215 | 28975894 |
| rs4452630 | 29466912 |
| rs2073154 | 29472794 |
| rs4711187 | 29475569 |
| rs362521 | 29664738 |
| rs29255 | 29687523 |
| rs715044 | 29701767 |
| rs2734985 | 29926641 |
| rs2523995 | 30210163 |
| rs2517646 | 30230554 |
| rs2523734 | 30237655 |
| rs6457164 | 30263087 |
| rs3132671 | 30286266 |
| rs3094132 | 30295413 |
| rs3094140 | 30304292 |
| rs1573295 | 30307046 |
| rs3130374 | 30429315 |
| rs3132636 | 30450491 |
| rs1264550 | 30485177 |
| rs1150769 | 30490951 |
| rs1264542 | 30494975 |
| rs1268445 | 30495446 |
| rs1269556 | 30516766 |
| rs1268444 | 30519525 |
| rs2844729 | 30530611 |
| rs2516677 | 30532602 |
| rs915666 | 30535461 |
| rs8512 | 30819336 |
| rs4713372 | 30878757 |
| rs2844657 | 30937501 |
| rs886422 | 30972258 |
| rs3132579 | 31048968 |
| rs3132556 | 31186788 |
| rs2233986 | 31187055 |
| rs3130982 | 31192054 |
| rs3094214 | 31193361 |
| rs3094204 | 31199971 |
| rs1265115 | 31225054 |
| rs7750641 | 31237289 |
| rs2923006 | 31425078 |
| rs2156875 | 31425326 |
| rs6933050 | 31451611 |
| rs3763288 | 31478346 |
| rs2516500 | 31561619 |
| rs2516415 | 31567721 |
| rs2516408 | 31571470 |
| rs2534679 | 31571769 |
| rs6916394 | 31572029 |
| rs3828903 | 31572718 |
| rs7382817 | 31578854 |
| rs2246618 | 31586965 |
| rs2246626 | 31587105 |
| rs2523503 | 31621538 |
| rs3093668 | 31654474 |
| rs2857595 | 31676448 |
| rs9348876 | 31683255 |
| rs3132450 | 31704117 |
| rs2077102 | 31719819 |
| rs15574 | 31794476 |
| rs3131379 | 31829012 |
| rs3117574 | 31833209 |
| rs3115671 | 31842324 |
| rs2471980 | 31908847 |
| rs644045 | 31991936 |
| rs497309 | 32000463 |
| rs1270942 | 32026839 |
| rs2072633 | 32027557 |
| rs440454 | 32035321 |
| rs592229 | 32038420 |
| rs389884 | 32048876 |
| rs389883 | 32055439 |
| rs1150753 | 32167845 |
| rs2071293 | 32170665 |
| rs2269426 | 32184477 |
| rs8283 | 32191278 |
| rs204999 | 32217957 |
| rs3134952 | 32221549 |
| rs3134608 | 32225949 |
| rs3130284 | 32248465 |
| rs3131297 | 32248983 |
| rs3134946 | 32253971 |
| rs3134945 | 32254470 |
| rs3132965 | 32254975 |
| rs3130349 | 32255674 |
| rs204994 | 32262976 |
| rs204993 | 32263559 |
| rs176095 | 32266297 |
| rs204991 | 32269344 |
| rs204990 | 32269408 |
| rs204989 | 32269830 |
| rs2071278 | 32273422 |
| rs3131296 | 32280971 |
| rs2071285 | 32288409 |
| rs45855 | 32297459 |
| rs365053 | 32303966 |
| rs382259 | 32317005 |
| rs3115573 | 32326821 |
| rs6908927 | 32332467 |
| rs3115560 | 32344120 |
| rs3096673 | 32345991 |
| rs3132945 | 32346658 |
| rs3115553 | 32353805 |
| rs6909427 | 32376679 |
| rs508805 | 32403328 |
| rs3129949 | 32406792 |
| rs1003878 | 32407800 |
| rs9366793 | 32409267 |
| rs6929776 | 32411489 |
| rs2273019 | 32414397 |
| rs2143462 | 32443182 |
| rs3129937 | 32444342 |
| rs3129939 | 32444744 |
| rs2050190 | 32447054 |
| rs3129944 | 32448850 |
| rs7746019 | 32450515 |
| rs3117103 | 32457535 |
| rs3129948 | 32462622 |
| rs743862 | 32489917 |
| rs3135380 | 32492655 |
| rs2395161 | 32495730 |
| rs3135363 | 32497626 |
| rs3135353 | 32500855 |
| rs2187818 | 32503546 |
| rs3135339 | 32507239 |
| rs2395172 | 32507820 |
| rs9268606 | 32508048 |
| rs3129858 | 32508498 |
| rs3129859 | 32508917 |
| rs983561 | 32511633 |
| rs2395177 | 32513054 |
| rs3129872 | 32515131 |
| rs2395181 | 32515382 |
| rs3129878 | 32516713 |
| rs3129881 | 32517462 |
| rs8084 | 32519013 |
| rs2239806 | 32519285 |
| rs7192 | 32519624 |
| rs7194 | 32520458 |
| rs2227139 | 32521437 |
| rs3129890 | 32522251 |
| rs7452076 | 32523058 |
| rs7766843 | 32538707 |
| rs9269043 | 32546576 |
| rs6901541 | 32550239 |
| rs3129763 | 32698903 |
| rs7744001 | 32734064 |
| rs2856718 | 32778233 |
| rs3916766 | 32789623 |
| rs11758312 | 32824350 |
| rs6918223 | 32830510 |
| rs7770024 | 32831319 |
| rs7769979 | 32831550 |
| rs6902723 | 32839938 |
| rs2857197 | 32854962 |
| rs10947345 | 32857773 |
| rs2621393 | 32863194 |
| rs7383433 | 32886509 |
| rs5009557 | 32887974 |
| rs10484565 | 32903010 |
| rs154986 | 32987211 |
| rs23544 | 33011615 |
| rs68600 | 33011702 |
| rs194675 | 33013724 |
| rs1480380 | 33021224 |
| rs2581 | 33082379 |
| rs399604 | 33082992 |
| rs453779 | 33083359 |
| rs2269346 | 33266876 |
| rs383711 | 33281976 |
| rs211453 | 33438109 |
| rs2247385 | 33529555 |
