# Supplementary material for: High-Density SNP Screening of the Major Histocompatibility Complex in Systemic Lupus Erythematosus Demonstrates Strong Evidence for Independent Susceptibility Regions
Source: PLoS Genet. 2009 Oct 23;5(10):e1000696. doi: 10.1371/journal.pgen.1000696 (PMC2758598; doi:10.1371/journal.pgen.1000696)
Supplement: Table S4 — Ancestry informative markers (total n = 357) genotyped to classify SLE cases (n = 1,570) according to continental ancestry (n = 112 SNPs) and European substructure (n = 246 SNPs). (0.39 MB DOC) [file pgen.1000696.s004.doc]

**Table S4**. Ancestry informative markers (total n = 357) genotyped to

classify SLE cases (n = 1,570) according to continental ancestry (n = 112

SNPs) and European substructure (n = 246 SNPs).

| **SNP** | **chromosome** | **Position, base pair** | **Ancestry information** |
| --- | --- | --- | --- |
| rs7548659 | 1 | 11030026 | intra-European |
| rs6541030 | 1 | 12530765 | continental |
| rs760607 | 1 | 23735240 | intra-European |
| rs1981135 | 1 | 26320326 | intra-European |
| rs4908343 | 1 | 27804285 | continental and intra-European |
| rs749663 | 1 | 30081331 | intra-European |
| rs1334804 | 1 | 37167831 | intra-European |
| rs1325502 | 1 | 42132857 | continental |
| rs1288367 | 1 | 53378472 | intra-European |
| rs213496 | 1 | 54642404 | intra-European |
| rs12130799 | 1 | 55435960 | continental |
| rs4915691 | 1 | 65640107 | intra-European |
| rs1566246 | 1 | 67017863 | intra-European |
| rs3118378 | 1 | 68622275 | continental |
| rs3101336 | 1 | 72523773 | intra-European |
| rs10493649 | 1 | 79980647 | intra-European |
| rs6660484 | 1 | 91945196 | intra-European |
| rs2968485 | 1 | 96642521 | intra-European |
| rs3737576 | 1 | 101482151 | continental |
| rs12061503 | 1 | 101615842 | intra-European |
| rs731756 | 1 | 110662638 | intra-European |
| rs7554936 | 1 | 149389113 | continental |
| rs1419074 | 1 | 163286625 | intra-European |
| rs1040404 | 1 | 166426514 | continental |
| rs488150 | 1 | 168418141 | intra-European |
| rs859362 | 1 | 173761713 | intra-European |
| rs1407434 | 1 | 184415655 | continental |
| rs2784101 | 1 | 197023984 | intra-European |
| rs10494878 | 1 | 204982432 | intra-European |
| rs4951629 | 1 | 210853506 | continental |
| rs1930903 | 1 | 227928109 | intra-European |
| rs2275302 | 1 | 230657489 | intra-European |
| rs316873 | 1 | 240409127 | continental |
| rs4916113 | 1 | 246443915 | intra-European |
| rs587913 | 2 | 4454758 | intra-European |
| rs798443 | 2 | 7885726 | continental |
| rs10929646 | 2 | 10302548 | intra-European |
| rs4260216 | 2 | 11452974 | intra-European |
| rs7421394 | 2 | 14673800 | continental |
| rs340747 | 2 | 16316196 | intra-European |
| rs1489688 | 2 | 18525734 | intra-European |
| rs4665797 | 2 | 21523281 | intra-European |
| rs13032262 | 2 | 25499825 | intra-European |
| rs10205008 | 2 | 28618813 | intra-European |
| rs4666200 | 2 | 29391915 | continental |
| rs605832 | 2 | 31308049 | intra-European |
| rs7594173 | 2 | 32753834 | intra-European |
| rs4670767 | 2 | 37794900 | continental |
| rs7577894 | 2 | 55862408 | intra-European |
| rs2075375 | 2 | 63134194 | intra-European |
| rs6720799 | 2 | 70225533 | intra-European |
| rs41420 | 2 | 72255762 | intra-European |
| rs13032535 | 2 | 75728825 | intra-European |
| rs13400937 | 2 | 79718431 | continental |
| rs11123861 | 2 | 97676314 | intra-European |
| rs6739285 | 2 | 104723749 | intra-European |
| rs260690 | 2 | 108946170 | continental |
| rs1955394 | 2 | 130339358 | intra-European |
| rs16829231 | 2 | 134248092 | intra-European |
| rs6730157 | 2 | 135623558 | intra-European |
| rs2117742 | 2 | 137765283 | intra-European |
| rs10496971 | 2 | 145486413 | continental |
| rs6752189 | 2 | 163857936 | intra-European |
| rs1227131 | 2 | 168121044 | intra-European |
| rs6749847 | 2 | 177276102 | intra-European |
| rs2627037 | 2 | 179314783 | continental |
| rs7558428 | 2 | 181726799 | intra-European |
| rs1569175 | 2 | 200730199 | continental |
| rs3769823 | 2 | 201831240 | intra-European |
| rs10189760 | 2 | 223552138 | intra-European |
| rs6436553 | 2 | 226002129 | intra-European |
| rs10510228 | 3 | 2183832 | continental |
| rs10510268 | 3 | 3539506 | intra-European |
| rs3804989 | 3 | 4703008 | intra-European |
| rs342042 | 3 | 6864799 | intra-European |
| rs3916092 | 3 | 19921061 | intra-European |
| rs4955316 | 3 | 30390616 | continental |
| rs744751 | 3 | 30710941 | intra-European |
| rs9809104 | 3 | 39121433 | continental |
| rs11544593 | 3 | 57278724 | intra-European |
| rs6773085 | 3 | 59447213 | intra-European |
| rs9310279 | 3 | 73793635 | intra-European |
| rs6548616 | 3 | 79482265 | continental |
| rs6551458 | 3 | 90355016 | intra-European |
| rs7630043 | 3 | 96097436 | intra-European |
| rs12629908 | 3 | 122005406 | continental |
| rs734873 | 3 | 149233045 | continental |
| rs10513729 | 3 | 176163941 | intra-European |
| rs7636818 | 3 | 180504809 | intra-European |
| rs2030763 | 3 | 181447421 | continental |
| rs4859259 | 3 | 184147241 | intra-European |
| rs1513181 | 3 | 190057690 | continental |
| rs6775595 | 3 | 190616271 | intra-European |
| rs9291090 | 4 | 5441538 | continental |
| rs6449375 | 4 | 18001717 | intra-European |
| rs13132286 | 4 | 20972739 | intra-European |
| rs6831024 | 4 | 25472473 | intra-European |
| rs6448770 | 4 | 31006008 | intra-European |
| rs9884706 | 4 | 32275985 | intra-European |
| rs10008492 | 4 | 38442115 | intra-European |
| rs12186184 | 4 | 40992187 | intra-European |
| rs10007810 | 4 | 41249121 | continental |
| rs1389037 | 4 | 45489650 | intra-European |
| rs6832891 | 4 | 54751401 | intra-European |
| rs1109501 | 4 | 71364079 | intra-European |
| rs1369093 | 4 | 73464055 | continental |
| rs3923243 | 4 | 75798477 | intra-European |
| rs6820697 | 4 | 85019499 | intra-European |
| rs385194 | 4 | 85528102 | continental |
| rs1343921 | 4 | 90166485 | intra-European |
| rs7669241 | 4 | 100649947 | intra-European |
| rs7657799 | 4 | 105594872 | continental |
| rs2194860 | 4 | 110167960 | intra-European |
| rs1448817 | 4 | 111860502 | intra-European |
| rs4574434 | 4 | 115958877 | intra-European |
| rs1541745 | 4 | 117710581 | intra-European |
| rs692157 | 4 | 122602284 | intra-European |
| rs1390009 | 4 | 135151781 | intra-European |
| rs10519410 | 4 | 138354031 | intra-European |
| rs1507500 | 4 | 148213152 | intra-European |
| rs2702414 | 4 | 179636517 | continental |
| rs1435442 | 4 | 185054196 | intra-European |
| rs10032784 | 4 | 190505753 | intra-European |
| rs2736122 | 5 | 1310621 | intra-European |
| rs316598 | 5 | 2417626 | continental |
| rs870347 | 5 | 6898035 | continental |
| rs37369 | 5 | 35072872 | continental |
| rs6451722 | 5 | 43747135 | continental |
| rs7718757 | 5 | 56965873 | intra-European |
| rs1533019 | 5 | 59343570 | intra-European |
| rs29460 | 5 | 65570159 | intra-European |
| rs2279095 | 5 | 76745182 | intra-European |
| rs1373967 | 5 | 80252312 | intra-European |
| rs6452788 | 5 | 87748669 | intra-European |
| rs174015 | 5 | 102159866 | intra-European |
| rs6866231 | 5 | 128831244 | intra-European |
| rs346650 | 5 | 135752820 | intra-European |
| rs1528961 | 5 | 136821047 | intra-European |
| rs6556352 | 5 | 155404292 | continental |
| rs1500127 | 5 | 165672560 | continental |
| rs7727897 | 5 | 176979615 | intra-European |
| rs6422347 | 5 | 177795689 | continental |
| rs248327 | 5 | 179310256 | intra-European |
| rs1933652 | 6 | 686043 | intra-European |
| rs2326106 | 6 | 2934581 | intra-European |
| rs1040045 | 6 | 4692158 | continental |
| rs4960257 | 6 | 6810243 | intra-European |
| rs2876167 | 6 | 9589358 | intra-European |
| rs2504853 | 6 | 12643097 | continental |
| rs1992387 | 6 | 13539293 | intra-European |
| rs7763768 | 6 | 17037691 | intra-European |
| rs7745461 | 6 | 22019595 | continental |
| rs6918101 | 6 | 25135267 | intra-European |
| rs12660883 | 6 | 30872399 | intra-European |
| rs382259 | 6 | 32317005 | intra-European |
| rs2076173 | 6 | 35550375 | intra-European |
| rs192655 | 6 | 90574999 | continental |
| rs4610536 | 6 | 91971586 | intra-European |
| rs638473 | 6 | 117306516 | intra-European |
| rs2451688 | 6 | 129688258 | intra-European |
| rs9389124 | 6 | 134355478 | intra-European |
| rs7753036 | 6 | 138953540 | intra-European |
| rs4463276 | 6 | 145097024 | continental |
| rs17551120 | 6 | 147275677 | intra-European |
| rs11756366 | 6 | 153893287 | intra-European |
| rs4458655 | 6 | 163141782 | continental |
| rs10428822 | 6 | 166523755 | intra-European |
| rs1871428 | 6 | 168408609 | continental |
| rs2189947 | 7 | 10285539 | intra-European |
| rs731257 | 7 | 12635776 | continental |
| rs10267453 | 7 | 14099830 | intra-European |
| rs697518 | 7 | 16725633 | intra-European |
| rs2529015 | 7 | 20293111 | intra-European |
| rs10951140 | 7 | 26498245 | intra-European |
| rs32314 | 7 | 32145649 | continental |
| rs2330442 | 7 | 42346596 | continental |
| rs10954737 | 7 | 83370983 | continental |
| rs7791143 | 7 | 89525200 | intra-European |
| rs705308 | 7 | 97533299 | continental |
| rs4730287 | 7 | 107557860 | intra-European |
| rs7781715 | 7 | 110500456 | intra-European |
| rs7803075 | 7 | 130392606 | continental |
| rs1593306 | 7 | 131264503 | intra-European |
| rs10236187 | 7 | 139093846 | continental |
| rs11761774 | 7 | 141507938 | intra-European |
| rs4571660 | 7 | 147356394 | intra-European |
| rs6464211 | 7 | 151504786 | continental |
| rs10108270 | 8 | 4178201 | continental |
| rs3943253 | 8 | 13403871 | continental |
| rs1548353 | 8 | 17211136 | intra-European |
| rs4565458 | 8 | 22210078 | intra-European |
| rs12548107 | 8 | 27113796 | intra-European |
| rs1471939 | 8 | 28997224 | continental |
| rs13273386 | 8 | 35765533 | intra-European |
| rs10109984 | 8 | 48966228 | intra-European |
| rs7830163 | 8 | 53240995 | intra-European |
| rs6993747 | 8 | 54343468 | intra-European |
| rs7827918 | 8 | 60640671 | intra-European |
| rs4634634 | 8 | 74431380 | intra-European |
| rs11784678 | 8 | 80450772 | intra-European |
| rs1026804 | 8 | 81680875 | intra-European |
| rs12544346 | 8 | 86611868 | continental |
| rs1841316 | 8 | 105911442 | intra-European |
| rs4871195 | 8 | 122567434 | intra-European |
| rs7844723 | 8 | 122977684 | continental |
| rs4736413 | 8 | 133395104 | intra-European |
| rs2001907 | 8 | 140310363 | continental |
| rs10758823 | 9 | 7078043 | intra-European |
| rs1408801 | 9 | 12662320 | continental |
| rs10962589 | 9 | 16773380 | intra-European |
| rs10812520 | 9 | 27037246 | intra-European |
| rs10511828 | 9 | 28618500 | continental |
| rs867469 | 9 | 32373708 | intra-European |
| rs3793451 | 9 | 70849100 | continental |
| rs2130118 | 9 | 75533109 | intra-European |
| rs2306040 | 9 | 92681020 | continental |
| rs10739277 | 9 | 111091779 | intra-European |
| rs7860625 | 9 | 115104908 | intra-European |
| rs10513300 | 9 | 119170027 | continental |
| rs2073821 | 9 | 134922943 | continental |
| rs1009473 | 9 | 137917133 | intra-European |
| rs7090242 | 10 | 1628527 | intra-European |
| rs7921493 | 10 | 7379070 | intra-European |
| rs1324322 | 10 | 10612757 | intra-European |
| rs3793791 | 10 | 50511710 | continental |
| rs3858126 | 10 | 65381692 | intra-European |
| rs4746826 | 10 | 70655656 | intra-European |
| rs4746136 | 10 | 74971000 | continental |
| rs4691 | 10 | 75231922 | intra-European |
| rs499437 | 10 | 78663949 | intra-European |
| rs1857459 | 10 | 85674575 | intra-European |
| rs7097946 | 10 | 90635321 | intra-European |
| rs11186543 | 10 | 93109747 | intra-European |
| rs1023331 | 10 | 97756527 | intra-European |
| rs10509826 | 10 | 108794830 | intra-European |
| rs1325172 | 10 | 114348545 | intra-European |
| rs4918842 | 10 | 115306802 | continental |
| rs11146457 | 10 | 134332339 | intra-European |
| rs4880436 | 10 | 134500093 | continental |
| rs10839880 | 11 | 7806892 | continental |
| rs1837606 | 11 | 15794713 | continental |
| rs2045272 | 11 | 18234674 | intra-European |
| rs7931276 | 11 | 21606387 | intra-European |
| rs2946788 | 11 | 23967106 | continental |
| rs2218868 | 11 | 57682553 | intra-European |
| rs2237997 | 11 | 60528666 | intra-European |
| rs12804561 | 11 | 62673153 | intra-European |
| rs11227699 | 11 | 66655068 | continental |
| rs488753 | 11 | 103481503 | intra-European |
| rs7131355 | 11 | 115582580 | intra-European |
| rs948028 | 11 | 120149657 | continental |
| rs4936969 | 11 | 124395606 | intra-European |
| rs740851 | 12 | 6508611 | intra-European |
| rs7976721 | 12 | 7824902 | intra-European |
| rs2416791 | 12 | 11592755 | continental |
| rs1513056 | 12 | 17299059 | continental |
| rs699039 | 12 | 25055598 | intra-European |
| rs12370505 | 12 | 32981222 | intra-European |
| rs214678 | 12 | 45963217 | continental |
| rs772262 | 12 | 54450001 | continental |
| rs2860493 | 12 | 67209117 | intra-European |
| rs2723891 | 12 | 89482389 | intra-European |
| rs11107018 | 12 | 92338033 | intra-European |
| rs12231308 | 12 | 96041305 | intra-European |
| rs7312155 | 12 | 105734583 | intra-European |
| rs2070586 | 12 | 107801849 | continental |
| rs2650170 | 12 | 116506138 | intra-European |
| rs3815210 | 12 | 119064052 | intra-European |
| rs377318 | 13 | 21784101 | intra-European |
| rs9319336 | 13 | 26522356 | continental |
| rs7997709 | 13 | 33745737 | continental |
| rs7323018 | 13 | 43513039 | intra-European |
| rs9530435 | 13 | 74891888 | continental |
| rs1678386 | 13 | 94647177 | intra-European |
| rs9556553 | 13 | 95754094 | intra-European |
| rs9522149 | 13 | 110625168 | continental |
| rs1760921 | 14 | 19887971 | continental |
| rs4982420 | 14 | 20705711 | intra-European |
| rs2038281 | 14 | 38984948 | intra-European |
| rs2357442 | 14 | 51677717 | continental |
| rs10498472 | 14 | 54424619 | intra-European |
| rs8021730 | 14 | 66956534 | continental |
| rs1531631 | 14 | 78694245 | intra-European |
| rs946918 | 14 | 82542621 | continental |
| rs17126387 | 14 | 89371067 | intra-European |
| rs9671457 | 14 | 94086589 | intra-European |
| rs1947745 | 15 | 25540093 | intra-European |
| rs2611605 | 15 | 30228925 | intra-European |
| rs12439433 | 15 | 34007327 | continental |
| rs1153860 | 15 | 43423876 | intra-European |
| rs1906433 | 15 | 51666240 | intra-European |
| rs10519005 | 15 | 57498983 | intra-European |
| rs2292745 | 15 | 66411344 | intra-European |
| rs2899826 | 15 | 72521553 | continental |
| rs8035124 | 15 | 89906712 | continental |
| rs1510058 | 15 | 97672532 | intra-European |
| rs1891325 | 16 | 6372409 | intra-European |
| rs4781011 | 16 | 10882812 | continental |
| rs949429 | 16 | 11283530 | intra-European |
| rs30237 | 16 | 14282146 | intra-European |
| rs8047148 | 16 | 22282331 | intra-European |
| rs7205880 | 16 | 25758692 | intra-European |
| rs818386 | 16 | 63964209 | continental |
| rs3751834 | 16 | 77573909 | intra-European |
| rs2966849 | 16 | 83741183 | continental |
| rs6539986 | 16 | 85526711 | intra-European |
| rs1879488 | 17 | 1348363 | continental |
| rs7215135 | 17 | 9105273 | intra-European |
| rs11870879 | 17 | 45102647 | intra-European |
| rs2033111 | 17 | 51143279 | continental |
| rs12939848 | 17 | 62801270 | intra-European |
| rs8071270 | 17 | 66907543 | intra-European |
| rs10512572 | 17 | 67023694 | continental |
| rs2125345 | 17 | 71293786 | continental |
| rs736632 | 18 | 5963728 | intra-European |
| rs400839 | 18 | 8650149 | intra-European |
| rs4798812 | 18 | 9410504 | continental |
| rs4800105 | 18 | 17905980 | continental |
| rs1786153 | 18 | 20752168 | intra-European |
| rs11564361 | 18 | 24158866 | intra-European |
| rs16948113 | 18 | 26308259 | intra-European |
| rs7238445 | 18 | 48035542 | continental |
| rs881728 | 18 | 57484088 | continental |
| rs17079195 | 18 | 64288874 | intra-European |
| rs4891825 | 18 | 66018643 | continental |
| rs11151863 | 18 | 69270859 | intra-European |
| rs874299 | 18 | 73185272 | continental |
| rs4536588 | 19 | 724437 | intra-European |
| rs1552046 | 19 | 5073051 | intra-European |
| rs715159 | 19 | 14900519 | intra-European |
| rs8111998 | 19 | 22533515 | intra-European |
| rs2082455 | 19 | 34699475 | intra-European |
| rs17496703 | 19 | 36476435 | intra-European |
| rs8113143 | 19 | 38344087 | continental |
| rs7976 | 19 | 40670139 | intra-European |
| rs7252868 | 19 | 45042569 | intra-European |
| rs3745099 | 19 | 57593717 | continental |
| rs6076623 | 20 | 4084671 | intra-European |
| rs6104567 | 20 | 10143433 | continental |
| rs1041200 | 20 | 10927521 | intra-European |
| rs8116153 | 20 | 13182124 | intra-European |
| rs1884783 | 20 | 18673724 | intra-European |
| rs6126462 | 20 | 35958790 | intra-European |
| rs6129532 | 20 | 38317602 | intra-European |
| rs6032343 | 20 | 43637704 | intra-European |
| rs477627 | 20 | 47613465 | intra-European |
| rs2041317 | 20 | 51797014 | intra-European |
| rs6092326 | 20 | 54550144 | intra-European |
| rs3916504 | 20 | 57551979 | intra-European |
| rs6071491 | 20 | 59117976 | intra-European |
| rs977712 | 21 | 18992561 | intra-European |
| rs1735899 | 21 | 20734486 | intra-European |
| rs2835370 | 21 | 36807495 | continental |
| rs11702531 | 21 | 40399077 | intra-European |
| rs1296819 | 22 | 16456546 | continental |
| rs4821004 | 22 | 30696359 | continental |
| rs2235338 | 22 | 36295826 | intra-European |
