# Supplementary material for: High-Density SNP Screening of the Major Histocompatibility Complex in Systemic Lupus Erythematosus Demonstrates Strong Evidence for Independent Susceptibility Regions
Source: PLoS Genet. 2009 Oct 23;5(10):e1000696. doi: 10.1371/journal.pgen.1000696 (PMC2758598; doi:10.1371/journal.pgen.1000696)
Supplement: Table S5 — Results from analyses of independently associated MHC variants in SLE families and Northern European individuals (see Figure 3 and Figure 4). (0.04 MB DOC) [file pgen.1000696.s005.doc]

**Table S5.** Results from analyses of independently associated MHC variants in SLE families and Northern European

individuals (See Figures 3 and 4).

| **Rs#** | **location** | **Gene** | **OR*** | **95%CI*** | **p-value*** | **TDT^†^**  **p-value** | **N.Euro^‡^**  **p-value** |
| --- | --- | --- | --- | --- | --- | --- | --- |
| rs1536215 | 28975894 | *TRIM27* | 0.77 | (0.66, 0.90) | 0.0009 | 0.0009 | 0.0013 |
| rs362521 | 29664738 | *OR2H2* | 0.69 | (0.56, 0.87) | 0.0010 | 0.0016 | 0.0001 |
| rs3828903 | 31572718 | *MICB* | 0.78 | (0.70, 0.87) | 8.15E-06 | 1.69E-08 | 0.0001 |
| rs2246626 | 31587105 | *MICB* | 0.86 | (0.78, 0.95) | 0.0032 | 1.45E-05 | 0.0085 |
| rs8283 | 32191278 | *CREBL1* | 0.74 | (0.66, 0.84) | 3.82E-06 | 3.45E-06 | 3.76E-06 |
| rs7746019 | 32450515 | *C6orf10 (nearest)* | 1.44 | (1.29, 1.61) | 8.80E-11 | 1.32E-08 | 3.57E-12 |
| rs3117103 | 32457535 | *C6orf10 (nearest)* | 1.63 | (1.43, 1.85) | 2.80E-13 | 1.58E-14 | 1.62E-13 |
| rs7769979 | 32831550 | *HLA-DQB2 (nearest)* | 0.72 | (0.65, 0.79) | 1.35E-11 | 2.10E-11 | 2.47E-13 |
| rs10947345 | 32857773 | *HLA-DQB2 (nearest)* | 1.17 | (1.05, 1.29) | 0.0033 | 0.0127 | 0.0021 |
| rs383711 | 33281976 | *HSD117B8* | 0.71 | (0.58, 0.87) | 0.0008 | 0.0006 | 0.0428 |

* Results from original single marker analyses (supplementary Table 2). OR = odds ratio, C.I. = confidence interval.

† TDT = transmission disequilibrium test

‡ Based on analysis of subjects with ≥ 90% Northern (vs. Southern) European ancestry estimated from ancestry informative marker.
