# Supplementary material for: High-Density SNP Screening of the Major Histocompatibility Complex in Systemic Lupus Erythematosus Demonstrates Strong Evidence for Independent Susceptibility Regions
Source: PLoS Genet. 2009 Oct 23;5(10):e1000696. doi: 10.1371/journal.pgen.1000696 (PMC2758598; doi:10.1371/journal.pgen.1000696)
Supplement: Table S6 — Results from relative predispositional effects (RPE) (A) and CHM analyses (B) of top MHC SNPs in SLE cases and controls. (Same as Table 3 and Table 4 with 95% confidence intervals included.) (0.08 MB DOC) [file pgen.1000696.s006.doc]

**Table S6**. Results from relative predispositional effects (RPE) (A) and CHM analyses (B) of top MHC SNPs in SLE cases and controls.* (Same as Tables 3 and 4 in manuscript with 95% confidence intervals included)

A.

| **SNP** | **Gene** | **All Haps**^†^ |  |  | ***DRB1*03* removed** |  |  | ***DRB1*03, *15* removed** |  |  | ***DRB1*03, *15, *14***  **removed** |  |  |
| --- | --- | --- | --- | --- | --- | --- | --- | --- | --- | --- | --- | --- | --- |
|  |  | **P-value** | **OR** | **95% CI** | **P-value** | **OR** | **95% CI** | **P-value** | **OR** | **95% CI** | **P-value** | **OR** | **95% CI** |
| rs1536215 | *TRIM27* | 0.0037 | 0.72 | (0.57, 0.90) | 0.0806 | 0.81 | (0.64, 1.02) | 0.0627 | 0.78 | (0.60, 1.00 | 0.0684 | 0.78 | (0.60, 1.01) |
| rs362521 | *OR2H2* | 0.0021 | 0.62 | (0.46, 0.84) | 0.0065 | 0.64 | (0.47, 0.88) | 0.0081 | 0.61 | (0.43, 0.87) | 0.0060 | 0.60 | (0.42, 0.86) |
| rs3828903 | *MICB* | 3.20 x 10^-7^ | 0.67 | (0.58, 0.78) | 0.0024 | 0.78 | (0.67, 0.92) | 0.0759 | 0.86 | (0.72, 1.02) | 0.0965 | 0.86 | (0.73, 1.03) |
| rs2246626 | *MICB* | 3.45 x 10^-4^ | 0.77 | (0.67, 0.89) | 0.2570 | 0.91 | (0.78, 1.07) | 0.7664 | 1.04 | (0.87, 1.21) | 0.6680 | 1.04 | (0.88, 1.23) |
| rs8283 | *CREBL1* | 7.67 x 10^-7^ | 0.63 | (0.53, 0.76) | 0.0013 | 0.74 | (0.61, 0.89) | 0.0117 | 0.78 | (0.65, 0.95) | 0.0111 | 0.78 | (0.64, 0.94) |
| rs7746019 | *C6orf10* | 7.85 x 10^-12^ | 1.77 | (1.50, 2.10) | 0.1075 | 1.20 | (0.96, 1.51) | 0.0244 | 1.30 | (1.03, 1.63) | 0.0224 | 1.31 | (1.04, 1.65) |
| rs3117103 | *C6orf10* | 2.42 x 10^-16^ | 2.35 | (1.89, 2.92) | 0.3961 | 1.47 | (0.71, 3.01) | 0.3701 | 1.52 | (0.71, 3.26) | 0.3699 | 1.50 | (0.70, 3.22) |
| rs7769979 | *DQB2* | 5.64 x 10^-14^ | 0.58 | (0.50, 0.67) | 1.90 x 10^-6^ | 0.69 | (0.59, 0.80) | 0.0013 | 0.75 | (0.63, 0.89) | 0.0030 | 0.76 | (0.64, 0.91) |
| rs10947345 | *DQB2* | 6.96 x 10^-4^ | 1.29 | (1.11, 1.50) | 1.21 x 10^-7^ | 1.53 | (1.31, 1.79) | 0.0002 | 1.42 | (1.18, 1.70) | 0.0004 | 1.40 | (1.16, 1.68) |
| rs383711 | *HSD17B8* | 0.0024 | 0.65 | (0.49, 0.85) | 0.0308 | 0.73 | (0.55, 0.97) | 0.0949 | 0.77 | (0.57, 1.04) | 0.1082 | 0.77 | (0.57, 1.05) |

B.

| **SNP** | **Gene** | ***DRB1*03***  **only** |  |  | ***DRB1*15***  **only** |  |  | ***DRB1*14***  ***only*** |  |  |
| --- | --- | --- | --- | --- | --- | --- | --- | --- | --- | --- |
|  |  | **P-value** | **OR** | **95% CI** | **P-value** | **OR** | **95% CI** | **P-value** | **OR** | **95% CI** |
| rs1536215 | *TRIM27* | 0.16 | 0.48 | (0.19, 1.20) | 0.7701 | 0.91 | (0.52, 1.61) | 1.000 | 0.89 | (0.19, 4.14) |
| rs362521 | *OR2H2* | 0.43 | 0.68 | (0.27, 1.74) | 0.4213 | 0.72 | (0.34, 1.52) | NA | NA | NA |
| rs3828903 | *MICB* | 0.0059 | 0.46 | (0.27, 0.79) | 0.1216 | 0.61 | (0.32, 1.13) | 0.3422 | 0.47 | (0.13, 1.68) |
| rs2246626 | *MICB* | 0.0615 | 0.59 | (0.34, 1.02) | 0.4478 | 0.80 | (0.45, 1.41) | 0.3614 | 0.48 | (0.14, 1.63) |
| rs8283 | *CREBL1* | 0.0122 | 0.32 | (0.14, 0.76) | 1.0000 | 1.02 | (0.36, 2.85) | 0.2220 | NA | NA |
| rs7746019 | *C6orf10* | 0.0189 | 1.94 | (1.13, 3.31) | 0.6853 | 2.05 | (0.24, 17.14) | 0.7403 | 1.40 | (0.37, 5.35) |
| rs3117103 | *C6orf10* | 0.0189 | 1.94 | (1.13, 3.31) | 1.0000 | 1.36 | (0.15, 12.25) | NA | NA | NA |
| rs7769979 | *DQB2* | 0.0544 | 0.54 | (0.30, 0.98) | 0.2374 | 0.70 | (0.41, 1.21) | 0.2451 | 0.30 | (0.05, 1.68) |
| rs10947345 | *DQB2* | 0.8908 | 1.06 | (0.61, 1.84) | 0.2536 | 1.37 | (0.80, 2.34) | 0.2451 | 3.35 | (0.60, 18.89) |
| rs383711 | *HSD17B8* | 0.2428 | 0.43 | (0.13, 1.44) | 0.2013 | 0.59 | (0.28, 1.28) | 0.5988 | 0.43 | (0.04, 5.14) |

*See Methods for details.

^†^Haplotypes assigned in SLE cases and all family members using PHASE v. 2.1.2. Non-transmitted haplotypes were used as control group.
