# Supplementary material for: High-Density SNP Screening of the Major Histocompatibility Complex in Systemic Lupus Erythematosus Demonstrates Strong Evidence for Independent Susceptibility Regions
Source: PLoS Genet. 2009 Oct 23;5(10):e1000696. doi: 10.1371/journal.pgen.1000696 (PMC2758598; doi:10.1371/journal.pgen.1000696)
Supplement: Table S7 — Details for top associated study MHC SNPs plus variants tagged by these SNPs (see Figure 3, Figure 4, Table 3, Table 4). (0.15 MB DOC) [file pgen.1000696.s007.doc]

**Table S7.** Details for top associated study MHC SNPs plus variants tagged by these SNPs (see Figures 3 and 4, Tables 3 and 4)

| Rs number | Tagged variants | position | Nearest Gene | Observed | Avg. Het.^1^ | Avg. Het. (SE) ^2^ | Function^3^ | Comments |
| --- | --- | --- | --- | --- | --- | --- | --- | --- |
| **rs362521** |  | 29664738 | *OR2H2* | G/A | 0.11 | 0.21 | near 3’ end | Typed phase III only Hap Map |
| rs362521 | rs12530421 | 29657422 | *GABBR1* | A/C | 0.16 | 0.23 | unknown |  |
| rs362521 | rs3025646 | 29674821 | *GABBR1* | A/G | 0.11 | 0.20 | unknown |  |
| rs362521 | rs3025645 | 29675600 | *GABBR1* | A/T | 0.11 | 0.21 | unknown |  |
| **rs3828903** |  | 31572718 | *MICB* | G/A | 0.47 | 0.12 | near 5’ end | Typed phase II and III HapMap |
| rs3828903 | rs9267356 | 31571312 | *MICB* | A/G | 0.00 | 0.00 | unknown |  |
| rs3828903 | rs6916187 | 31571905 | *MICB* | C/T | 0.47 | 0.13 | unknown |  |
| rs3828903 | rs3828905 | 31572881 | *MICB* | A/G | 0.47 | 0.12 | near 5’ end |  |
| rs3828903 | rs3828912 | 31573705 | *MICB* | A/C | 0.46 | 0.13 | near 5’ end |  |
| rs3828903 | rs3828914 | 31573798 | *MICB* | C/T | 0.47 | 0.13 | near 5’ end |  |
| rs3828903 | rs9267390 | 31576733 | *MICB* | A/G | 0.48 | 0.10 | intron |  |
| rs3828903 | rs9267404 | 31578524 | *MICB* | G/T | 0.47 | 0.12 | intron |  |
| rs3828903 | rs4713468 | 31578854 | *MICB* | A/T | 0.47 | 0.13 | intron |  |
| rs3828903 | rs9267415 | 31579809 | *MICB* | A/G | 0.47 | 0.13 | intron, near 5’ end |  |
| **rs8283** | none | 32191278 | *CREBL1* | A/G | 0.43 | 0.17 | 3’-UTR | Typed phase II and III HapMap |
| **rs7769979** |  | 32831550 | *HLA-DQB2* | G/A | 0.49 | 0.06 | unknown | Typed phase II and III HapMap |
| rs7769979 | rs9272775 | 32718235 | *HLA-DQA1* | C/T | 0.50 | 0.02 | intron |  |
| rs7769979 | rs12177980 | 32794062 | *LOC731881* | A/G | 0.48 | 0.09 | near 5’ end |  |
| rs7769979 | rs9469228 | 32795868 | *HLA_DQA2* | A/G | 0.48 | 0.10 | unknown |  |
| rs7769979 | rs9461799 | 32797507 | *HLA_DQB2* | A/G | 0.49 | 0.06 | unknown |  |
| rs7769979 | rs9469240 | 32797979 | *HLA_DQB2* | A/G | 0.49 | 0.06 | unknown |  |
| rs7769979 | rs2395237 | 32798923 | *HLA-DQA2* | C/T | 0.48 | 0.09 | unknown |  |
| rs7769979 | rs9469246 | 32800205 | *HLA-DQA2* | G/T | 0.49 | 0.07 | unknown |  |
| rs7769979 | rs2859064 | 32800790 | *HLA-DQA2* | A/G | 0.48 | 0.09 | unknown |  |
| rs7769979 | rs2858892 | 32801220 | *HLA-DQA2* | C/T | 0.48 | 0.09 | unknown |  |
| rs7769979 | rs2859118 | 32805318 | *HLA-DQA2* | C/T | 0.49 | 0.09 | unknown |  |
| rs7769979 | rs2859112 | 32805991 | *HLA-DQA2* | A/G | 0.48 | 0.09 | unknown |  |
| rs7769979 | rs2859109 | 32806185 | *HLA-DQA2* | C/T | 0.48 | 0.09 | unknown |  |
| rs7769979 | rs9469257 | 32807194 | *HLA-DQA2* | A/G | 0.48 | 0.09 | unknown |  |
| rs7769979 | rs6457643 | 32812392 | *HLA-DQA2* | G/T | 0.49 | 0.05 | unknown |  |
| rs7769979 | rs10947336 | 32812552 | *HLA-DQA2* | A/C | 0.50 | 0.03 | unknown |  |
| rs7769979 | rs12183007 | 32812608 | *HLA-DQA2* | A/G | 0.50 | 0.03 | unknown |  |
| rs7769979 | rs13199553 | 32813082 | *HLA-DQA2* | C/T | 0.50 | 0.02 | unknown |  |
| rs7769979 | rs13214143 | 32813105 | *HLA-DQA2* | A/G | 0.50 | 0.03 | unknown |  |
| rs7769979 | rs7773407 | 32814376 | *HLA-DQA2* | A/C | 0.50 | 0.02 | unknown |  |
| rs7769979 | rs7773441 | 32814473 | *HLA-DQA2* | A/T | 0.50 | 0.03 | unknown |  |
| rs7769979 | rs2894287 | 32815260 | *HLA-DQA2* | A/G | 0.50 | 0.03 | near 5’ end |  |
| rs7769979 | rs2227127 | 32819760 | *HLA-DQA2* | C/T | 0.49 | 0.06 | intron |  |
| rs7769979 | rs2071799 | 32822153 | *HLA-DQA2* | A/G | 0.50 | 0.05 | 3’-UTR |  |
| rs7769979 | rs28693951 | 32822710 | *HLA-DQA2* | C/T | 0.5 | 0.02 | 3’-UTR |  |
| rs7769979 | rs28420297 | 32822738 | *HLA-DQA2* | A/G | 0.5 | 0.02 | 3’-UTR |  |
| rs7769979 | rs5016066 | 32822796 | *HLA-DQA2* | A/C | 0.48 | 0.09 | unknown |  |
| rs7769979 | rs10947340 | 32826365 | *HLA-DQA2* | G/T | 0.50 | 0.02 | unknown |  |
| rs7769979 | rs10947342 | 32828758 | *HLA-DQB2* | A/G | 0.50 | 0.002 | unknown |  |
| rs7769979 | rs7450989 | 32829021 | *HLA-DQB2* | C/T | 0.50 | 0.03 | unknown |  |
| rs7769979 | rs7774435 | 32829926 | *HLA-DQB2* | C/T | 0.50 | 0.01 | unknown |  |
| rs7769979 | rs10807113 | 3.3E+07 | *HLA-DQB2* | A/C | 0.5 | 0.01 | unknown |  |
| rs7769979 | rs6934041 | 32830745 | *HLA-DQB2* | C/G | 0.49 | 0.05 | unknown |  |
| rs7769979 | rs6913646 | 32830809 | *HLA-DQB2* | C/T | 0.50 | 0.02 | unknown |  |
| rs7769979 | rs7769974 | 32831496 | *HLA-DQB2* | C/T | 0.50 | 0.03 | unknown |  |
| rs7769979 | rs7774452 | 32831847 | *HLA-DQB2* | C/T | 0.5 | 0.01 | unknown |  |
| rs7769979 | rs7756516 | 32831895 | *HLA-DQB2* | C/T | 0.5 | 0.02 | unknown |  |
| rs7769979 | rs9296042 | 32843983 | *HLA-DQB2* | C/T | 0.50 | 0.06 | unknown |  |
| **rs10947345** |  | 32857773 | *HLA-DQB2* | T/A | 0.46 | 0.14 | unknown | Typed phase II only HapMap – SNP exists only in Build 35 |
| rs10947345 | rs7383119 | 32855505 | *HLA-DQB2* | A/G | na | na | unknown |  |
| rs10947345 | rs7450288 | 32858295 | *HLA-DQB2* | C/G | na | na | unknown |  |
| rs10947345 | rs2261566 | 32862058 | *HLA-DQB2* | A/T | 0.46 | 0.13 | unknown |  |
| rs10947345 | rs10947352 | 32864216 | *HLA-DQB2* | A/C | na | na | unknown |  |
| rs10947345 | rs9276705 | 32864810 | *HLA-DOB* | C/T | 0.46 | 0.14 | unknown |  |
| rs10947345 | rs2621389 | 32865509 | *HLA-DOB* | G/T | 0.46 | 0.14 | unknown |  |
| rs10947345 | rs2621387 | 32865948 | *HLA-DOB* | G/T | 0.46 | 0.14 | unknown |  |
| rs10947345 | rs2857164 | 32866196 | *HLA-DOB* | G/T | 0.46 | 0.14 | unknown |  |
| rs10947345 | rs2621386 | 32866245 | *HLA-DOB* | G/T | 0.46 | 0.14 | unknown |  |
| rs10947345 | rs1158785 | 32866548 | *HLA-DOB* | A/G | 0.46 | 0.14 | unknown |  |
| rs10947345 | rs2261684 | 32866644 | *HLA-DOB* | A/T | 0.47 | 0.13 | unknown |  |
| rs10947345 | rs2621384 | 32867250 | *HLA-DOB* | A/G | 0.45 | 0.16 | unknown |  |
| rs10947345 | rs2857161 | 32867274 | *HLA-DOB* | A/G | 0.45 | 0.16 | unknown |  |
| rs10947345 | rs2621383 | 32867312 | *HLA-DOB* | G/T | 0.45 | 0.15 | unknown |  |
| rs10947345 | rs2621382 | 32868422 | *HLA-DOB* | A/C | 0.46 | 0.13 | unknown |  |
| rs10947345 | rs2157082 | 32868691 | *HLA-DOB* | A/C | 0.47 | 0.13 | unknown |  |
| rs10947345 | rs2857153 | 32870618 | *HLA-DOB* | A/G | 0.46 | 0.13 | unknown |  |
| rs10947345 | rs2621379 | 32870769 | *HLA-DOB* | C/T | 0.46 | 0.13 | unknown |  |

^1^ Avg. Het. = average heterozygosity (http://genome.ucsc.edu)

^2^ Avg. Het. (SE) = standard error of average heterozygosity (http://genome.ucsc.edu)

^3^ Predicted functional role based on University of California, Santa Cruz genome browser http://genome.ucsc.edu/. UTR = untranslated region.
